# Differences in patient-centered burdens and economic outcomes based on sociodemographic characteristics and social determinants of health: a scoping review

**DOI:** 10.3389/fpubh.2026.1784571

**Published:** 2026-07-21

**Authors:** Sophia D’Angelo, Sydney Kirsch, Ella Shenkar, Olga Khavjou, Kristen Giombi

**Affiliations:** Center for Health Economics, Methods and Evidence Synthesis, RTI International, Durham, NC, United States

**Keywords:** caregiving burden, patient burden, patient-centered burdens and economic outcomes, social determinants of health, sociodemographic characteristics

## Abstract

**Background:**

We conducted a scoping review to examine how patient-centered burdens and economic outcomes (PCBEOs) vary across sociodemographic characteristics and social determinants of health (SDoH) among U.S. patients and their families or caregivers. PCBEOs include financial, emotional, physical, and time-related challenges associated with accessing medical care. We categorized these burdens into four domains: direct medical costs, direct non-medical costs, indirect impacts, and intangible burdens.

**Methods:**

We searched PubMed, CINAHL, EconLit, and Web of Science (January 2015–January 2025) for studies focused on adult patients and/or their families or caregivers who reported PCBEOs stratified by sociodemographic characteristics or SDoH. We categorized PCBEOs by domain, catalogued measurement tools and data sources, and summarized proposed strategies to mitigate PCBEOs.

**Results:**

From 1,461 records identified, we included 71 studies. Most studies focused on patients (*n* = 51), with others including caregivers (*n* = 14) or both groups (*n* = 6). Cancer was the most frequently studied medical condition (*n* = 41). Researchers primarily used cross-sectional designs (*n* = 64), and most relied on secondary analyses of survey data (*n* = 37). Intangible burdens (*n* = 55) were the most frequently reported domain, followed by direct medical costs (*n* = 27), indirect impacts (*n* = 17), and direct non-medical costs (*n* = 9). Common subcategories included financial toxicity, delayed or forgone care, and medical financial debt. Younger adults, lower-income households, individuals with less education, and Black or Hispanic populations more frequently reported greater PCBEOs. Fifteen studies proposed strategies to reduce differences in PCBEOs, including cost-related communication, insurance reform, transportation support, financial navigation, and patient-focused interventions.

**Conclusion:**

PCBEOs are widespread and unevenly distributed across U.S. populations. Our findings emphasize the need for standardized measurement tools, intersectional research approaches, and direct engagement with patients and caregivers to inform policies and interventions to reduce differences in PCBEOs and support sustainable and accessible care.

## 1 Introduction

Patients and their families or caregivers face significant physical, emotional, financial, and time-related burdens associated with accessing medical care (1). In the United States, average annual out-of-pocket (OOP) health care spending reached $1,425 per person in 2022 (2). People with high health care needs tend to incur the highest OOP spending: those in the top 1% of OOP spending pay an average of $23,700 out of pocket annually, and those in the top 10% pay $6,126, on average (2). In addition to these direct costs, patients often encounter less visible, yet equally impactful challenges related to economic burdens of health care such as emotional distress, lifestyle disruptions, financial hardship, and missed opportunities for social and economic participation (3, 4). Researchers collectively refer to these challenges as patient-centered burdens and economic outcomes (PCBEOs) (1).

**Figure 1:**
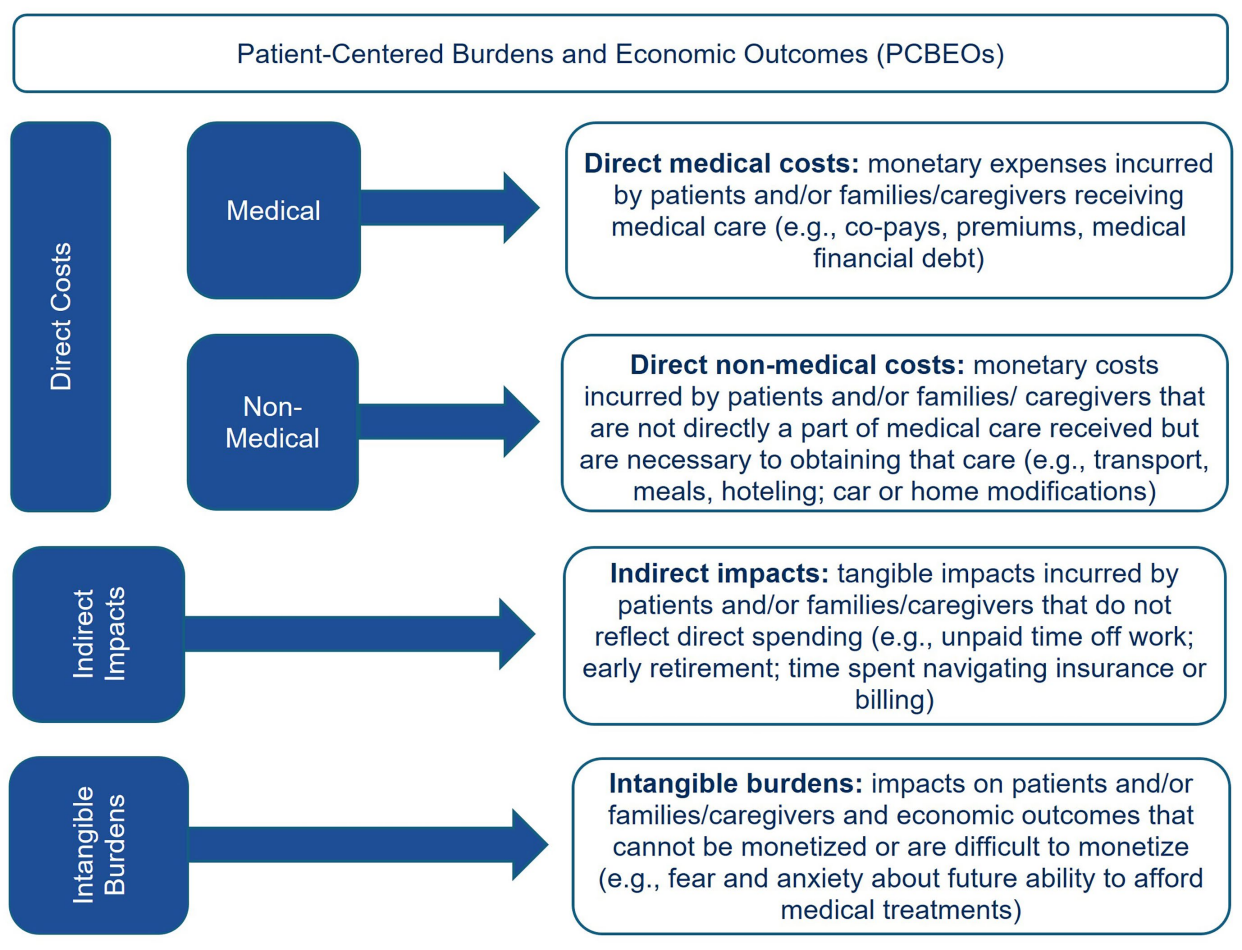
Categories of patient-centered burdens and economic outcomes. Note: This figure is based on Figure 2 from the PCORI PCBEO landscape review (1).

The Patient-Centered Outcomes Research Institute (PCORI) PCBEOs Landscape report describes four categories of PCBEOs (1), as outlined in Figure 1. These include direct medical costs (e.g., copayments, premiums, deductibles), direct non-medical costs (e.g., transportation and parking fees), indirect impacts (e.g., time spent attending appointments), and intangible burdens (e.g., anxiety about future affordability of care).

These burdens are widespread across the United States and may be intensified by sociodemographic characteristics and social determinants of health (SDoH), such as age, education, geographic location, income, insurance, race or ethnicity, relationship status, and sex or gender (5–7). For example, in 2025, cost-related barriers to accessing health care were reported more often by Hispanic than non-Hispanic people, lower-income than higher-income individuals, and uninsured than insured individuals (2). The wide range of costs and economic burdens that patients and caregivers face can heighten different PCBEOs across populations. Although existing research identifies differences in costs and burdens related to patient and caregiver characteristics (5–7), there is a lack of knowledge around how different populations experience various PCBEOs. Understanding how different types of PCBEOs vary across these populations is essential for informing interventions and health policies to best mitigate these burdens.

This scoping review addresses critical gaps in the literature by synthesizing evidence on differences in PCBEOs by sociodemographic characteristics and SDoH and by stratifying findings by category of PCBEO. This review also examines the instruments and data sources used to measure these differences and summarizes proposed strategies to mitigate differences in PCBEOs. Specifically, this review explores the following research questions:

1. What issues do researchers raise in the literature regarding differences in PCBEOs based on patient and/or family or caregiver sociodemographic characteristics or SDoH?
2. What data sources or instruments do researchers use to identify PCBEOs and examine differences in PCBEOs by sociodemographic characteristics or SDoH?
3. What strategies have researchers proposed to reduce PCBEOs for patients and/or families or caregivers whose sociodemographic characteristics or SDoH are associated with greater impacts of PCBEOs?

## 2 Materials and methods

This scoping review of the literature was guided by the preferred reporting items for systematic reviews and meta-analysis (PRISMA) and Joanna Briggs Institute guidance for scoping reviews (8, 9).

### 2.1 Search strategy and data sources

We conducted a comprehensive search of PubMed, CINAHL, EconLit, and Web of Science to identify original, peer-reviewed research published in English between January 1, 2015, and January 10, 2025. The search strategy included patient and caregiver terms, terms that indicate differences in sociodemographic characteristics or SDoH, and terms related to PCBEOs. Supplementary material A details the full search strategy.

### 2.2 Study inclusion criteria

#### 2.2.1 Population

Included studies focused on adult patients (aged 18 or older) and/or their families or caregivers in the United States who experienced physical, emotional, financial, or time-related burdens (i.e., PCBEOs) due to a medical condition. We classified burdens as related to a medical condition when a study specified a type of condition or patient population (e.g., older adult or end-of-life patients). We excluded studies that centered on health care providers or payers, involved pediatric populations (younger than 18 years), or were conducted outside the United States.

#### 2.2.2 Interventions

Eligible interventions encompassed (1) medical conditions contributing to PCBEOs among patients and/or their families or caregivers, (2) strategies aimed at addressing differences in PCBEOs, and (3) methods or instruments used to measure differences in PCBEOs. We excluded studies where burdens or differences were not attributable to a medical condition, such as those caused by natural disasters, or where burdens were unrelated to patients or their families or caregivers, such as health system operational challenges.

#### 2.2.3 Outcomes

Outcomes of interest included (1) differences in patient and/or family or caregiver burdens stratified by sociodemographic factors or SDoH, (2) effectiveness or impact of interventions designed to mitigate differences in PCBEOs, and (3) evaluations of tools or data sources used to assess differences in PCBEOs. We excluded studies that did not stratify burdens by sociodemographic factors or SDoH, lacked relevance to a medical condition and older adult or end-of-life populations, or did not address PCBEOs (e.g., studies focused solely on health care utilization, clinical outcomes, or system-level costs).

### 2.3 Study selection

We used DistillerSR software to conduct the review (10). For the title and abstract screening stage, two reviewers independently assessed each article (SD, SK, ES). A single reviewer conducted full-text reviews, with a third reviewer resolving any disagreements from the initial screening and performing quality checks on inclusion and exclusion decisions. Supplementary material B outlines the study selection criteria.

### 2.4 Data abstraction and synthesis

One reviewer abstracted key study characteristics and outcomes, and a second reviewer verified the data for accuracy and completeness (SD, SK, ES). We synthesized findings using narrative summaries and tables. Following recommended scoping review methods, we conducted an inductive thematic analysis, charting and comparing extracted data to identify recurring patterns across studies. Consistent with scoping review guidance by Peters et al. (2015), we organized themes into conceptual categories aligned with the review objectives (9). As per scoping review guidelines, we did not conduct risk-of-bias assessments, quantitative synthesis, or evaluations of evidence strength (11).

## 3 Results

### 3.1 Study selection and characteristics

**Figure 2:**
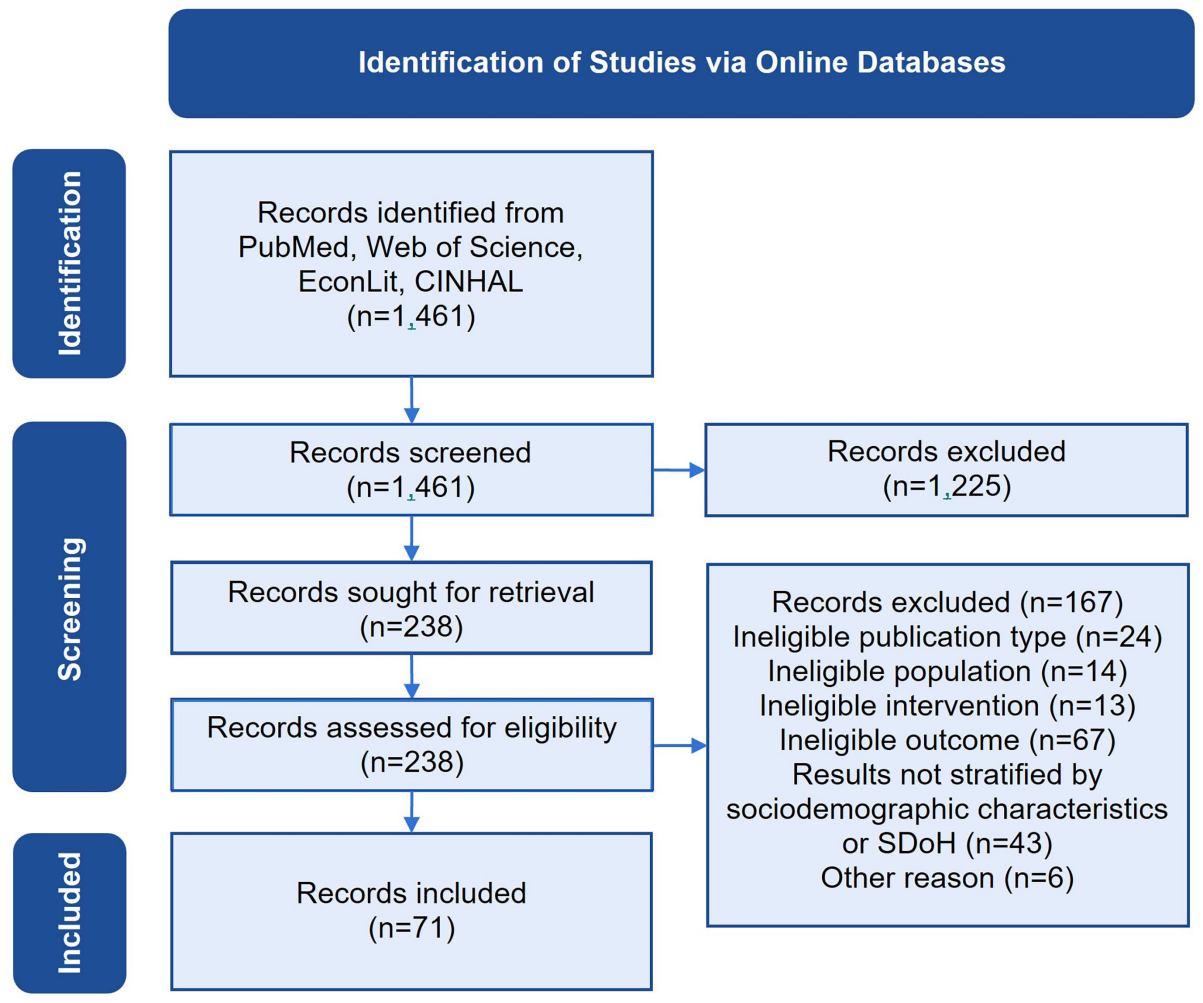
Differences in patient-centered burdens and economic outcomes: PRISMA diagram.

The database search yielded 1,461 titles and abstracts; after two stages of screening, we included a total of 71 studies (Figure 2).

Of the 71 included studies, 51 focused on patients, 14 on families or caregivers, and 6 included both groups. Cancer was the most frequently examined medical condition, representing more than half of studies (*n* = 41). Most cancer-focused studies included participants with any cancer type (*n* = 21), although some examined specific cancers such as breast (*n* = 6), colorectal (*n* = 5), and head and neck cancers (*n* = 4). Additional studies centered on specific populations or conditions, including older adult populations (*n* = 5), people with dementia (*n* = 5), and patients at the end of life (*n* = 3). Most studies employed cross-sectional designs (*n* = 64), with fewer retrospective cohort studies (*n* = 5) and qualitative studies (*n* = 3). In terms of data sources, 37 studies conducted secondary analyses of existing survey data, whereas 29 collected primary quantitative data through surveys. Additional sources included qualitative interviews (*n* = 5), medical claims data (*n* = 2), and electronic health records (*n* = 1). Geographically, 34 studies examined PCBEO differences at the national level. Seventeen were conducted within a single institution or site, 11 spanned multiple sites within a single state, 7 covered multiple states, and 2 had unclear or unstated geographic coverage. Supplementary material C provides detailed information for each included study.

### 3.2 Synthesized findings

#### 3.2.1 RQ1: What issues do researchers raise in the literature regarding differences in PCBEOs based on patient and/or family or caregiver sociodemographic characteristics or SDoH?

##### 3.2.1.1 Categories and frequencies of PCBEOs identified in the literature

Our review examined four domains of PCBEOs: direct medical costs, direct non-medical costs, indirect impacts, and intangible burdens (Table 1). Intangible burdens were the most frequently reported PCBEO category (*n* = 55), followed by direct medical costs (*n* = 27) and indirect impacts (*n* = 17), whereas direct non-medical costs were least commonly reported (*n* = 9). Across all domains, three subcategories emerged as the most frequently studied: financial toxicity (i.e., stress and anxiety caused by OOP costs, financial debt, or lost income) (*n* = 23), delaying or forgoing medical treatment due to cost (n = 19), and medical financial debt (*n* = 17).

**Table 1:** Categories of PCBEOs for patients and/or families or caregivers examined among included studies (*N* = 71).

Category of PCBEOs	Studies (*N* = 71)		
	Patients only (*n* = 51)	Caregivers only (*n* = 14)	Patients & caregivers (*n* = 6)
Direct medical costs (*n* = 27)			
Medical financial debt (*n* = 17)	Patients (*n* = 16)	Caregivers (*n* = 0)	Patients & caregivers (*n* = 1)
	(12–17, 19–28)	None	(18)
Out-of-pocket medical costs (*n* = 11)	Patients (*n* = 10)	Caregivers (*n* = 0)	Patients & caregivers (*n* = 1)
	(22, 29–34, 36–38)	None	(35)
Direct non-medical costs (*n* = 9)			
Filed for bankruptcy (*n* = 6)	Patients (*n* = 6)	Caregivers (*n* = 0)	Patients & caregivers (*n* = 0)
	(12, 15, 19, 20, 25, 26)	None	None
Other direct non-medical costs (*n* = 3)	Patients (*n* = 1)	Caregivers (*n* = 2)	Patients & caregivers (*n* = 0)
	(40)	(39, 41)	None
Indirect impacts (*n* = 17)			
Time spent providing unpaid caregiving (*n* = 8)	Patients (*n* = 1)	Caregivers (*n* = 7)	Patients & caregivers (*n* = 0)
	(30)	(39, 41–46)	None
Time spent traveling to or between medical appointments (*n* = 2)	Patients (*n* = 2)	Caregivers (*n* = 0)	Patients & caregivers (*n* = 0)
	(48, 49)	None	None
Time to attend medical appointments (*n* = 1)	Patients (*n* = 0)	Caregivers (*n* = 1)	Patients & caregivers (*n* = 0)
	None	(44)	None
Unpaid time off work, early retirement (*n* = 7)	Patients (*n* = 5)	Caregivers (*n* = 2)	Patients & caregivers (*n* = 0)
	(16, 17, 21, 25, 48)	(46, 47)	None
Other indirect impacts (*n* = 3)	Patients (*n* = 0)	Caregivers (*n* = 2)	Patients & caregivers (*n* = 1)
	None	(47, 50)	(51)
Intangible burdens (*n* = 55)			
Changes in employment status or insurance coverage (*n* = 3)	Patients (*n* = 3)	Caregivers (*n* = 0)	Patients & caregivers (*n* = 0)
	(17, 25, 48)	None	None
Delaying or forgoing medical treatments due to cost (*n* = 19)	Patients (*n* = 19)	Caregivers (*n* = 0)	Patients & caregivers (*n* = 0)
	(12, 14, 21–24, 26, 28, 48, 64–73)	None	None
Diminished household wealth (*n* = 4)	Patients (*n* = 4)	Caregivers (*n* = 0)	Patients & caregivers (*n* = 0)
	(12, 21, 28, 74)	None	None
Family/social life impacts (*n* = 5)	Patients (*n* = 1)	Caregivers (*n* = 3)	Patients & caregivers (*n* = 1)
	(16)	(39, 46, 52)	(82)
Fear and anxiety about future ability to afford medical treatments (*n* = 6)	Patients (*n* = 5)	Caregivers (*n* = 1)	Patients & caregivers (*n* = 0)
	(19, 25, 70, 74, 75)	(76)	None
Food insecurity, housing insecurity (*n* = 6)	Patients (*n* = 5)	Caregivers (*n* = 0)	Patients & caregivers (*n* = 1)
	(12, 13, 16, 25, 26)	None	(51)
Hindered career or education advancement (*n* = 3)	Patients (*n* = 2)	Caregivers (*n* = 1)	Patients & caregivers (*n* = 0)
	(12, 36)	(76)	None
Quality-of-life impacts (not quality-adjusted life years or disability-adjusted life years) (*n* = 4)	Patients (*n* = 1)	Caregivers (*n* = 3)	Patients & caregivers (*n* = 0)
	(81)	(39, 43, 76)	None
Reduced spending on basic goods, groceries, and leisure activities (*n* = 10)	Patients (*n* = 9)	Caregivers (*n* = 0)	Patients & caregivers (*n* = 1)
	(12, 13, 15–17, 20, 25, 26, 28)	None	(51)
Financial toxicity [i.e., stress and anxiety directly caused by out-of-pocket costs, financial debt, or lost income (*n* = 23)]	Patients (*n* = 16)	Caregivers (*n* = 5)	Patients & caregivers (*n* = 2)
	(14–17, 23–25, 38, 53, 54, 56–59, 62, 63)	(42, 46, 52, 60, 61)	(51, 55)
Other intangible burdens (*n* = 9)	Patients (*n* = 7)	Caregivers (*n* = 1)	Patients & caregivers (*n* = 1)
	(28, 40, 59, 65, 73, 79, 80)	(78)	(77)

Table 1 summarizes the specific types of PCBEOs reported within each domain. Twenty-seven studies reported direct medical costs, defined as monetary expenses paid by patients and/or their families or caregivers for medical care. Among these, 17 studies examined medical financial debt (12–28), and 11 studies reported OOP medical costs (22, 29–38). Most studies in this category focused exclusively on patients (*n* = 25), whereas two included both patients and caregivers (18, 35).

Nine studies addressed direct non-medical costs, defined as expenses incurred by patients and/or families or caregivers to access medical care, even though those costs are not part of the treatment itself (Table 1). Six studies reported bankruptcy filings (12, 15, 19, 20, 25, 26), and three studies described other direct non-medical costs such as the financial impact on caregivers (39–41). Of these, seven studies focused on patients (12, 15, 19, 20, 25, 26, 40), and two focused on caregivers (39, 41).

Seventeen studies described indirect impacts, defined as tangible burdens that patients, families, or caregivers experience without involving direct spending (Table 1). The most frequently reported subcategory was unpaid caregiving time (*n* = 8) (30, 39, 41–46), followed by unpaid time off work or early retirement (*n* = 7) (16, 17, 21, 25, 46–48). Other reported impacts included travel time to or between medical appointments (*n* = 2) (48, 49), time spent attending appointments (*n* = 1) (44), and other indirect impacts (*n* = 3) such as presenteeism (47, 50, 51). Seven studies focused on patients (16, 17, 21, 25, 30, 48, 49), nine on caregivers (39, 41–46, 50, 52), and one included both groups (51).

Intangible burdens, which include non-monetizable impacts on patients and caregivers, were the most frequently reported PCBEOs, appearing in 55 studies (Table 1). The most common subcategory was financial toxicity (*n* = 23) (14–17, 23–25, 38, 42, 46, 51–63). Other frequently reported burdens included delaying or forgoing medical treatment due to cost (*n* = 19) (12, 14, 21–24, 26, 28, 48, 64–73); reduced spending on basic goods, groceries, and leisure activities (*n* = 10) (12, 13, 15–17, 20, 25, 26, 28, 51); and fear or anxiety about future affordability of care (*n* = 6) (19, 25, 70, 74–76). Food or housing insecurity was reported in six studies (12, 13, 16, 25, 26, 51). Other intangible burdens were reported in 9 studies (28, 40, 59, 65, 73, 77–80), including composite caregiver burden (78) and difficulty paying non-medical bills (80).

##### 3.2.1.2 Differences in PCBEOs by sociodemographic characteristics and SDoH

We examined differences in PCBEOs across sociodemographic characteristics and SDoH categories that appeared in three or more studies. Nine characteristics met this threshold and reported statistically significant differences (*p* < 0.05): age, education, employment status, geographic location, income, insurance coverage, race or ethnicity, relationship status, and sex or gender (Table 2). Table 2 lists the studies that found significant differences in PCBEOs across these characteristics and the four PCBEO domains. Our primary analysis focused on characteristics with three or more significant findings, but all statistically significant results are detailed in the abstraction tables in Supplementary material C.

**Table 2:** Overview of statistically significant differences in PCBEOs, by patient and/or family or caregiver sociodemographic characteristics and SDoH (*N* = 70).

Sociodemographic characteristic	Studies (*N* = 70)			
	Direct medical	Direct non-medical	Indirect impacts	Intangible burdens
Age (*n* = 27)	Direct medical (*n* = 11)	Direct non-medical (*n* = 5)	Indirect (*n* = 4)	Intangible (*n* = 21)
	(14, 19–21, 25, 26, 28, 29, 31, 34, 35)	(19, 20, 25, 26, 40)	(21, 25, 47, 49)	(14, 19–21, 25, 26, 28, 40, 53, 58, 59, 62, 63, 65, 69–71, 74, 75, 77, 79)
Education (*n* = 12)	Direct medical (*n* = 4)	Direct non-medical (*n* = 0)	Indirect (*n* = 4)	Intangible (*n* = 10)
	(14, 21, 24, 34)	None	(21, 43, 44, 46)	(14, 21, 24, 43, 46, 53, 58, 63, 65, 75)
Employment status (*n* = 7)	Direct medical (*n* = 2)	Direct non-medical (*n* = 1)	Indirect (*n* = 2)	Intangible (*n* = 7)
	(19, 21)	(19)	(21, 48)	(19, 21, 48, 54, 63, 70, 74)
Geographic location (*n* = 14)	Direct medical (*n* = 8)	Direct non-medical (*n* = 2)	Indirect (*n* = 3)	Intangible (*n* = 7)
	(12, 20, 22, 28, 29, 33–35)	(12, 20)	(44, 49, 50)	(12, 20, 22, 28, 55, 64, 70)
Income (*n* = 32)	Direct medical (*n* = 9)	Direct non-medical (*n* = 2)	Indirect (*n* = 6)	Intangible (*n* = 24)
	(14, 21, 22, 25, 27, 31, 33–35)	(25, 39)	(21, 25, 39, 44, 45, 47)	(14, 21, 22, 25, 39, 54, 56–60, 62, 63, 65–67, 69–71, 73, 75, 79–81)
Insurance coverage (*n* = 27)	Direct medical (*n* = 14)	Direct non-medical (*n* = 1)	Indirect (*n* = 1)	Intangible (*n* = 19)
	(13, 14, 16, 19, 21, 23, 27, 31–35, 37, 38)	(19)	(44)	(13, 14, 16, 19, 21, 23, 38, 48, 51, 58, 63, 66, 68–71, 75, 80, 81)
Race or ethnicity (*n* = 29)	Direct medical (*n* = 12)	Direct non-medical (*n* = 4)	Indirect (*n* = 8)	Intangible (*n* = 23)
	(14, 17, 19, 21, 24, 26, 29, 30, 34–36, 38)	(19, 26, 39, 40)	(17, 21, 30, 39, 42–44, 49)	(14, 17, 19, 21, 24, 26, 36, 38–40, 42, 43, 52, 53, 56, 58, 60, 63, 66, 67, 76, 78, 82)
Relationship status (*n* = 11)	Direct medical (*n* = 6)	Direct non-medical (*n* = 1)	Indirect (*n* = 2)	Intangible (*n* = 11)
	(13, 14, 19–21, 28)	(20)	(21, 43)	(13, 14, 19–21, 28, 43, 65, 69, 71, 72)
Sex or gender (*n* = 21)	Direct medical (*n* = 8)	Direct non-medical (*n* = 2)	Indirect (*n* = 5)	Intangible (*n* = 15)
	(14, 18, 21, 24, 31, 34–36)	(39, 41)	(21, 39, 41, 43, 49)	(14, 21, 24, 36, 39, 43, 52, 58, 61, 62, 65, 69–71, 77)

Table 2 presents sociodemographic characteristics or SDoH categories with three or more studies that reported statistically significant differences in PCBEOs. As a result, the frequency counts in this table do not match those in the other tables or the total number of articles included in the review.

Twenty-seven studies reported differences in PCBEOs by age (Table 2). Intangible burdens were the most frequently examined cost category (*n* = 21), followed by direct medical costs (*n* = 11), direct non-medical costs (*n* = 5), and indirect impacts (*n* = 4). Findings by age were mixed. Eighteen studies found that younger adults experienced higher burdens (14, 19–21, 25, 26, 28, 31, 40, 47, 53, 59, 62, 63, 69, 71, 74, 77), whereas nine studies reported greater burdens among older adults (29, 34, 35, 49, 58, 65, 70, 75, 79). Five studies reported greater intangible burdens among older adults, including delayed medical treatment (65), cost-saving actions (70), difficulty paying medical bills (75), and financial toxicity (58, 79). However, 14 studies reported higher intangible burdens among younger individuals, including greater financial toxicity (25, 28, 53, 59, 62, 63), caregiving burden (40, 77), reduced spending on essentials (20, 26), food or housing insecurity (26), borrowing money (69, 71), and delaying or forgoing treatment due to cost (21, 26, 69, 71). All four studies examining direct non-medical costs found higher burdens among younger adults (19, 25, 26, 40). Two studies reported indirect impacts: one found greater work absenteeism among younger adults (47), and one (49) found older adults spent more time traveling to appointments. Among studies on direct medical costs, three found higher OOP expenses among older adults (29, 34, 35), whereas one found higher OOP costs among younger adults (31). Two studies found associations between younger age and greater medical financial debt (14, 19).

Twelve studies examined PCBEOs by educational attainment (Table 2). Most (*n* = 10) found that individuals with lower education levels experienced greater burdens. Intangible burdens were most frequently reported (*n* = 10), followed by indirect impacts (*n* = 4) and direct medical costs (*n* = 4). No studies reported significant findings for direct non-medical costs. Lower education was associated with financial toxicity (53, 58, 63), caregiving burden (43, 46), and delays in care due to cost (14, 21, 65). Three studies found that lower education was linked to increased unpaid caregiving time (43, 44, 46). Among studies on direct medical costs, three reported higher financial debt among those with lower education (14, 21, 24), whereas one found higher OOP expenditures among those with higher education (34).

Seven studies examined PCBEOs by employment status (Table 2), primarily focusing on intangible burdens. Three studies assessed direct medical costs, direct non-medical costs, and indirect impacts (19, 21, 48). Four studies found that unemployed individuals experienced greater burdens, including higher odds of bankruptcy (19), financial hardship (19), financial toxicity (54), delayed or forgone care (21), and reduced cost-saving behaviors (70). However, two studies reported higher burdens among employed individuals. One study attributed this to loss of public insurance and work-related time constraints (48), whereas another (74) did not distinguish unemployment from retirement, suggesting that unemployment may reflect financial stability in some cases.

Fourteen studies examined PCBEOs by geographic location (Table 2). Direct medical costs were most frequently reported (*n* = 8), followed by intangible burdens (*n* = 7), indirect impacts (*n* = 3), and direct non-medical costs (*n* = 2). Comparisons included rural versus urban (*n* = 7), regional differences (*n* = 5), and Area Deprivation Index (ADI) analyses (*n* = 2). Among rural versus urban comparisons, five studies found greater burdens in rural areas, including financial hardship (20, 22, 55), caregiving time (44), and reduced support (12). Two studies reported less favorable outcomes in urban areas, including missed work (50) and financial hardship (12). Regional comparisons showed higher burdens in the South, including OOP costs (33), financial strain (35), and forgone care (64). Two studies found less favorable outcomes in the South, Midwest, and West compared with the Northeast (34, 70). Both ADI-based studies reported greater burdens in more deprived areas (28, 49).

Thirty-two studies examined PCBEOs by income level (Table 2). Intangible burdens were most frequently reported (*n* = 24), followed by direct medical costs (*n* = 9), indirect impacts (*n* = 6), and direct non-medical costs (*n* = 2). Most studies (*n* = 29) found that lower income was associated with higher burdens. Three studies reported greater burdens among higher-income individuals, including inability to pay medical bills (80) and higher OOP costs (31, 34). Among studies linking lower income to higher intangible burdens, financial toxicity was most common (*n* = 10) (25, 54, 57–60, 63, 73, 75, 79), followed by delayed care (*n* = 4) (65, 66, 69, 71), financial hardship (*n* = 4) (21, 22, 56, 62), inability to pay for care (*n* = 3) (14, 67, 80), cost-saving actions (*n* = 1) (70), caregiver burden (*n* = 1) (39), and work absenteeism (*n* = 1) (81).

Insurance coverage was associated with PCBEOs in 27 studies (Table 2), most commonly in relation to intangible burdens (*n* = 19) and direct medical costs (*n* = 15), followed by indirect impacts (*n* = 1) and direct non-medical costs (*n* = 1). Two studies found that high-deductible plans were linked to greater hardship (23, 37), and one study reported that comprehensive coverage (e.g., military, Medicare) was associated with less financial stress (51). Findings on intangible burdens were mixed. Greater intangible burdens were reported among individuals with private insurance in three studies (14, 63, 68), public insurance in six studies (13, 21, 38, 58, 66, 81), and no insurance in three studies (70, 71, 75). Studies on direct medical costs primarily addressed OOP medical expenses. Uninsured patients faced higher OOP medical costs in five studies (19, 31, 32, 35, 38). One study found that public insurance was associated with a higher percentage of income spent on OOP medical costs compared with private insurance (33), whereas three studies found higher costs for private insurance (34, 35, 48). Only one study reported on direct non-medical costs, finding that public insurance was associated with greater bankruptcy risk than private insurance (19). One study on indirect impacts found that public insurance was associated with increased unpaid caregiving time compared with private insurance (44).

Twenty-nine studies examined differences in PCBEOs by race or ethnicity (Table 2). Intangible burdens were the most frequently reported (*n* = 23), followed by direct medical costs (*n* = 12), indirect impacts (*n* = 8), and direct non-medical costs (*n* = 4). Although findings varied across racial and ethnic groups, several consistent patterns emerged. Studies examining caregiver burden (*n* = 9) (39, 40, 42–44, 52, 60, 76, 82) found that Black or Hispanic caregivers experienced greater PCBEOs than non-Hispanic White caregivers. These PCBEOs included increased time spent providing unpaid care (39, 42–44), impacts on family and social life (39, 52, 82), non-monetary quality-of-life burdens (39, 43, 76), and financial toxicity (42, 52, 60). In contrast, findings for patients were less consistent. Eight studies reported higher PCBEOs among Black or Hispanic patients compared with non-Hispanic White patients (14, 17, 19, 21, 26, 53, 56, 63), whereas five studies found greater burdens among non-Hispanic White patients (34–36, 58, 67).

Relationship status was associated with PCBEOs in 11 studies (Table 2), with all studies reporting differences in intangible burdens. Six studies also examined direct medical costs, two explored indirect impacts, and one addressed direct non-medical costs. Most studies (n = 8) found that unmarried patients and caregivers experienced greater intangible burdens than their married counterparts. These intangible burdens included delaying or forgoing care due to cost (21, 69, 71, 72), skipping medications (65), borrowing money (19), and experiencing financial toxicity (20, 28). One study (20) also found that unmarried individuals were more likely to report bankruptcy (direct non-medical) compared with married individuals. However, two studies reported greater burdens among married versus unmarried individuals. One study (14) found that married adults were more likely than unmarried adults to worry about paying large cancer-related medical bills, and another study (43) found that caregivers who had never married reported lower overall caregiving burden than married or partnered caregivers. Additionally, one study (13) noted that cancer survivors who struggled to afford basic living expenses but avoided medical debt were more often unmarried, whereas those who incurred medical debt but could meet basic needs were more often married.

Twenty-one studies reported differences in PCBEOs by sex or gender (Table 2). Intangible burdens were the most examined (n = 15), followed by direct medical costs (n = 8), indirect impacts (n = 5), and direct non-medical costs (n = 2). All five studies examining caregiver or family burden, whether as composite intangible burden or indirect caregiving time, found that female caregivers experienced greater burdens than male caregivers (39, 43, 52, 71, 77). Among studies focused on financial stress or hardship, four reported higher burdens for male patients and caregivers (41, 58, 61, 62), whereas three found greater burdens for female patients (21, 24, 35). All three studies investigating delays in care or skipped medications (intangible) found that female patients were more likely to experience these burdens (14, 65, 69). Two studies reported higher OOP medical expenses for female patients (31, 34), whereas one study found that families with male cancer survivors were more likely to incur medical debt (direct medical) than those with female survivors (18). Additionally, one study reported longer travel times to medical appointments for male patients than for female patients (39).

#### 3.2.2 RQ2: What data sources or instruments do researchers use to identify PCBEOs and examine differences in PCBEOs by sociodemographic characteristics or SDoH?

Across the 71 included studies, we identified 37 distinct instruments or data sources used to measure differences in PCBEOs (Table 3). The most frequently used source was self-designed questionnaires (*n* = 24), followed by the National Health Interview Survey (NHIS) (*n* = 12) and the Medical Expenditure Panel Survey (MEPS) (*n* = 9). These instruments varied in scope, design, and validation status.

**Table 3:** Sources of data or instruments for measuring differences in PCBEOs (*N* = 71).

Sources of data or instruments	Validated	Publicly available	References (*N* = 71)
Primary data collection or analysis			
Self-designed questionnaire (*n* = 24)	No	No	(12, 14–17, 20, 21, 26, 28, 29, 39, 47, 52, 57, 59–62, 71, 74, 76, 78, 81, 82)
Comprehensive score for financial toxicity (COST) (*n* = 6)	Yes	No (permission from developer)	(25, 28, 54, 55, 59, 79)
Qualitative interviews (*n* = 5)	No	No	(13, 16, 48, 51, 55)
Zarit caregiver burden scale (*n* = 5)	Yes	Partial (may require permission/license)	(45, 76–78, 82)
Economic strain and resilience in cancer (ENRICh) (*n* = 3)	Yes	No	(16, 53, 63)
Caregiver reaction assessment scale (CRAS) (*n* = 2)	Yes	Partial (contact developer)	(39, 40)
Area deprivation index (*n* = 1)	Yes	Yes	Bai et al. (49)
Bakas caregiving outcomes scale (*n* = 1)	Yes	Partial (permission required)	Jessup et al. (52)
Braden scale, Missouri alliance for home care (MAHC-10) (*n* = 1)	Yes	Yes	Hallinan et al. (77)
Caregiver quality of life index-revised (CQLI-R) (*n* = 1)	Yes	Partial (requires permission)	Starr et al. (76)
Caregiver-centered communication questionnaire (CCCQ) (*n* = 1)	Yes	Partial (contact authors)	Starr et al. (76)
Financial management behavior scale (FMBS) (*n* = 1)	Yes	Yes	Patel et al. (58)
Generalized anxiety disorder 7-item (GAD-7) scale (*n* = 1)	Yes	Yes	Starr et al. (76)
Geriatric depression scale-short form (GDS-S) (*n* = 1)	Yes	Yes	Hallinan et al. (77)
Modified caregiver strain index (MCSI) (*n* = 1)	Yes	Yes	Pereira-Osorio et al. (42)
Oberst caregiving burden scale (*n* = 1)	Yes	No	Jessup et al. (52)
Patient health questionnaire 9-item (PHQ-9) (*n* = 1)	Yes	Yes	Starr et al. (76)
PROMIS short forms v2.0 (*n* = 1)	Yes	Yes	McDougall et al. (81)
Saint louis university mental status exam (SLUMS) (*n* = 1)	Yes	Yes	Hallinan et al. (77)
Work productivity and activity impairment questionnaire (*n* = 1)	Yes	Partial (requires license for commercial use)	Ganapathy et al. (47)
Secondary data analysis			
National health interview survey (NHIS) (*n* = 12)	Yes	Yes	(23, 24, 27, 66–70, 72, 73, 75, 80)
Medical expenditure panel survey (MEPS) (*n* = 9)	Yes	Yes	(15, 17, 20, 31–35, 56)
National study of caregiving (NSOC) (*n* = 2)	Yes	Yes	(46, 50)
Behavioral risk factor surveillance system (*n* = 2)	Yes	Yes	(12, 64)
Electronic health records (*n* = 2)	N/A	No	(49, 56)
Health and retirement study (*n* = 2)	Yes	Yes	(30, 36)
National cancer institute’s health information national trends survey (HINTS) (*n* = 2)	Yes	Yes	(22, 62)
National health and aging trends study (*n* = 2)	Yes	Yes	(43, 50)
Caregiving in the United States 2015 survey (*n* = 1)	No	Partial (report available)	Lee et al. (41)
Healthcare access and utilization survey within the *all of us* database (*n* = 1)	Yes	Yes	Boddu et al. (65)
Kaiser family foundation health insurance study (*n* = 1)	Yes	Yes	Lee et al. (59)
LIVESTRONG survey (*n* = 1)	Yes	Yes	Banegas et al. (19)
Medical claims data (*n* = 1)	N/A	No	Berinstein et al. (37)
National inpatient sample (*n* = 1)	N/A	No	Ng et al. (38)
National survey of children with special health care needs (*n* = 1)	Yes	Yes	Miller et al. (44)
Panel study of income dynamics (*n* = 1)	Yes	Yes	Grafova et al. (18)
University of Alabama at Birmingham tumor registry (*n* = 1)	N/A	No	Davis et al. (56)

In addition to the 24 studies using self-designed questionnaires, we identified 19 studies that employed previously developed instruments for primary data collection. Of these, 17 were validated and 9 were publicly available. Seventeen studies relied on secondary data sources, 12 of which were validated and publicly accessible.

Among studies using self-designed questionnaires, all but two examined intangible burdens (12, 14–17, 20, 21, 26, 28, 39, 52, 57, 59–62, 71, 74, 76, 78, 81, 82). Similarly, nearly all studies using NHIS data (23, 24, 66–70, 72, 73, 75, 80) focused on intangible burdens. In contrast, studies using MEPS data (15, 17, 20, 31–35) primarily examined direct medical or direct non-medical costs, with five studies focusing exclusively on direct medical costs.

#### 3.2.3 RQ3: What strategies have researchers proposed to reduce PCBEOs for patients and/or families or caregivers whose sociodemographic characteristics or SDoH are associated with greater impacts of PCBEOs?

Fifteen studies identified strategies aimed at mitigating PCBEO disparities related to sociodemographic characteristics and SDoH (Table 4). A central theme was enhancing provider–patient communication about financial risk and treatment costs through provider training, shared decision-making, and documentation of cost-related discussions (14, 55–57, 62).

**Table 4:** Summary of proposed strategies for addressing differences in PCBEOs (*n* = 15).

Theme	References
Access to financial resources and navigation support (*n* = 5)	
Simplify and standardize access to essential financial resources and programs	Liang et al. (55)
Connect patients and/or families or caregivers to financial navigation programs	(14, 56, 57, 62)
Offer support services such as financial and insurance counseling, patient navigation, medication assistance programs, and social services	Vanderpool et al. (62)
Financial toxicity screening and communication (*n* = 4)	
Proactively and periodically screen for financial toxicity	(55, 56)
Improve training on cost communication among health care providers	Liang et al. (55)
Use thresholds based on sociodemographic characteristics to identify patients for earlier and more intensive interventions for financial toxicity	Liang et al. (25)
Increase financial risk and cost-of-care conversations between patients and their health care providers	Vanderpool et al. (62)
Limit patient cost sharing in health insurance plans	
Change the way care is delivered and financed through Medicaid and other health insurance reforms	Gu et al. (64)
Expand insurance coverage	Boddu et al. (65)
Develop a plan quality and network breadth metric to increase transparency in health insurance plans	McKenna et al. (68)
Simplify administrative processes to increase provider participation in insurance plans	McKenna et al. (68)
Target state-level policy to address provider participation in health insurance plans	McKenna et al. (68)
Community and social support services (*n* = 3)	
Enhance community resources assisting with transportation to and from medical visits	Boddu et al. (65)
Launch programs to help patients understand employment protections	Berghuijs et al. (74)
Help caregivers access support from their social network of family members and friends through communication skills training and peer support	Washington et al. (40)
Tailor clinical interventions based on cost and insurance barriers (*n* = 2)	
Tailor prescriptions based on patients’ insurance status and cost barriers so they can get a prescription they can afford	Shah et al. (66)
Develop and adapt existing effective interventions to improve chronic disease self-management	McDougall et al. (81)
Documentation and data collection (*n* = 1)	
Document discussions between providers and patients regarding costs of care	Davis et al. (56)
Increase data collection and documentation regarding patient-reported financial hardship and its effect on patient outcomes	Davis et al. (56)
Geographic prioritization of resources (*n* = 1)	
Use the area deprivation index to prioritize resources to mitigate financial toxicity	Corkum et al. (28)

Several studies emphasized the need for health insurance reforms to improve transparency, reduce cost-sharing, and expand coverage (13, 64, 65, 68). One study recommended tailoring prescribed drug formulations based on insurance status to reduce OOP medical expenses (66).

Additional strategies included providing transportation support for medical appointments (65) and implementing financial navigation services equipped with screening tools for financial toxicity (25, 55, 56, 62). Some studies proposed targeted interventions using tools such as the ADI (28) and patient risk thresholds (25) to help prioritize resources for high-need populations.

Finally, several studies identified patient empowerment approaches as promising strategies. These included peer support programs (40), chronic illness self-management interventions (81), and education on employment protections (74), all aimed at reducing differences in PCBEOs.

## 4 Discussion

### 4.1 Summary of main findings

This scoping review provides a comprehensive synthesis of evidence on how PCBEOs differ across patient and/or family or caregiver populations based on sociodemographic characteristics and SDoH. We also summarized data sources, measurement instruments, and proposed mitigation strategies used to assess and address these differences. To our knowledge, this is the first scoping assessment of variations in PCBEOs based on sociodemographic and SDoH-related factors.

We included 71 studies spanning the four PCBEO domains defined by PCORI: direct medical costs, direct non-medical costs, indirect impacts, and intangible burdens. Intangible burdens emerged as the most frequently reported domain (*n* = 55), likely due to the widespread use of self-designed questionnaires, which more readily capture subjective financial concerns than structured data sources such as medical records or electronic health records (EHRs). The high proportion of studies focused on cancer patients and/or their families or caregivers may also contribute to this emphasis, given the well-documented long-term financial hardship associated with cancer care (19, 83).

Income (*n* = 32), race or ethnicity (*n* = 29), age (*n* = 27), and insurance coverage (*n* = 27) were the most frequently assessed sociodemographic characteristics or SDoH. Across studies, younger adults, individuals with lower educational attainment, residents of the Southern United States and rural areas, lower-income households, and Black or Hispanic populations were more often reported to experience greater indirect and intangible PCBEOs. In contrast, older patients and those with higher education or income levels more frequently reported higher OOP medical costs. These patterns may reflect underlying differences in access to and quality of care or financial resources across sociodemographic groups.

The consistently greater intangible burdens, despite lower direct medical costs, among populations with lower socioeconomic status suggest that lower direct costs may mask deeper financial strain rather than indicate lower overall need. Because financial hardship can limit the ability to spend on care, direct expenditure data alone likely underestimates the true burden of illness for these groups. Younger adults also consistently reported experiencing greater intangible and direct non-medical costs than older adults, potentially due to greater financial instability, more limited insurance coverage, or competing life demands that heighten the perceived impact of illness-related costs.

Differences in PCBEOs by race or ethnicity were also observed among patients and caregivers. Most of these studies focused on unpaid caregivers, potentially underrepresenting the financial implications of paid caregiving services. Findings related to employment status, relationship status, and patient sex or gender were more variable and did not reveal consistent patterns across studies.

Our findings build on prior work, showing that younger adults, people with lower household income, and Black or Hispanic populations report greater financial hardship. Evidence from a large population-based study of cancer survivors aligns with this pattern, identifying these groups as experiencing the highest levels of psychological and material financial strain (84). Broader research on financial-toxicity similarly emphasizes emotional and psychological consequences that often extend beyond measurable out-of-pocket costs (85, 86). Literature on SDoH reflects comparable patterns, documenting differences in both financial and non-financial impacts by income, education, geographic location, insurance status, and race or ethnicity (87, 88).

Our PCBEO domains closely align with the Innovation and Value Initiative and AcademyHealth Economic Impacts Framework, which captures direct medical costs, non-clinical healthcare costs, caregiver and family impacts, social impacts, and work- and education-related consequences (89). This alignment supports the conceptual foundation of our PCBEO categorization, while our explicit focus on intangible burdens adds nuance by highlighting emotional and relational impacts that are often less directly addressed by economic models.

Key strengths of this study include its broad literature base, which enabled synthesis across a wide range of populations, and the deliberate inclusion of caregivers, which adds depth by capturing the burdens experienced by family members and other unpaid caregivers. The methodological range, which incorporated quantitative and qualitative approaches, diverse sociodemographic and SDoH categories, and an expansive array of search terms, supports a robust and nuanced analysis. Presenting findings by PCBEO domain and demographic subgroup enhances practical relevance and offers clear, targeted insights for researchers and policymakers. Additionally, by compiling and organizing strategies proposed in the literature to reduce differences in PCBEOs, this review contributes to informed decision-making. Collectively, these strengths position our study as a valuable resource for future research and policy initiatives aimed at understanding and addressing the financial, emotional, physical, and time-related burdens faced by different patient and caregiver populations.

### 4.2 Limitations

This review has a few limitations. During article screening, we identified additional factors (e.g., immigration status) not captured in our original search strategy, which may have led to the omission of relevant studies and characteristics. However, we intentionally designed our search terms to encompass a broad range of sociodemographic characteristics and SDoH potentially associated with differences in PCBEOs. Rather than targeting specific population descriptors, we used inclusive terms such as “variation” and “difference” to identify studies addressing differential outcomes.

We also found that many sociodemographic categories, such as geographic location, income, and age, lacked standardized definitions across studies. This may have obscured important within-group variations, making it challenging to summarize findings across studies. For example, one study defined the youngest age category as 25–35 years (26), whereas others used broader ranges such as 18–55 years (21, 82). Analyzing sociodemographic factors independently may also overlook the intersecting burdens experienced by individuals belonging to multiple groups. To address these limitations, we synthesized findings thematically and emphasized overarching patterns and directional associations rather than absolute differences. Where appropriate, we applied broader classifications, such as older versus younger adults or higher versus lower income, to facilitate comparison.

To enhance relevance to the U.S. health care system, we restricted our review to studies published in English and conducted in the United States. As a result, our findings may have limited generalizability to PCBEOs experienced by patients, families, or caregivers in other countries.

This review did not include gray literature such as conference abstracts, technical reports, or white papers. Because scoping reviews aim to capture the full breadth of available evidence, excluding grey literature may have led to missed insights and contributed to publication bias. Future reviews could incorporate a structured grey-literature search to strengthen comprehensiveness.

Finally, we did not conduct a formal quality assessment of the studies included, because this was beyond the scope of the current review. As a result, we cannot draw conclusions about the overall strength of the evidence. However, future scoping reviews or meta-analyses focused on more specific topics—such as the intersectionality between PCBEOs—may help address questions related to the consistency and robustness of the evidence.

### 4.3 Implications for future research

Our review identified recurring associations between sociodemographic characteristics, SDoH, and PCBEOs, with younger adults, people with lower educational attainment, and lower-income households often experiencing greater burdens. Yet, the literature did not reveal a consistent overall pattern, either in the direction of associations or in their persistence across studies. This variability underscores the complex, context dependent relationships among sociodemographic factors, SDoH, and PCBEOs. These findings highlight the need for research that more comprehensively incorporates these variables and explicitly examines how they intersect to shape PCBEOs. Researchers assessing PCBEOs may consider systematically capturing sociodemographic characteristics and SDoH, and likewise, studies of SDoH may consider integrating PCBEOs. Doing so will support the development of a more cohesive evidence base to inform how these factors interact across contexts.

Studies that include sociodemographic characteristics or SDoH as confounding or descriptive variables may benefit from also measuring and reporting PCBEOs to enable subgroup analyses. Incorporating validated instruments, standardized sociodemographic categories, and varying data sources, such as claims data or EHR data, could improve comparability and generalizability. Expanding beyond cancer to include other prevalent conditions such as cardiovascular disease, diabetes, and mental health would help assess whether observed patterns are condition specific or broadly applicable. The overrepresentation of cancer studies may reflect search strategy limitations, funding priorities, or the visibility of financial toxicity in oncology.

Only a few studies used qualitative methods, limiting insight into the lived experiences of patients and caregivers. Interviews, focus groups, and participatory approaches could uncover burdens not captured by quantitative tools and help ensure that proposed strategies are grounded in real-world needs. Mixed findings regarding insurance coverage and intangible burdens such as financial toxicity warrant further investigation to clarify these associations. Direct non-medical costs, including transportation, lodging, and caregiving time, remain underexamined despite their potential impact on families. Developing standardized tools to measure indirect impacts and intangible burdens could help build a more complete picture of PCBEOs.

Finally, future studies should prioritize direct engagement with patients and caregivers. Their perspectives can reveal overlooked challenges and guide the design of policies and programs, such as financial navigation services, that are more responsive to their needs.

## 5 Conclusion

Patients and their families or caregivers experience a wide range of PCBEOs across multiple domains, including direct medical costs, non-medical expenses, indirect impacts, and intangible burdens. These burdens are not evenly distributed; rather, they vary substantially based on sociodemographic characteristics and SDoH. This scoping review captures the breadth and complexity of PCBEOs across diverse populations, underscoring that no group is entirely unaffected.

By categorizing PCBEOs and examining differences across population groups, this study offers a foundational understanding of how these burdens manifest and where differences are most evident. It also synthesizes strategies proposed in the literature to mitigate these differences, pointing to promising areas for future research.

Ongoing investigation into the nuanced and intersecting relationships between sociodemographic factors and PCBEOs is critical for developing effective interventions and informing future policies. As the health care system continues to evolve, addressing these differences must remain a priority to ensure that care is not only clinically effective but also financially and emotionally sustainable for patients and their families or caregivers.

## Data Availability

The original contributions presented in the study are included in the article/Supplementary material, further inquiries can be directed to the corresponding author.
