# Supplementary material for: Differences in patient-centered burdens and economic outcomes based on sociodemographic characteristics and social determinants of health: a scoping review
Source: Front Public Health. 2026 Jul 21;14:1784571. doi: 10.3389/fpubh.2026.1784571 (PMC13435972; doi:10.3389/fpubh.2026.1784571)
Supplement: Supplementary file 1 [file Supplementary_file_1.DOCX]

Supplementary Material

# Supplement A: Search Strategy

**Topic:** Socioeconomic and sociodemographic differences in PCBEOs

**Timeframe:** January 1, 2015–January 10, 2025

**Language:** English

**Geographic area:** United States

**Publication types:** Peer-reviewed literature

**Databases:** PubMed, CINAHL, EconLit, Web of Science

**Population Terms**

"adult*"[ti] OR "American*"[ti] OR "cancer survivor*"[ti] OR "caregiv*"[ti] OR "famil*"[ti] OR "female*"[ti] OR "individuals"[ti] OR "patient*"[ti] OR "people"[ti] OR "caregiv*"[ot] OR "famil*"[ot] OR "individuals"[ot] OR "nonbinary"[ot] OR "patient*"[ot] OR "people"[ot] OR "Caregivers"[Mesh] OR "Family"[Mesh:NoExp] OR "Patients"[Mesh:NoExp]

**AND**

**PCBEO Terms**

"socioeconomic disparit*"[ti] OR "socio-economic disparit*"[ti] OR "sociodemographic disparit*"[ti] OR "socio-demographic disparit*"[ti] OR "bankruptcy"[ti] OR "burden*"[ti] OR "caregiver burden*"[ti] OR "caregiving burden*"[ti] OR "caregiver distress"[ti] OR "coping strateg*"[ti] OR "delayed treatment"[ti] OR "early retirement"[ti] OR "economic distress"[ti] OR "emotional cost*"[ti] OR "economic impact*"[ti] OR "emotional distress"[ti] OR "emotional impact*"[ti] OR "financial cost*"[ti] OR "financial distress"[ti] OR "financial hardship*"[ti] OR "financial impact*"[ti] OR "financial strain"[ti] OR "financial toxicity"[ti] OR "medical debt*"[ti] OR "mental distress"[ti] OR "mental health distress"[ti] OR "mental health impact*"[ti] OR "missed work"[ti] OR "out-of-pocket"[ti] OR "paid leave"[ti] OR "paid time off"[ti] OR "patient burden*"[ti] OR "psychological distress"[ti] OR "psychological impact*"[ti] OR "reduced income"[ti] OR "social isolation"[ti] OR "time cost*"[ti] OR "treatment delay*"[ti] OR "treatment disruption*"[ti] OR "unpaid leave"[ti] OR "work loss"[ti] OR "socioeconomic disparit*"[ot] OR "socio-economic disparit*"[ot] OR "sociodemographic disparit*"[ot] OR "socio-demographic disparit*"[ot] OR "bankruptcy"[ot] OR "burden*"[ot] OR "caregiver burden*"[ot] OR "caregiving burden*"[ot] OR "caregiver distress"[ot] OR "coping strateg*"[ot] OR "delayed treatment"[ot] OR "early retirement"[ot] OR "economic distress"[ot] OR "emotional cost*"[ot] OR "economic impact*"[ot] OR "emotional distress"[ot] OR "emotional impact*"[ot] OR "financial cost*"[ot] OR "financial distress"[ot] OR "financial hardship*"[ot] OR "financial impact*"[ot] OR "financial strain"[ot] OR "financial toxicity"[ot] OR "medical debt*"[ot] OR "mental distress"[ot] OR "mental health distress"[ot] OR "mental health impact*"[ot] OR "missed work"[ot] OR "out-of-pocket"[ot] OR "paid leave"[ot] OR "paid time off"[ot] OR "patient burden*"[ot] OR "psychological distress"[ot] OR "psychological impact*"[ot] OR "reduced income"[ot] OR "social isolation"[ot] OR "time cost*"[ot] OR "treatment delay*"[ot] OR "treatment disruption*"[ot] OR "unpaid leave"[ot] OR "work loss"[ot] OR "Caregiver Burden"[Mesh] OR "Treatment Delay"[Mesh] OR "Psychological Distress"[Mesh:NoExp] OR "Financial Stress"[Mesh] OR "Social Isolation"[Mesh:NoExp]

**AND**

**Difference Terms**

"access"[ti] OR "demographic*"[ti] OR "difference*"[ti] OR "disadvantage*"[ti] OR "disparities"[ti] OR "disparity"[ti] OR "disproportion*"[ti] OR "excluded"[ti] OR "exclusion"[ti] OR "SDoH"[ti] OR "social determinants of health"[ti] OR "social determinant of health"[ti] OR "social divide"[ti] OR "sociodemographic*"[ti] OR "socio-demographic*"[ti] OR "socioeconomic*"[ti] OR "socio-economic*"[ti] OR "underrepresented"[ti] OR "under-represented"[ti] OR "underserved"[ti] OR "under-served"[ti] OR "unequal"[ti] OR "variation*"[ti] OR "access"[ot] OR "demographic*"[ot] OR "difference*"[ot] OR "disadvantage*"[ot] OR "disparities"[ot] OR "disparity"[ot] OR "disproportion*"[ot] OR "excluded"[ot] OR "exclusion"[ot] OR "inequalities"[ot] OR "inequality"[ot] OR "SDoH"[ot] OR "social determinants of health"[ot] OR "social determinant of health"[ot] OR "social divide"[ot] OR "sociodemographic*"[ot] OR "socio-demographic*"[ot] OR "socioeconomic*"[ot] OR "socio-economic*"[ot] OR "underrepresented"[ot] OR "under-represented"[ot] OR "underserved"[ot] OR "under-served"[ot] OR "unequal"[ot] OR "variation*"[ot] OR "Healthcare Disparities"[Mesh] OR "Health Status Disparities"[Mesh] OR "Socioeconomic Disparities in Health"[Mesh] OR "Health Equity"[Mesh] OR "Health Inequities"[Mesh:NoExp] OR "Social Determinants of Health"[Mesh] OR "Sociodemographic Factors"[Mesh] OR "Socioeconomic Factors"[Mesh:NoExp] OR "Health Disparate Minority and Vulnerable Populations"[Mesh:NoExp] OR "Vulnerable Populations"[Mesh]

**AND**

**United States Terms**

"United States"[Mesh] OR "United States"[tiab] OR "USA"[tiab] OR "U.S.A."[tiab] OR "U.S."[tiab] OR "United States"[Affiliation] OR "USA"[Affiliation] OR "U.S.A."[Affiliation] OR "U.S."[Affiliation] OR "US"[Affiliation]

**NOT**

**Exclusion Terms**

("Animals"[Mesh] NOT "Humans"[Mesh]) OR pmcbook OR pmcbooktitle OR pmcbookchapter OR "Comment"[Publication Type] OR "Letter"[Publication Type] OR "Editorial"[Publication Type] OR "adverse childhood experience*"[ti] OR "air pollution"[ti] OR "child abuse"[ti] OR "child neglect"[ti] OR "climate change"[ti] OR "elder abuse"[ti] OR "homicide*"[ti] OR "rat"[ti] OR "rats"[ti] OR "sexual abuse"[ti] OR "violence"[ti] OR "Australia*"[tiab] OR "Canada"[tiab] OR "Canadian*"[tiab] OR "China"[tiab] OR "German*"[tiab] OR "global burden"[ti] OR "low- and middle-income countr*"[tiab] OR "Sub-Saharan Africa"[tiab] OR "United Kingdom"[tiab]

Filters: English

# Supplement B: Selection Criteria

|  | **Inclusion** | **Exclusion** |
| --- | --- | --- |
| **Population** | 1) Adult (aged 18 or older) patients and/or their families or caregivers experiencing physical, emotional, financial, or time-related burdens caused by a medical condition | 1) Studies focusing on populations other than patients and/or their families or caregivers (e.g., providers, payers, health systems) 2) Studies focused on children (aged younger than 18) 3) Studies conducted outside the United States |
| **Intervention** | 1) Medical conditions causing physical, emotional, financial, or time-related burdens to patients and/or families or caregivers of patients 2) Strategies aimed at addressing differences in PCBEOs 3) Methods or instruments used to measure differences in PCBEOs | 1) Burdens or differences not caused by a medical condition (e.g., economic disruptions from the COVID-19 pandemic, natural disasters, job loss unrelated to health conditions) 2) Burdens unrelated to patients or their families or caregivers (e.g., provider or health systems-level challenges) |
| **Outcomes** | 1) Differences in patient and/or family/caregiver burdens stratified by sociodemographic factors or SDoH 2) Efficacy or impact of interventions designed to address differences in PCBEOs 3) Assessment of methods or instruments for evaluating differences in PCBEOs | 1) Studies that do not stratify burdens by sociodemographic factors or SDoH 2) Differences unrelated to physical, emotional, financial, or time-related burdens from medical conditions affecting patients or families or caregivers 3) Outcomes unrelated to medical conditions including violence or abuse 4) Outcomes that do not focus on economic factors or differences in PCBEOs (e.g., health care utilization, clinical outcomes, health systems-level costs) |
| **Setting and publication type** | 1) Studies conducted in the United States 2) Peer-reviewed articles published in 2015 or later 3) Publications available in English | 1) Non-peer-reviewed publications such as book sections, letters, editorials, or commentaries 2) Dissertations, working papers, news articles, or trade publications 3) Study protocols without results or findings 4) Publications in languages other than English 5) Published before 2015 |

# Supplement C: Abstraction Tables

| **Acquah, I., Valero-Elizondo, J., Javed, Z., Ibrahim, H. N., Patel, K. V., Ali, H. J. R., Menser, T., Khera, R., Cainzos-Achirica, M., & Nasir, K. (2021). Financial hardship among nonelderly adults with CKD in the United States. *American Journal of Kidney Diseases*, *78*(5), 658–668.** | | |
| --- | --- | --- |
| Study Characteristics | Study Purpose | To describe the scope and determinants of financial hardship among a nationally representative sample of adults with chronic kidney disease (CKD). |
|  | Source of Data | Secondary analysis of pre-existing survey data |
|  | Study Type | Cross-sectional |
|  | Data Collection Geography | National |
|  | Population | Patients |
|  | Sample Size | 1,425 patients |
|  | Medical Condition | CKD |
| Findings: Categories of PCBEOs | Categories of PCBEOs Examined | Direct medical costs |
|  | Direct Medical Costs | Other direct medical costs: Ability to pay bills |
| Findings: Differences in PCBEOs | Types of Differences Examined | Income, insurance |
|  | Income | Family income was significantly associated with inability to pay (*p*<0.001); 15.6% of middle/high income individuals reported inability to pay, and 26.4% of low-income individuals reported inability to pay. |
|  | Insurance | Insurance status was significantly associated with inability to pay (*p*<0.001); 15.1% of those with private health insurance, 19.7% of those on Medicaid, 12.6% of those with other insurance types, and 50.2% of uninsured individuals reported inability to pay. Only uninsured individuals were significantly more likely to not pay bills at all (*p*<0.001; odds ratio=5.67, 95% confidence interval [3.61, 8.92]). |
| Findings: Strategies for Addressing Differences | Strategies for Addressing Differences | No |
|  | Methods/Instruments | National Health Interview Survey |
| **Badger, T., Segrin, C., Crane, T., Morrill, K., & Sikorskii, A. (2024). Social determinants of health, psychological distress, and caregiver burden among informal cancer caregivers of cancer survivors during treatment. *Journal of Psychosocial Oncology*, *42*(3), 333–350.** | | |
| Study Characteristics | Study Purpose | To compare three ethnic/racial groups of informal cancer caregivers on social determinants of health and tested social determinants of health as predictors of psychological distress and caregiver burden. |
|  | Source of Data | Primary quantitative data collected through a survey |
|  | Study Type | Cross-sectional |
|  | Data Collection Geography | Multiple sites across multiple states |
|  | Population | Caregivers |
|  | Sample Size | 396 caregivers |
|  | Medical Condition | Cancer (all types) |
| Findings: Categories of PCBEOs | Categories of PCBEOs Examined | Direct non-medical costs  Indirect impacts  Intangible burdens |
|  | Direct Non-Medical Costs | Other direct non-medical costs: Caregiving impact on finances |
|  | Indirect Costs | Time spent providing unpaid caregiving |
|  | Intangible Burdens | Family/social life impacts, quality-of-life impacts (not quality-adjusted life-years or disability-adjusted life-years) |
| Findings: Differences in PCBEOs | Types of Differences Examined | Income, race/ethnicity, sex/gender |
|  | Income | When looking at specific social and health factors, having an income that barely meets or does not meet basic needs was a significant predictor of caregiver burden (β=-0.16, *p*=0.003). |
|  | Race/Ethnicity | Hispanic caregivers experienced higher mean burden across three caregiver burden indictors compared with non-Hispanic White and non-Hispanic caregivers of other races, namely impact on finances, impact on family, and impact on schedules.  Hispanic caregivers reported higher mean scores than other ethnicities on measures of impact on finances from caregiving (Hispanic: Mean=2.73, non-Hispanic White: 2.08, non-Hispanic other races: 2.35, *p*<0.001, effect size: .08), measures of impact on family (Hispanic: 2.22, non-Hispanic White: 1.88, non-Hispanic other races: 2.12 (*p*=0.001), and measures of impact on schedule: Hispanic: 2.73, non-Hispanic White: 2.46, non-Hispanic other races: 2.53 (*p*=.02). |
|  | Sex | Female caregivers reported greater average caregiver burden than non-female caregivers, after controlling for other factors in the model (β=0.11, *p*=0.022). |
| Findings: Strategies for Addressing Differences | Strategies for Addressing Differences | No |
|  | Methods/Instruments | Yes. Caregiver burden was assessed using the four subscales of the Caregiver Reaction Assessment Scale. |
| **Bai, J., Barandouzi, Z. A., Yeager, K. A., Graetz, I., Gong, C., Norman, M., Hankins, J., Paul, S., Torres, M. A., & Bruner, D. W. (2024). Analysis of travel burden and travel support among patients treated at a comprehensive cancer center in the Southeastern United States. *Supportive Care in Cancer*, *32*(7), 451.** | | |
| Study Characteristics | Study Purpose | To analyze the electronic medical record data of patients who received or were offered (thereafter referred to as “received”) travel support between 1/1/2021 and 5/1/2023.  Describe the travel burden and types and amount of travel support provided at a comprehensive cancer center in the Southeastern United States.  Examine factors associated with the level of travel burden and travel support received. |
|  | Source of Data | Medical records/electronic health records |
|  | Study Type | Cross-sectional |
|  | Data Collection Geography | Multiple sites in a single state |
|  | Population | Patients |
|  | Sample Size | 1,086 patients |
|  | Medical Condition | Cancer (all types) |
| Findings: Categories of PCBEOs | Categories of PCBEOs Examined | Indirect impacts |
|  | Indirect Costs | Time spent traveling to or between medical appointments |
| Findings: Differences in PCBEOs | Types of Differences Examined | Age, geographic location, race/ethnicity, sex/gender |
|  | Age | The longer the travel distance, the higher proportion of patients were in the age range 40–64 (48.1% of patients aged 40–64 had a travel distance ≥50 miles vs. 33.0% for 65 or older vs. 18.9% for 18–39, *p*<0.001). |
|  | Geographic Location | Patients residing in more disadvantaged neighborhoods had increased travel distance (correlation coefficient (r)=0.30, *p*<0.001, 95% confidence interval [CI] [0.24, 0.35]) and travel time (r=0.31, *p*<0.001, 95% CI [0.25, 0.36]) based on the Area Deprivation Index (ADI) nation rankings. |
|  | Race/Ethnicity | White patients who received travel support had longer travel distances (mean=72.8 miles for White vs. 51.2 miles for Black vs. 53.5 miles for other races, *p*<0.001) and higher travel times (mean=82.0 min. for White vs. 61.4 min. for Black vs. 65.4 min. for other races, *p*<0.001) than Black patients and other races, respectively.  Hispanic patients had shorter travel times than non-Hispanic patients (mean=45.3 min. vs. 67.9 min., *p*=0.043). |
|  | Sex | Male patients who received travel support had longer travel distances (mean=64.2 miles vs. 51.3 miles, *p*=0.005) and higher travel times (mean=71.8 min. vs. 61.9 min., *p*=0.004) than female patients, respectively. |
| Findings: Strategies for Addressing Differences | Strategies for Addressing Differences | No |
|  | Methods/Instruments | ADI |
| **Banegas, M. P., Guy, G. P., de Moor, J. S., Ekwueme, D. U., Virgo, K. S., Kent, E. E., Nutt, S., Zheng, Z., Rechis, R., & Yabroff, K. R. (2016). For working-age cancer survivors, medical debt and bankruptcy create financial hardships. *Health Affairs*, *35*(1), 54–61.** | | |
| Study Characteristics | Study Purpose | To assess the proportion of working-age cancer survivors who reported that they or their families had gone into debt (respondents also gave the amount of debt incurred) and the proportion who reported having filed for bankruptcy because of cancer, its treatment, or the lasting effects of that treatment. |
|  | Source of Data | Secondary analysis of pre-existing survey data |
|  | Study Type | Cross-sectional |
|  | Data Collection Geography | National |
|  | Population | Patients |
|  | Sample Size | 4,719 patients |
|  | Medical Condition | Cancer (all types) |
| Findings: Categories of PCBEOs | Categories of PCBEOs Examined | Direct medical costs  Intangible burdens |
|  | Direct Medical Costs | Medical financial debt |
|  | Direct Non-Medical Costs | Filed for bankruptcy |
|  | Intangible Burdens | Fear and anxiety about future ability to afford medical treatments |
| Findings: Differences in PCBEOs | Types of Differences Examined | Age, employment status, insurance, relationship status, race/ethnicity |
|  | Age | Compared with ages 55–64, ages 18–44 was associated with borrowing money/debt (odds ratio [OR]=2.07, *p*<0.001, 95% confidence interval [CI] [1.73, 2.47]) and bankruptcy (OR=1.81, *p*<0.05, 95% CI [1.10, 2.98]).  Compared with ages 55–64, ages 45–54 was associated with borrowing money/debt (OR=1.66, *p*<0.001, 95% CI [1.40, 1.98]) and bankruptcy (OR=1.86, *p*<0.05, 95% CI [1.15, 3.01]). |
|  | Employment Status | Compared with full-time employed patients, unemployed patients had higher odds of borrowing money/debt (OR=1.28, *p*<0.05, 95% CI [1.04, 1.57]) and bankruptcy (OR=1.74, *p*<0.05, 95% CI [1.04, 2.91]).  Compared with full-time employed patients, retired patients had lower odds of borrowing money/debt (OR=0.51, *p*<0.001, 95% CI [0.38, 0.70]). |
|  | Insurance | Compared with private insurance, public insurance (OR=1.95, *p*<0.001, 95% CI [1.50, 2.45]) and being uninsured (OR=2.46, *p*<0.001, 95% CI [1.64, 2.69]) are associated with borrowing money/debt, and public insurance is associated with bankruptcy (OR=1.82, *p*<0.05, 95% CI [1.08, 3.06]). |
|  | Relationship Status | Compared with married male patients, non-married male (OR=1.37, *p*<0.05, 95% CI [1.05, 1.79]) and non-married female patients (OR=1.56, *p*<0.001, 95% CI [1.22, 2.01]) had higher odds of borrowing money. |
|  | Race/Ethnicity | Compared with non-Hispanic white patients, Hispanic patients had higher odds of borrowing money (OR=1.41, *p*<0.05, 95% CI [1.06, 1.87]). |
| Findings: Strategies for Addressing Differences | Strategies for Addressing Differences | No |
|  | Methods/Instruments | Yes. LIVESTRONG Survey, which borrowed Medical Expenditure Panel Survey Experiences with Cancer Survivorship Supplement questions. |
| **Beeler, W. H., Bellile, E. L., Casper, K. A., Jaworski, E., Burger, N. J., Malloy, K. M., Spector, M. E., Shuman, A. G., Rosko, A., Stucken, C. L., Chinn, S. B., Dragovic, A. F., Chapman, C. H., Owen, D., Jolly, S., Bradford, C. R., Prince, M. E. P., Worden, F. P., Jagsi, R., ... Swiecicki, P. L. (2020). Patient-reported financial toxicity and adverse medical consequences in head and neck cancer. *Oral Oncology*, *101*, 104521.** | | |
| Study Characteristics | Study Purpose | To identify predictors of financial toxicity (FT) and adverse consequences in head and neck cancer after radiotherapy. |
|  | Source of Data | Primary quantitative data collected through a survey |
|  | Study Type | Cross-sectional |
|  | Data Collection Geography | Multiple sites in a single state |
|  | Population | Patients |
|  | Sample Size | 63 patients |
|  | Medical Condition | Head and neck cancer |
| Findings: Categories of PCBEOs | Categories of PCBEOs Examined | Intangible burdens |
|  | Intangible Burdens | Other intangible burdens: Composite FT score |
| Findings: Differences in PCBEOs | Types of Differences Examined | Age, geographic location |
|  | Age | Relative to individuals aged 65 or older, being younger than 65 was associated with a 5.65-point decrease in FT (β=-5.65, *p*=0.045, 95% confidence interval [CI] [-11.6, -0.13]), all else being equal. |
|  | Geographic Location | Relative to those with a household income greater than or equal to $50,000/year, a household income greater than $50,000/year was associated with a 6.81-point decrease in FT (β=06.81, *p*=0.016, 95% CI [-12.35, -1.28]), all else being equal. |
| Findings: Strategies for Addressing Differences | Strategies for Addressing Differences | No |
|  | Methods/Instruments | Comprehensive Score for financial Toxicity tool |
| **Berghuijs, K. M. V. T., Kaddas, H. K., Trujillo, G., Rouhani, G., Chevrier, A., Ose, J., Shibata, D., Toriola, A. T., Figueiredo, J. C., Peoples, A. R., Li, C. I., Hardikar, S., Siegel, E. M., Gigic, B., Schneider, M., Ulrich, C. M., & Kirchhoff, A. C. (2024). Age-related differences in employment, insurance, and financial hardship among colorectal cancer patients: A report from the ColoCare Study. *Journal of Cancer Survivorship*, *18*(3), 1075–1084.** | | |
| Study Characteristics | Study Purpose | To understand the employment, insurance, and financial experiences by age at diagnosis for colorectal cancer patients in the ColoCare Study. |
|  | Source of Data | Secondary analysis of pre-existing survey data |
|  | Study Type | Cross-sectional |
|  | Data Collection Geography | Not stated/unclear  United States |
|  | Population | Patients |
|  | Sample Size | 202 patients |
|  | Medical Condition | Colorectal cancer |
| Findings: Categories of PCBEOs | Categories of PCBEOs Examined | Intangible burdens |
|  | Intangible Burdens | Diminished household wealth, fear and anxiety about future ability to afford medical treatments |
| Findings: Differences in PCBEOs | Types of Differences Examined | Age, employment status |
|  | Age | Material financial hardship, borrowing money, debt, and so on were common for many patients across age, but greatest among the 18–49 age group.  Those in the 18–49 age group were more likely to put off major purchases (n=22, 52.4%, *p*<0.001), take on credit card debt (n=15, 35.7%, *p*=0.003), borrow money (n=12, 28.6%, *p*=0.003), and think about filing bankruptcy (n=7, 16.7%, *p*=0.004) than other age groups.  Material and psychological hardship summary scores were highest for younger patients compared with those in older age groups (18–49: Mean=2.2, 50–64: Mean=1.4, 65 or older: Mean=0.9).  Patients in the 18–49 age group (mean=2.3, *p*=0.02, 95% confidence interval [CI] [1.47, 3.59]) and the 50–64 age group (mean=1.6, *p*=0.02, 95% CI [1.09, 2.26]) were more likely to report psychological hardship (compared with those aged 65 or older). |
|  | Employment Status | Those working reported greater material hardship than those not working (mean=1.7, *p*=0.01). |
| Findings: Strategies for Addressing Differences | Strategies for Addressing Differences | The study suggests launching programs to help patients, specifically cancer patients, understand employment protections. |
|  | Methods/Instruments | No |
| **Berinstein, J. A., Cohen-Mekelburg, S. A., Steiner, C. A., McLeod, M., Noureldin, M., Allen, J. I., Kullgren, J. T., Waljee, A. K., & Higgins, P. D. R. (2021). Variations in health care utilization patterns among inflammatory bowel disease patients at risk for high medical service utilization enrolled in high-deductible health plans. *Inflammatory Bowel Diseases*, *27*(6), 771–778.** | | |
| Study Characteristics | Study Purpose | To quantify the out-of-pocket health care costs for inflammatory bowel disease patients at risk for high medical service utilization and to examine variations in health care service utilization throughout the year among high-deductible health plan and traditional health plan (i.e., private health insurance plans without a high deductible) enrollees. |
|  | Source of Data | Medical claims: Researchers used pre-existing data generated when health care providers submitted a request for payment to an insurer. |
|  | Study Type | Retrospective cohort: Longitudinal study using pre-existing data on two groups to examine the relationship between an exposure and an outcome |
|  | Data Collection Geography | National |
|  | Population | Patients |
|  | Sample Size | 13,052 patients |
|  | Medical Condition | Other: Irritable bowel disorder |
| Findings: Categories of PCBEOs | Categories of PCBEOs Examined | Direct medical costs |
|  | Direct Medical Costs | Out-of-pocket medical costs |
| Findings: Differences in PCBEOs | Types of Differences Examined | Insurance |
|  | Insurance | Patients with a high-deductible health plan paid a median annual coinsurance of $876 (vs. $524; *p*<0.001), median annual deductible of $1,483 (vs. $302; *p*<0.001), and median annual out-of-pocket cost of $2,870 (vs. $1,864; *p*<0.001) compared with those with a traditional health plan.  Conversely, the median annual copay was $102 in the high-deductible health plan group compared with $742 in the traditional health plan group (*p*<0.001).  Despite variable degrees of cost-sharing intended to reduce total health care costs, the median annual total health care expenses were similar in both groups (i.e., no statistically significant difference) (high-deductible health plan=$23,029 vs. traditional health plan=$23,794; *p*=0.628). |
| Findings: Strategies for Addressing Differences | Strategies for Addressing Differences | No |
|  | Methods/Instruments | No |
| **Boddu, S. P., Lin, E., Gill, V. S., Hinckley, N. B., Lai, C. H., & Renfree, K. J. (2024). Low-income, poor physical health, poor mental health, and other social risk factors are associated with decreased access to care in patients with carpal tunnel syndrome. *Journal of Primary Care & Community Health*, *15*.** | | |
| Study Characteristics | Study Purpose | To assess the association of carpal tunnel syndrome and patient-specific factors with decreased access to overall health care and health care utilization among patients with carpal tunnel syndrome. |
|  | Source of Data | Secondary analysis of pre-existing survey data |
|  | Study Type | Cross-sectional |
|  | Data Collection Geography | Multiple sites across multiple states |
|  | Population | Patients |
|  | Sample Size | 15,298 patients |
|  | Medical Condition | Other: Carpal tunnel syndrome |
| Findings: Categories of PCBEOs | Categories of PCBEOs Examined | Intangible burdens |
|  | Intangible Burdens | Delaying or forgoing medical treatments due to costs, other intangible burdens: Skipping medication |
| Findings: Differences in PCBEOs | Types of Differences Examined | Age, education, income, relationship status, sex/gender |
|  | Age | Older age was a significant predictor of skipping medications (odds ratio [OR]=0.98, 95% confidence interval [CI] [0.97,0.98], *p*<0.001).  Older age had a minor association with going over 1 year since seeing a health care provider (OR=0.97, *p*<0.001). |
|  | Educational Attainment | Not finishing a high school education was a significant predictor of going over 1 year since seeing a health care provider (OR=2.81, 95% CI [1.17, 6.73], *p*=0.02). |
|  | Income | Factors associated with increased likelihood of skipped medications included a moderate association with income less than $50,000 (OR=1.68, *p*<0.001).  Having income greater than $100,000 was strongly associated negatively with going over 1 year since seeing a health care provider (OR=0.4, *p*=0.002). |
|  | Relationship Status | A “single” marital status was a significant predictor of skipping medication (OR=0.84, *p*=0.02). |
|  | Sex | Males were less likely to skip medication compared with females (OR=0.63, *p*<0.001). |
| Findings: Strategies for Addressing Differences | Strategies for Addressing Differences | Interventions may include expanded insurance coverage, community resources assisting with transportation to and from clinic visits, and increased usage of Wide-Awake Local Anesthesia No Tourniquet Carpal Tunnel Release. |
|  | Methods/Instruments | Healthcare Access and Utilization Survey within the *All of Us* Database |
| **Choi, E., Berkman, A. M., Cheung, C. K., Betts, A. C., Salsman, J. M., Andersen, C. R., Ochoa-Dominguez, C. Y., Miller, K., Milam, J., Shah, A., Peterson, S. K., Lu, Q., Livingston, J. A., Hildebrandt, M. A. T., Parsons, S. K., Freyer, D., & Roth, M. E. (2023). Psychological distress and mental health care utilization among Hispanic/Latino survivors of adolescent and young adult cancer. *Psychooncology*, *32*(12), 1918–1929.** | | |
| Study Characteristics | Study Purpose | To compare psychological distress, mental health care use, and inability to afford mental health care between Hispanic/Latino survivors of adolescent and young adult cancer and Hispanic/Latino controls. |
|  | Source of Data | Secondary analysis of pre-existing survey data |
|  | Study Type | Cross-sectional |
|  | Data Collection Geography | National |
|  | Population | Patients |
|  | Sample Size | 4,070 |
|  | Medical Condition | Cancer (all types) |
| Findings: Categories of PCBEOs | Categories of PCBEOs Examined | Intangible burdens |
|  | Intangible Burdens | Delaying or forgoing medical treatments due to costs |
| Findings: Differences in PCBEOs | Types of Differences Examined | Relationship status |
|  | Relationship Status | The model-adjusted odds for being unable to afford mental health care among Hispanic/Latino survivors of adolescent and young adult cancer were higher compared with survivors who were married/living with a partner (odds ratio=7.23, p=0.010, 95% confidence interval [1.821, 28.276]). |
| Findings: Strategies for Addressing Differences | Strategies for Addressing Differences | No |
|  | Methods/Instruments | National Health Interview Survey data (2010–2018): Validated and publicly available |
| **Ciftci, Y., Radomski, S. N., Johnston, F. M., & Greer, J. B. (2024). Predictors of financial toxicity risk among patients undergoing cytoreductive surgery with hyperthermic intraperitoneal chemotherapy (CRS–HIPEC). *Annals of Surgical Oncology*, *31*(3), 1980–1989.** | | |
| Study Characteristics | Study Purpose | To use preoperative factors to estimate the risk of financial toxicity for patients undergoing cytoreductive surgery with hyperthermic intraperitoneal chemotherapy (CRS-HIPEC) at a single institution. |
|  | Source of Data | Primary quantitative data collected through a survey |
|  | Study Type | Retrospective cohort: Longitudinal study using pre-existing data on two groups to examine the relationship between an exposure and an outcome |
|  | Data Collection Geography | Single institution/site |
|  | Population | Patients |
|  | Sample Size | 163 patients |
|  | Medical Condition | Other: Patients undergoing CRS-HIPEC |
| Findings: Categories of PCBEOs | Categories of PCBEOs Examined | Intangible burdens |
|  | Intangible Burdens | Stress and anxiety directly caused by out-of-pocket costs, financial debt, or lost income (i.e., financial toxicity [FT]) |
| Findings: Differences in PCBEOs | Types of Differences Examined | Income |
|  | Income | The majority of patients in the lowest income quartile were determined to be at risk for FT (n=11, 78.6%), compared with 63.6% (n=14) in the second income quartile, 34.1% (n=14) in the third income quartile, and only 15.1% (n=13) in the highest income quartile (*p*<0.001). |
| Findings: Strategies for Addressing Differences | Strategies for Addressing Differences | The information in this study can be utilized to counsel patients and offer appropriate referrals to financial navigators prior to the surgery in hopes to mitigate some of the deleterious impacts of FT and improve patient well-being. |
|  | Methods/Instruments | No |
| **Cordova, F. M., Harris, R. B., Teufel-Shone, N. I., Nisson, P. L., Joshweseoma, L., Brown, S. R., Sanderson, P. R., Ami, D., Saboda, K., Mastergeorge, A. M., & Gerald, L. B. (2016). Caregiving on the Hopi Reservation: Findings from the 2012 Hopi survey of cancer and chronic disease. *Journal of Community Health*, *41*(6), 1177–1186.** | | |
| Study Characteristics | Study Purpose | To compare caregivers’ versus non-caregivers’ reported health and use of the health care system for chronic disease prevention. |
|  | Source of Data | Primary quantitative data collected through a survey |
|  | Study Type | Cross-sectional |
|  | Data Collection Geography | Single institution/site |
|  | Population | Caregivers |
|  | Sample Size | 802 caregivers |
|  | Medical Condition | Cancer (all types), other: disease or disability other than cancer |
| Findings: Categories of PCBEOs | Categories of PCBEOs Examined | Indirect impacts |
|  | Indirect Costs | Other indirect impacts: Financial burden |
| Findings: Differences in PCBEOs | Types of Differences Examined | Sex/gender |
|  | Sex | The only “greatest difficulty” that was statistically significant between males and females was “financial burden” (*p*=0.01). |
| Findings: Strategies for Addressing Differences | Strategies for Addressing Differences | No |
|  | Methods/Instruments | No |
| **Corkum, J., Zhu, V., Agbafe, V., Sun, S. X., Chu, C., Colen, J. S., Greenup, R., & Offodile, A. C. (2022). Area deprivation index and rurality in relation to financial toxicity among breast cancer surgical patients: Retrospective cross-sectional study of geospatial differences in risk profiles. *Journal of the American College of Surgeons*, *234*(5), 816–826.** | | |
| Study Characteristics | Study Purpose | To examine the relationship between geospatial location, represented by rurality and Area Deprivation Index (ADI), and risk of financial toxicity (FT). |
|  | Source of Data | Primary quantitative data collected through a survey |
|  | Study Type | Cross-sectional |
|  | Data Collection Geography | Single institution/site |
|  | Population | Patients |
|  | Sample Size | 568 patients |
|  | Medical Condition | Breast cancer |
| Findings: Categories of PCBEOs | Categories of PCBEOs Examined | Direct medical costs  Intangible burdens |
|  | Direct Medical Costs | Medical financial debt |
|  | Intangible Burdens | Delaying or forgoing medical treatments due to costs; diminished household wealth; reduced spending on basic goods, groceries, and leisure activities; other intangible burdens: Composite Comprehensive Score for financial Toxicity (COST) score |
| Findings: Differences in PCBEOs | Types of Differences Examined | Age, geographic location, relationship status |
|  | Age | Older age (odds ratio [OR]=0.98, 95% confidence interval [CI] [0.96, 0.99], *p*<0.01) was protective of FT. |
|  | Geographic Location | The study found significant association between patients’ ADI rankings and increasing risk of FT (OR=1.012, 95% CI [1.01, 1.02], *p*<0.01).  The study found a positive association between worsening ADI tertile and the following: Decreased basic spending, decreased spending on leisure activity, savings depletion to pay for cancer care, borrowed money to pay for care, and skipped a clinic visit to save costs (*p*<0.01). |
|  | Relationship Status | Marital status (OR=0.46, 95% CI [0.30, 0.70], *p*<0.01) was protective against FT. |
| Findings: Strategies for Addressing Differences | Strategies for Addressing Differences | Using ADI to prioritize resources to mitigate FT |
|  | Methods/Instruments | COST tool: Validated and publicly available |
| **Corrigan, K. L., Fu, S., Chen, Y. S., Kaiser, K., Roth, M., Peterson, S. K., Shih, Y. T., Jagsi, R., Giordano, S. H., Volk, R. J., Yabroff, K. R., Banegas, M. P., Acquati, C., Conti, R. M., Ma, H. Y., Ku, K., You, Y. N., & Smith, G. L. (2022). Financial toxicity impact on younger versus older adults with cancer in the setting of care delivery. *Cancer*, *128*(13), 2455–2462.** | | |
| Study Characteristics | Study Purpose | To quantify and compare the domains and severity of the material, coping, and psychological financial toxicity (FT) burdens early in the care and survivorship trajectory for working-age adults (including young adults aged 18–39 and other working-age adults aged 40–64) and older adults with cancer. |
|  | Source of Data | Secondary analysis of pre-existing survey data |
|  | Study Type | Cross-sectional |
|  | Data Collection Geography | Multiple sites in a single state |
|  | Population | Patients |
|  | Sample Size | 311 patients |
|  | Medical Condition | Cancer (all types) |
| Findings: Categories of PCBEOs | Categories of PCBEOs Examined | Intangible burdens |
|  | Intangible Burdens | Stress and anxiety directly caused by out-of-pocket costs, financial debt, or lost income (i.e., FT) |
| Findings: Differences in PCBEOs | Types of Differences Examined | Age, education, employment status, income, insurance, race/ethnicity |
|  | Age | Median overall FT score as well as material, coping, and psychological FT scores were significantly worse for individuals in younger age groups (18–39 and 40–64) compared with those aged 65 or older (Kruskal-Wallis *p*<0.001 for all comparisons).  Fifty percent of younger adults aged 18–39 had severe (score, ≥5) overall FT versus 40.7% of adults aged 40–64 and 9.6% of adults aged 65 or older (*p*<0.001).  There was increased likelihood of severe FT across younger groups for adults aged 18–39 (adjusted odds ratio [OR]=5.84, *p*<0.001, 95% confidence interval [CI] [2.07, 16.5]) and for adults aged 40–64 (adjusted OR=4.58, *p*<0.001, 95% CI [2.16, 9.72]), compared with the referent group of adults aged 65 or older.  Young adults also frequently encountered severe financial toxixity across the separate domains: 47% reported severe material, 47% severe coping, and 70% severe psychological FT burden. |
|  | Educational Attainment | Lower educational attainment was associated with greater FT (*p*<0.001), with the highest median FT score in the high school or GED and associate degree or trade certification (3.9) and the lowest median FT score among the advanced degree category (1.3). |
|  | Employment Status | Part-time and unemployed people had significantly higher FT scores (5.2 and 5.4, respectively) than people employed full-time (2.5) or retired (1.3) (*p*<0.001). |
|  | Income | Individuals with lower incomes had significantly higher FT scores than those with higher incomes. Specifically, individuals with incomes between $0 and $19,999 (FT: 5.3) and $20,000 and $49,999 (FT: 4.5) had significantly higher FT scores than individuals with income $50,000–$99,999 (FT: 2.9) and $100,000–$199,999 (FT: 1.8) and $200,000+ (1.2) (*p*<.001). |
|  | Insurance | Individuals with private insurance had significantly higher FT (employer based: 3.2, private marketplace purchased: 4.5) than those with Medicare (1.8). However, individuals with Medicaid/state or none had the highest FT scores (5.4) (*p*<.001). |
|  | Race/Ethnicity | Black, non-Hispanic (FT: 5) and Hispanic (4) respondents had significantly higher FT scores than White, non-Hispanic respondents (2) (*p*<0.001). |
| Findings: Strategies for Addressing Differences | Strategies for Addressing Differences | No |
|  | Methods/Instruments | Economic Strain and Resilience in Cancer: Whether the method/instrument is validated and/or publicly available was not mentioned. |
| **Cwalina, T. B., Jella, T. K., Tripathi, R., & Carroll, B. T. (2023). Financial stress among skin cancer patients: A cross-sectional review of the 2013–2018 National Health Interview Survey. *Archives of Dermatological Research*, *315*(4), 1003–1010.** | | |
| Study Characteristics | Study Purpose | To perform a cross-sectional review of the National Health Interview Survey to quantify the prevalence of various financial stress measures among skin cancer patients, identify subgroups affected most by personal financial stress, and isolate the most predictive features of skin cancer patients experiencing financial stress. |
|  | Source of Data | Secondary analysis of pre-existing survey data |
|  | Study Type | Cross-sectional |
|  | Data Collection Geography | National |
|  | Population | Patients |
|  | Sample Size | 5,698 patients |
|  | Medical Condition | Skin cancer |
| Findings: Categories of PCBEOs | Categories of PCBEOs Examined | Intangible burdens |
|  | Intangible Burdens | Fear and anxiety about future ability to afford medical treatments |
| Findings: Differences in PCBEOs | Types of Differences Examined | Age, education, income, insurance |
|  | Age | Older individuals were significantly more likely to report problems paying medical bills (estimate: 0.96, *p*<0.001, 95% confidence interval [CI] [0.95, 0.97]). |
|  | Educational Attainment | Patients without a high school diploma had increased odds (adjusted odds ratio [OR]=2.55, *p*<0.001, 95% CI [1.43, 4.57]) of having problems paying or being unable to pay medical bills in the past 12 months than patients with a graduate degree. |
|  | Income | Of patients with income below 200% of the poverty level, 33.84% (*p*<0.001, 95% CI [30.62%, 37.06%]) responded as being worried about medical costs. |
|  | Insurance | Uninsured patients had higher odds (adjusted OR=4.82, *p*<0.001, 95% CI [3.02, 7.70]) of being worried about medical costs than patients with private insurance. |
| Findings: Strategies for Addressing Differences | Strategies for Addressing Differences | No |
|  | Methods/Instruments | No |
| **Davis, E. S., Poulson, M. R., Yarbro, A. A., Franks, J. A., Bhatia, S., & Kenzik, K. M. (2024). Understanding racial differences in financial hardship among older adults surviving cancer. *Cancer*, *130*(20), 3487–3495.** | | |
| Study Characteristics | Study Purpose | To study racial differences in financial hardship as a function of the downstream social determinants of health intrinsic to structural racism that help explain these disparities. |
|  | Source of Data | Secondary analysis of pre-existing survey data |
|  | Study Type | Cross-sectional |
|  | Data Collection Geography | Single institution/site |
|  | Population | Patients |
|  | Sample Size | 721 patients |
|  | Medical Condition | Breast cancer, colorectal cancer, leukemia and lymphoma, lung cancer, prostate cancer |
| Findings: Categories of PCBEOs | Categories of PCBEOs Examined | Indirect impacts  Intangible burdens |
|  | Indirect Costs | Other indirect impacts: Material hardship |
|  | Intangible Burdens | Stress and anxiety directly caused by out-of-pocket costs, financial debt, or lost income (i.e., financial toxicity) |
| Findings: Differences in PCBEOs | Types of Differences Examined | Income, race/ethnicity |
|  | Income | Income was the largest contributor to Black–White differences in any financial hardship (21%) and in material (25%) and psychological (25%) financial hardship. |
|  | Race/Ethnicity | Black patients reported higher rates of any financial hardship (40.9% vs. 19.2%; *p*<0.0001) and higher rates of material hardship (29.6% vs.11.4%; *p*<0.0001) and psychological hardship (27.2% vs. 11.4%; *p*<0.0001) compared with their White counterparts.  Black patients reported experiencing more domains (greater in tensity) of financial hardship than White patients (*p*<0.0001), with 16.7% of Black patients reporting two domains of hardship (vs. 4.7% of White patients) and 2.6% reporting three domains of hardship (vs. 2.2% of White patients). |
| Findings: Strategies for Addressing Differences | Strategies for Addressing Differences | Quality improvement efforts at the patient, provider, and policy levels include systematic screening for financial hardship, connecting patients to financial navigation programs, documented discussions between providers and patients regarding costs of care, and increased data collection and documentation regarding patient‐reported financial hardship and its effect on patient outcomes. |
|  | Methods/Instruments | Financial hardship questions were from the Medical Expenditure Panel Survey: Publicly available and validated |
| **Doherty, M., Gardner, D., & Finik, J. (2021). The financial coping strategies of US cancer patients and survivors. *Supportive Care in Cancer*, *29*(10), 5753–5762.** | | |
| Study Characteristics | Study Purpose | To identify distinct problem-focused financial coping strategies, determine whether individuals sorted into “coping classes” by employing different combinations of financial coping strategies, and measure the extent to which sociodemographic factors were associated with coping class membership. |
|  | Source of Data | Primary quantitative data collected through a survey |
|  | Study Type | Cross-sectional |
|  | Data Collection Geography | National |
|  | Population | Patients |
|  | Sample Size | 510 patients |
|  | Medical Condition | Cancer (all types) |
| Findings: Categories of PCBEOs | Categories of PCBEOs Examined | Direct non-medical costs  Intangible burdens |
|  | Direct Medical Costs | Medical financial debt |
|  | Direct Non-Medical Costs | Filed for bankruptcy |
|  | Intangible Burdens | Delaying or forgoing medical treatments due to costs, food insecurity, housing insecurity, reduced spending on basic goods, groceries, and leisure activities |
| Findings: Differences in PCBEOs | Types of Differences Examined | Age, race/ethnicity |
|  | Age | Those aged 25–34 had higher odds of being in the high burden/high coping class (odds ratio [OR]=9.87, 95% confidence interval [CI] [8.59, 11.14], *p*<0.01) and higher odds of being in the high self-advocacy class (OR=3.10, 95% CI [2.05, 4.16], *p*<0.05, compared with those aged 65–74.  Those aged 35–44 had greater odds of being in the high burden/high coping class (OR=12.27, 95% CI [7.03, 19.87], *p*<0.01) and greater odds of being in the high self-advocacy class (OR=7.08, 95% CI [5.89, 8.28], *p*<0.01), compared with those aged 65–74.  Those aged 45–54 had higher odds of being in the high burden/high coping class (OR=7.58, 95% CI [6.35, 8.81], *p*<0.01), compared with those aged 65–74.  Those aged 55–64 had higher odds of being in the high burden/high coping class (OR=4.11, 95% CI [2.91, 5.93], *p*<0.05), compared with those aged 65–74. |
|  | Race/Ethnicity | Compared with white respondents, African American (OR=5.82, 95% CI [3.01, 6.64], *p*<0.01) and Hispanic (OR=5.60, 95% CI [4.40, 6.79], *p*<0.01) respondents had nearly 6 times higher odds of belonging to the high burden/high coping class than the low burden/low coping class.  African American respondents were also at increased odds of belonging to the high self-advocacy class than the low burden/low coping group, when compared with White respondents (OR=1.99, 95% CI [1.19, 2.80], *p*<0.10). |
| Findings: Strategies for Addressing Differences | Strategies for Addressing Differences | No |
|  | Methods/Instruments | 41-item designed survey: Whether the method/instrument is validated and/or publicly available was not mentioned. |
| **Evans, E. M., Lin, J., Sanchez‐Alvarez, J., Agrawal, A. K., & Winestone, L. E. (2023). Disparities in household material hardship, financial toxicity, and income loss in pediatric cancer. *Pediatric Blood & Cancer*, *70*(9), e30496.** | | |
| Study Characteristics | Study Purpose | To examine the prevalence of household material hardship (HMH), financial toxicity (FT), and income loss in English- and Spanish-speaking caregivers of children with cancer and the impact of work flexibility and social support on financial burden in this population. |
|  | Source of Data | Primary quantitative data collected through a survey |
|  | Study Type | Cross-sectional |
|  | Data Collection Geography | Single institution/site |
|  | Population | Caregivers |
|  | Sample Size | 156 caregivers |
|  | Medical Condition | Pediatric cancer |
| Findings: Categories of PCBEOs | Categories of PCBEOs Examined | Indirect impacts |
|  | Indirect Costs | Other indirect impacts: Household material hardships |
| Findings: Differences in PCBEOs | Types of Differences Examined | Income, race/ethnicity |
|  | Income | Low- and middle-income caregivers were more likely to experience HMH and FT compared with high-income caregivers (HMH: 68% low vs. 38% middle vs. 8.7% high, *p*<0.001; FT: 81% vs. 68% vs. 44%, *p*<0.001). |
|  | Race/Ethnicity | Hispanic caregivers were more likely to report HMH compared with non-Hispanic White and Asian caregivers (HMH: 57% vs. 21% vs. 19%, *p*<0.001). |
| Findings: Strategies for Addressing Differences | Strategies for Addressing Differences | No |
|  | Methods/Instruments | No |
| **Fusco, K. N., Isoba, L. C. G., Alef, R., Roger, A., & Mayrovitz, H. N. (2024). Effects of demographic identities on psychosocial burdens of patients living in the United States with systemic lupus erythematosus. *Cureus*, *16*(12).** | | |
| Study Characteristics | Study Purpose | To investigate the potential role or relationship between demographic identities of age, race/ethnicity, and geographic location on the psychosocial burdens of care accessibility, finance, mental health, and social support on one specific health condition, systemic lupus erythematosus (SLE). |
|  | Source of Data | Primary quantitative data collected through a survey |
|  | Study Type | Cross-sectional |
|  | Data Collection Geography | National |
|  | Population | Patients |
|  | Sample Size | 294 patients |
|  | Medical Condition | Autoimmune diseases: SLE |
| Findings: Categories of PCBEOs | Categories of PCBEOs Examined | Direct medical costs |
|  | Direct Medical Costs | Out-of-pocket medical costs |
| Findings: Differences in PCBEOs | Types of Differences Examined | Age, geographic location, race/ethnicity |
|  | Age | Respondents older than 61 were more likely to spend more than $200 a month on prescriptions for all their medical conditions than their younger counterparts (33.3% vs. 9.4%–11.4% for other age groups). |
|  | Geographic Location | Suburban and urban respondents also reported receiving more support than rural ones. Of respondents, 36.8% urban and 38.2% suburban vs. 19.4% rural reported a lot of support felt from friends, family, or peers regarding SLE diagnosis. |
|  | Race/Ethnicity | About 20.1% of the respondents indicated that they are unable to afford their copay for all their monthly medications in general. Indian Asians were noted to pay more out-of-pocket medical expenses compared with other race/ethnicity groups: 67% paid $5,000–$10,000 (compared with 0%–16.7% for other races), and 33.3% paid more than $10,000 (compared with 0%–9.5% for other races). |
| Findings: Strategies for Addressing Differences | Strategies for Addressing Differences | No |
|  | Methods/Instruments | No |
| **Ganapathy, V., Graham, G. D., DiBonaventura, M. D., Gillard, P. J., Goren, A., & Zorowitz, R. D. (2015). Caregiver burden, productivity loss, and indirect costs associated with caring for patients with poststroke spasticity. *Clinical Interventions in Aging*, 1793–1802.** | | |
| Study Characteristics | Study Purpose | To assess and quantify the economic burden of providing care for stroke survivors with poststroke spasticity. |
|  | Source of Data | Primary quantitative data collected through a survey |
|  | Study Type | Cross-sectional |
|  | Data Collection Geography | National |
|  | Population | Caregivers |
|  | Sample Size | 153 caregivers |
|  | Medical Condition | Stroke |
| Findings: Categories of PCBEOs | Categories of PCBEOs Examined | Indirect impacts |
|  | Indirect Costs | Unpaid time off work, early retirement, other indirect impacts: Presenteeism (restriction while at work) |
| Findings: Differences in PCBEOs | Types of Differences Examined | Age, income |
|  | Age | Age was associated with -0.008 marginal effect on absenteeism (*p*<0.0001) and -0.01 marginal effect on overall work restriction (*p*=0.004). |
|  | Income | Compared with income of $75,000 or more, income less than $25,000 was associated with 0.12 marginal effect on absenteeism (*p*=0.02) and a -0.31 marginal effect on presenteeism (*p*=0.011). Compared with income of $75,000 or more, income of $25,000–$49,999 was associated with 0.060 marginal effect on absenteeism (*p*=0.041). |
| Findings: Strategies for Addressing Differences | Strategies for Addressing Differences | No |
|  | Methods/Instruments | Work Productivity and Activity Impairment: Validated |
| **Garcia, M. A., Diminich, E. D., Lu, P., Arévalo, S. P., Sayed, L., Abdelrahim, R., & Ajrouch, K. J. (2023). Caregiving for foreign-born older adults with dementia. *Journals of Gerontology: Series B*, *78*(Supplement_1), S4–S14.** | | |
| Study Characteristics | Study Purpose | To examine how nativity, dementia classification, and age of migration of older foreign-born adults are associated with caregiver psychological well-being and care burden. |
|  | Source of Data | Secondary analysis of pre-existing survey data |
|  | Study Type | Cross-sectional |
|  | Data Collection Geography | National |
|  | Population | Caregivers |
|  | Sample Size | 1,474 caregivers |
|  | Medical Condition | Dementia, older adults, general |
| Findings: Categories of PCBEOs | Categories of PCBEOs Examined | Indirect impacts  Intangible burdens |
|  | Indirect Costs | Time spent providing unpaid caregiving |
|  | Intangible Burdens | Quality-of-life impacts (not quality-adjusted life-years or disability-adjusted life-years) |
| Findings: Differences in PCBEOs | Types of Differences Examined | Education, relationship status, race/ethnicity, sex/gender |
|  | Educational Attainment | In the regression model, some college (-0.53, *p*<0.001) and 4-year college degree or above (-0.28, *p*=0.037) were associated with lower average care burden compared with less than high school, all else being equal. |
|  | Relationship Status | In the regression model, never-married caregivers were associated with lower average care burden compared with married/partnered caregivers (-0.49, *p*=0.003), all else being equal. |
|  | Race/Ethnicity | In the regression model, non-Hispanic other race caregivers were associated with higher average care burden compared with non-Hispanic White caregivers (0.66, *p*=0.008), all else being equal. |
|  | Sex | In the regression model, male caregivers were associated with lower average care burden (-0.23, *p*=0.029) compared with female caregivers, all else being equal. |
| Findings: Strategies for Addressing Differences | Strategies for Addressing Differences | No |
|  | Methods/Instruments | National Health and Aging Trends Study data—used a 4-item scale for care burden: Publicly available |
| **Grafova, I. B., Devine, K. A., Hudson, S. V., O’Malley, D., Paddock, L. E., Bandera, E. V., Llanos, A. A. M., Fong, A. J., Evens, A. M., & Manne, S. (2024). Household income and county income inequality are associated with financial hardship among cancer survivors in New Jersey. *Journal of Cancer Survivorship*, 1–13.** | | |
| Study Characteristics | Study Purpose | To examine how household income and county income inequality are linked to financial hardship among cancer survivors. |
|  | Source of Data | Primary quantitative data collected through a survey |
|  | Study Type | Cross-sectional |
|  | Data Collection Geography | Multiple sites in a single state |
|  | Population | Patients |
|  | Sample Size | 864 patients |
|  | Medical Condition | Cancer (all types) |
| Findings: Categories of PCBEOs | Categories of PCBEOs Examined | Direct medical costs  Intangible burdens |
|  | Direct Medical Costs | Medical financial debt |
|  | Intangible Burdens | Delaying or forgoing medical treatments due to costs, stress and anxiety directly caused by out-of-pocket costs, financial debt, or lost income (i.e., financial toxicity) |
| Findings: Differences in PCBEOs | Types of Differences Examined | Age, education, income, insurance, rrelationship status, race/ethnicity, sex/gender |
|  | Age | Age was negatively associated with ever unable to cover cancer care out-of-pocket (average marginal effect=-0.004, *p*=0.001, 95% confidence interval [CI] [-0.007, -0.002]), unmet need in paying non-medical bills related to cancer (average marginal effect=-0.003, *p*=0.003, 95% CI [-0.005, -0.001]), unmet need in paying for follow-up/medications related to cancer (average marginal effect=-0.004, *p*=0.001, 95% CI [-0.006, -0.002]), and ever worried about having to pay large cancer medical bills (average marginal effect=-0.008, *p*<0.001, 95% CI [-0.012, -0.005).  Age was negatively associated with forgoing doctor visits (average marginal effect=-0.003, *p*=0.001, 95% CI [-0.005, -0.001]) and eye care or glasses/contacts (average marginal effect=-0.003, *p*=0.015, 95% CI [-0.006, -0.001]) due to cost in the past 12 months. |
|  | Educational Attainment | Having a college degree was negatively associated with unmet need in paying non-medical bills related to cancer (average marginal effect=-0.081, *p*<0.001, 95% CI [-0.125, -0.036]) and unmet need in paying for follow-up/medications related to cancer (average marginal effect=-0.062, *p*=0.005, 95% CI [-0.125, -0.019]).  Having a college degree was negatively associated with forgoing medications (average marginal effect=-0.082, *p*<0.001, 95% CI [-0.126, -0.037]) and dental care (average marginal effect=-0.064, *p*= 0.012, 95% CI [-0.114, -0.014]) due to cost in the past 12 months. |
|  | Income | Compared with households with an annual income at or above $90,000 (higher income), households with income less than $49,999 (lower income) had a higher risk of ever unable to cover cancer care out-of-pocket (average marginal effect=0.123, *p*=0.001, 95% CI [0.054, 0.192]. |
|  | Insurance | Compared with private insurance, having Medicare was negatively associated with unmet need in paying non-medical bills related to cancer (average marginal effect=-0.065, *p*=0.019, 95% CI [-0.119, -0.011]) and ever worried about having to pay large cancer medical bills (average marginal effect=-0.099, *p*=0.020, 95% CI [-0.182, -0.016]).  Compared with private insurance, having Medicaid was negatively associated with ever worried about having to pay large cancer medical bills (average marginal effect=-0.387, *p*<0.001, 95% CI [-0.580, -0.194]).  Compared with private insurance, having Medicaid was negatively associated with forgoing dental care in the past 12 months due to cost (average marginal effect=-0.232, *p*=0.002, 95% CI [-0.38, -0.084]). |
|  | Relationship Status | Being married was positively associated with ever worried about having to pay large cancer medical bills (average marginal effect=0.112, *p*=0.002, 95% CI [0.112, 0.182]). |
|  | Race/Ethnicity | Compared with White non-Hispanic, being Hispanic was positively associated with unmet need in paying non-medical bills related to cancer (average marginal effect=0.095, *p*=0.007, 95% CI [0.026, 0.164]).  Compared with being White, being other race non-Hispanic was positively associated with ever unable to cover cancer care out-of-pocket (average marginal effect=0.084, *p*=0.022, 95% CI [0.012, 0.156]), unmet need in paying non-medical bills related to cancer (average marginal effect=0.104, *p*=.002, 95% CI [0.038, 0.171]), unmet need in paying for follow-up/medications related to cancer (average marginal effect=0.075, *p*=.024, 95% CI [0.01, 0.14]), and ever worried about having to pay large cancer medical bills (average marginal effect=0.024 *p*=.021, 95% CI [0.019 .240]).  Compared with being White, being other race (non-Hispanic) was associated with a higher risk of forgoing doctor care (average marginal effect=0.107, *p*<0.001, 95% CI [0.060, 0.115]), medications (average marginal effect=0.067, *p*=0.036, 95% CI [0.004, 0.129]), dental care (average marginal effect=0.088, *p*=0.031, 95% CI [0.008, 0.168]), and eyecare or glasses/contacts (average marginal effect=0.08, *p*=0.025, 95% CI [0.010, 0.150]) due to cost in the past 12 months. |
|  | Sex | Being female was associated with forgoing mental health care or counseling due to cost in the past 12 months (average marginal effect=0.048, *p*=0.043, 95% CI [0.001, 0.094]). |
| Findings: Strategies for Addressing Differences | Strategies for Addressing Differences | Include middle household income survivors in financial navigation and mitigation programs. |
|  | Methods/Instruments | The study uses a self-designed survey to capture financial hardship.  Whether the method/instrument is validated and/or publicly available was not mentioned. |
| **Grafova, I. B., Manne, S. L., Hudson, S. V., Elliott, J., Llanos, A. A., Saraiya, B., & Duberstein, P. R. (2023). Functional impairment is associated with medical debt in male cancer survivors and credit card debt in female cancer survivors. *Supportive Care in Cancer*, *31*(10), 605.** | | |
| Study Characteristics | Study Purpose | To examine the associations of functional limitations with medical and credit card debt among cancer survivor families and explore sex differences in these associations. |
|  | Source of Data | Secondary analysis of pre-existing survey data |
|  | Study Type | Cross-sectional |
|  | Data Collection Geography | National |
|  | Population | Both |
|  | Sample Size | 723 patients and caregivers |
|  | Medical Condition | Cancer (all types) |
| Findings: Categories of PCBEOs | Categories of PCBEOs Examined | Direct medical costs |
|  | Direct Medical Costs | Medical financial debt |
| Findings: Differences in PCBEOs | Types of Differences Examined | Sex/gender |
|  | Sex | Medical debt was less common among families with female cancer survivors than male survivors (6.0% vs. 9.9%; *p*=0.048). |
| Findings: Strategies for Addressing Differences | Strategies for Addressing Differences | No |
|  | Methods/Instruments | Instrumental activities of daily living. Whether the method/ instrument is validated and/or publicly available was not mentioned. |
| **Gu, C., Jewett, P. I., Yabroff, K. R., Vogel, R. I., Parsons, H. M., Gangnon, R. E., Purani, H., & Blaes, A. H. (2024). Forgoing physician visits due to cost: Regional clustering among cancer survivors by age, sex, and race/ethnicity. *Journal of Cancer Survivorship*, *18*(2), 385–397.** | | |
| Study Characteristics | Study Purpose | To explore the prevalence of forgoing physician visits due to cost among cancer survivors by demographic characteristics and U.S. geographic regions in recent years. |
|  | Source of Data | Secondary analysis of pre-existing survey data |
|  | Study Type | Cross-sectional |
|  | Data Collection Geography | National |
|  | Population | Patients |
|  | Sample Size | 217,981 patients |
|  | Medical Condition | Cancer (all types) |
| Findings: Categories of PCBEOs | Categories of PCBEOs Examined | Intangible burdens |
|  | Intangible Burdens | Delaying or forgoing medical treatments due to costs |
| Findings: Differences in PCBEOs | Types of Differences Examined | Geographic location |
|  | Geographic Location | The prevalence of forgoing physician visits due to cost was highest in the South (North Central/Midwest vs. South, odds ratio [OR]=0.63, 95% confidence interval [CI] [0.56, 0.71]; Northeast vs. South, OR=0.63, 95% CI [0.55, 0.73]; West vs. South, OR=0.73, 95% CI [0.64, 0.84]). |
| Findings: Strategies for Addressing Differences | Strategies for Addressing Differences | The discussion section suggests that elimination of disproportionate cancer-related financial hardship for these and other vulnerable groups will require changes in the way care is delivered and financed through Medicaid and other health insurance reforms. |
|  | Methods/Instruments | 2015–2019 Behavioral Risk Factor Surveillance System survey question of forgoing physician visits due to cost: Validated and publicly available |
| **Hallgren, E., Moore, R., Ayers, B. L., Purvis, R. S., Bryant-Smith, G., DelNero, P., & McElfish, P. A. (2024). “It was kind of a nightmare, it really was:” Financial toxicity among rural women cancer survivors. *Journal of Cancer Survivorship*, *18*(3), 1006–1015.** | | |
| Study Characteristics | Study Purpose | To examine how rural female cancer survivors experience and manage financial toxicity. |
|  | Source of Data | Qualitative interviews |
|  | Study Type | Qualitative study |
|  | Data Collection Geography | Multiple sites in a single state |
|  | Population | Patients |
|  | Sample Size | 36 patients |
|  | Medical Condition | Cancer (all types) |
| Findings: Categories of PCBEOs | Categories of PCBEOs Examined | Direct medical costs  Intangible burdens |
|  | Direct Medical Costs | Medical financial debt |
|  | Intangible Burdens | Food insecurity, housing insecurity, reduced spending on basic goods, groceries, and leisure activities |
| Findings: Differences in PCBEOs | Types of Differences Examined | Insurance, relationship status |
|  | Insurance | Most survivors who reported no medical debt but did report difficulty affording basic living expenses had public health insurance.  Their insurance or state programs covered most of their medical expenses; however, preexisting financial precarity and employment changes related to cancer impacted their financial situation.  Survivors who reported medical debt primarily had employer-based private health insurance. |
|  | Relationship Status | Most survivors who reported no medical debt but did report difficulty affording basic living expenses were not married. Most survivors who reported medical debt but were able to meet basic needs were married. |
| Findings: Strategies for Addressing Differences | Strategies for Addressing Differences | The study notes that the findings point to the need to limit patient cost sharing in health insurance plans, so patients do not shoulder a large portion of cancer treatment costs beyond their means. |
|  | Methods/Instruments | No |
| **Hallinan, S., Sobering, J., Cardona-Martell, I., Girardin, M., Gohagon, K., O’Leary, D., & Stewart, G. (2024). Care recipient factors that predict caregiver burden in older adult dyads. *Home Health Care Management & Practice*, *36*(3), 191–196.** | | |
| Study Characteristics | Study Purpose | To identify factors that impact caregiver burden within caregiver dyads enrolled in one of the Veteran Affairs Home-Based Primary Care program teams to help facilitate effective support for the caregiver. |
|  | Source of Data | Secondary analysis of pre-existing survey data |
|  | Study Type | Retrospective cohort: Longitudinal study using pre-existing data on two groups to examine the relationship between an exposure and an outcome |
|  | Data Collection Geography | Multiple sites across multiple states |
|  | Population | Both |
|  | Sample Size | 201 veterans and their caregivers |
|  | Medical Condition | Medical condition not specified |
| Findings: Categories of PCBEOs | Categories of PCBEOs Examined | Intangible burdens |
|  | Intangible Burdens | Other intangible burdens |
| Findings: Differences in PCBEOs | Types of Differences Examined | Age, sex/gender |
|  | Age | The model found that age had a significant negative relationship with caregiver burden (estimate=-0.07, *p*=0.01), such that, as age increased, caregiver burden decreased. |
|  | Sex | The model found that gender was a significant predictor of caregiver burden (estimate=3.85, *p*=0.01), such that women were more likely than men to experience caregiver burden. The literature notes that there was an unbalanced sample in the population of men versus women. |
| Findings: Strategies for Addressing Differences | Strategies for Addressing Differences | No |
|  | Methods/Instruments | Zarit Burden Interview-12  Saint Louis University Mental Status  Braden Scale  Missouri Alliance for Home Care (MAHC-10)  Geriatric Depression Scale-Short Form |
| **Hastert, T. A., Banegas, M. P., Hamel, L. M., Reed, A. R., Baird, T., Beebe-Dimmer, J. L., & Schwartz, A. G. (2019). Race, financial hardship, and limiting care due to cost in a diverse cohort of cancer survivors. *Journal of Cancer Survivorship*, *13*(3), 429–437.** | | |
| Study Characteristics | Study Purpose | To estimate prevalence of types of cancer-related financial hardship by race and test whether they are associated with limiting care due to cost. |
|  | Source of Data | Primary quantitative data collected through a survey |
|  | Study Type | Cross-sectional |
|  | Data Collection Geography | Single institution/site |
|  | Population | Patients |
|  | Sample Size | 994 patients |
|  | Medical Condition | Breast cancer, colorectal cancer, lung cancer, prostate cancer |
| Findings: Categories of PCBEOs | Categories of PCBEOs Examined | Direct medical costs  Indirect impacts  Intangible burdens |
|  | Direct Medical Costs | Medical financial debt |
|  | Indirect Costs | Unpaid time off work, early retirement |
|  | Intangible Burdens | Delaying or forgoing medical treatments due to costs, diminished household wealth |
| Findings: Differences in PCBEOs | Types of Differences Examined | Age, education, employment status, income, insurance, relationship status, race/ethnicity, sex/gender |
|  | Age | Any financial hardship was associated with younger age (younger than 55=57.0%, 55–59=51.4%, 60–64=48.4%, 65 or older=31.5%, *p*<0.001). |
|  | Educational Attainment | Education status was associated with any financial hardship (*p*=0.007) with less than high school (38.7%) and college degree (39%) reporting less financial hardship than high school/GED (48.6%) and some college (52.0%). |
|  | Employment Status | Employment status was associated with any financial hardship (*p*=0.001) and any limiting medical care (*p*=0.023), with unemployed patients reporting the highest financial hardship (61.9%) and limiting medical care (24.4%).  Homemakers reported the lowest financial hardship (37.5%) and employed full time reported the lowest limiting care (11.8%). |
|  | Income | Lower income was associated with any financial hardship (53.6%, less than $20,000; 50.0%, $20,000–$39,999; 41.7%, $40,000–$59,999; 49.3%, $60,000–$79,999; 30.8%, $80,000 or more, *p*<0.001) and any limiting care due to cost (26.6%, less than $20,000; 17.7%, $20,000–$39,999; 12.5%, $40,000–$59,000; 13.2%, $60,000–$79,000; 5.5%, $80,000 or more, *p*<0.001). |
|  | Insurance | Not having private health insurance (*p*<0.001), not having Medicare (*p*<0.001), and having Medicaid (*p*=0.002) were associated with any financial hardship. Not having private health insurance (*p*<0.001) and having Medicaid (*p*=0.002) were associated with limiting care. |
|  | Relationship Status | Being married/living with partner was associated with lower financial hardship (39.1% vs. 51.5%–54.3%, *p*<0.001) and lower limiting care due to cost (12.1% vs. 20.8%–27.3%, *p*<0.001). |
|  | Race/Ethnicity | More African American than white survivors reported financial hardship (50.3% vs. 41.0%, *p*=0.005). More African American than White survivors reported limiting care (20.0% vs. 14.2%, *p*=0.019), driven by a higher proportion reporting that they needed to see a doctor but did not go due to cost (13.6% vs. 8.2%, *p*=0.008).  More White than African American survivors reported utilizing assets (9.3% vs. 4.8%, *p*=0.006), whereas more African American survivors reported cancer-related debt (30.5% vs. 18.5%, *p*<0.001).  These differences were evident for refinancing or selling a home (1.7% of White vs. 0.3% of African American survivors, *p*=0.026) and withdrawing money from retirement savings to pay for cancer care (7.5% of White vs. 4.3% of African American survivors, *p*=0.028). |
|  | Sex | Being female was associated with any financial hardship (50.6% vs 39.8%, *p*=0.001). |
| Findings: Strategies for Addressing Differences | Strategies for Addressing Differences | No |
|  | Methods/Instruments | Survey with previously designed questionnaire |
| **Jella, T. K., Cwalina, T. B., Sachdev, R., Otteson, T., & Fowler, N. (2021). Prevalence, trends, and demographic characteristics associated with self-reported financial stress among head and neck cancer patients in the United States of America. *American Journal of Otolaryngology*, *42*(6), 103154**. | | |
| Study Characteristics | Study Purpose | (1) To quantify the prevalence of self-reported financial insecurity among head and neck cancer patients, (2) to identify patient subgroups most affected by self-reported financial insecurity, and (3) to identify the demographic and socioeconomic patient characteristics associated with self-reported financial insecurity. |
|  | Source of Data | Secondary analysis of pre-existing survey data |
|  | Study Type | Cross-sectional |
|  | Data Collection Geography | National |
|  | Population | Patients |
|  | Sample Size | 710 patients |
|  | Medical Condition | Head and neck cancer |
| Findings: Categories of PCBEOs | Categories of PCBEOs Examined | Direct medical costs |
|  | Direct Medical Costs | Medical financial debt |
| Findings: Differences in PCBEOs | Types of Differences Examined | Income, insurance |
|  | Income | In multivariable logistic regression analysis to control for confounding factors, poverty status (adjusted odds ratio [aOR]=2.55, 95% confidence interval [CI] [1.48, 4.37], *p*=0.017) was determined as a statistically significant predictor for self-reported difficulty or inability to pay medical bills among head and neck cancer patients. |
|  | Insurance | In multivariable logistic regression analysis to control for confounding factors, insurance status (aOR=2.17, 95% CI [1.15, 4.07], *p*<0.001) was determined as a statistically significant predictor for self-reported difficulty or inability to pay medical bills among head and neck cancer patients |
| Findings: Strategies for Addressing Differences | Strategies for Addressing Differences | No |
|  | Methods/Instruments | Used data from the National Health Interview Survey: Publicly available and validated |
| **Jella, T. K., Cwalina, T. B., Treisman, J., & Hamadani, M. (2021). Risk factors for cost-related delays to medical care among lymphoma patients: A 22-year analysis of a nationally representative sample. *Clinical Lymphoma Myeloma and Leukemia*, *21*(7), e619–e625.** | | |
| Study Characteristics | Study Purpose | (1) To identify at-risk individuals who are likely to have delays in care due to cost-related concerns, insurance or otherwise; (2) to identify the patient characteristics, including socioeconomic and demographic patterns; and (3) to quantify the prevalence of financial stressors in this subpopulation of lymphoma patients. |
|  | Source of Data | Secondary analysis of pre-existing survey data |
|  | Study Type | Cross-sectional |
|  | Data Collection Geography | National |
|  | Population | Patients |
|  | Sample Size | 1,619 patients |
|  | Medical Condition | Leukemia and lymphoma |
| Findings: Categories of PCBEOs | Categories of PCBEOs Examined | Intangible burdens |
|  | Intangible Burdens | Delaying or forgoing medical treatments due to costs |
| Findings: Differences in PCBEOs | Types of Differences Examined | Age, income, insurance, relationship status, sex/gender, other |
|  | Age | In multivariable logistical regression analysis, the age groups of 25–44 and 45–64 had 4.63 times (95% confidence interval [CI] [2.28, 9.41]) and 5.85 times (95% CI [3.20,10.70]) greater odds, respectively, of experiencing medical delays due to cost (*p*<0.001) compared with 65 or older. Those aged 45–64 had 4.87 times (95% CI [2.33, 10.17], *p*<0.001) the odds of not being able to afford medical care. |
|  | Income | Patients whose federal poverty level was between 100% and 200% had greater odds of delaying care compared with respondents above 200% (aOR=2.30, 95% CI [1.26, 4.41], *p*=0.008). |
|  | Insurance | Patients without insurance had greater odds of not being able to afford medical care (aOR=3.16, 95% CI [1.25, 7.98], *p*=0.015) compared with those with private insurance. |
|  | Relationship Status | Single respondents had an aOR of 1.88 (95% CI [1.18, 3.00], *p*=0.009) of experiencing medical delays due to cost. |
|  | Sex | Females compared with males had significantly greater odds of delaying (aOR=1.62, 95% CI [1.06, 2.48], *p*=0.027) or being unable to afford medical care (aOR=2.20, 95% CI [1.28, 3.76], *p*=0.004). |
|  | Other | Immigration: Patients who were not born in the United States had significantly lower odds of delaying (aOR=0.27, 95% CI [0.09, 0.83], *p*=0.024) or being unable to afford medical care (aOR=0.14, 95% CI [0.02, 0.88], *p*=0.037). |
| Findings: Strategies for Addressing Differences | Strategies for Addressing Differences | No |
|  | Methods/Instruments | National Health Interview Survey questions: Publicly available and validated |
| **Jessup, N. M., Bakas, T., McLennon, S. M., & Weaver, M. T. (2015). Are there gender, racial or relationship differences in caregiver task difficulty, depressive symptoms and life changes among stroke family caregivers? *Brain Injury*, *29*(1), 17–24.** | | |
| Study Characteristics | Study Purpose | To explore differences in caregiver task difficulty, depressive symptoms, and life changes based on caregiver characteristics of gender, race (non-African American vs. African American), and type of relationship (spouse vs. non-spouse) to the person with stroke. |
|  | Source of Data | Primary quantitative data collected through a survey |
|  | Study Type | Cross-sectional |
|  | Data Collection Geography | Multiple sites across multiple states |
|  | Population | Caregivers |
|  | Sample Size | 243 caregivers |
|  | Medical Condition | Stroke |
| Findings: Categories of PCBEOs | Categories of PCBEOs Examined | Intangible burdens |
|  | Intangible Burdens | Family/social life impacts, stress and anxiety directly caused by out-of-pocket costs, financial debt, or lost income (i.e., financial toxicity) |
| Findings: Differences in PCBEOs | Types of Differences Examined | Race/ethnicity, sex/gender |
|  | Race/Ethnicity | Non–African American caregivers were relatively 3.1 (95% confidence interval [CI] [1.7, 6.3], *p*<0.001) times more likely to experience negative change related to having time for family activities. |
|  | Sex | Female caregivers had higher odds of difficulty managing finances (odds ratio=2.0, 95% CI [1.0, 4.1], *p*=0.041). |
| Findings: Strategies for Addressing Differences | Strategies for Addressing Differences | No |
|  | Methods/Instruments | 15-item Oberst Caregiving Burden Scale and 15-item Bakas Caregiving Outcomes Scale: Validated |
| **Kaddas, H. K., Millar, M. M., Herget, K. A., Carter, M. E., Ofori-Atta, B. S., Edwards, S. L., Codden, R. R., Sweeney, C., & Kirchhoff, A. C. (2024). Material financial hardship and insurance-related experiences among Utah’s rural and urban cancer survivors. *Journal of Cancer Survivorship*, 1–8.** | | |
| Study Characteristics | Study Purpose | To describe material financial hardship (e.g., using savings, credit card debt), insurance, and access to care experienced by Utah cancer survivors; investigate urban–rural differences in financial hardship. |
|  | Source of Data | Primary quantitative data collected through a survey |
|  | Study Type | Cross-sectional |
|  | Data Collection Geography | Multiple sites in a single state |
|  | Population | Patients |
|  | Sample Size | 1,793 patients |
|  | Medical Condition | Cancer (all types) |
| Findings: Categories of PCBEOs | Categories of PCBEOs Examined | Direct medical costs  Direct non-medical costs  Intangible burdens |
|  | Direct Medical Costs | Medical financial debt |
|  | Direct Non-Medical Costs | Filed for bankruptcy |
|  | Intangible Burdens | Delaying or forgoing medical treatments due to costs, diminished household wealth, food insecurity, housing insecurity, hindered career or education advancement, reduced spending on basic goods, groceries, and leisure activities |
| Findings: Differences in PCBEOs | Types of Differences Examined | Geographic location |
|  | Geographic Location | Hispanic participants who lived rurally had lower odds (odds ratio [OR]=0.24, 95% confidence interval [CI] [0.08, 0.73]) of financial hardship than their urban counterparts. Rural survivors who received chemotherapy were over 2 times (OR=2.72, 95% CI [1.08, 6.86]) more likely to report a financial hardship compared with urban survivors who received chemotherapy; there were no urban–rural differences among survivors who received other treatment regimens. |
| Findings: Strategies for Addressing Differences | Strategies for Addressing Differences | No |
|  | Methods/Instruments | The survey was self-designed and borrowed financial hardship questions from the Behavioral Risk Factor Surveillance System questionnaires and other surveys. Whether the method/instrument is validated and/or publicly available was not mentioned. |
| **Kahn, L. S., Vest, B. M., Madurai, N., Singh, R., York, T. R., Cipparone, C. W., Reilly, S., Malik, K. S., & Fox, C. H. (2015). Chronic kidney disease (CKD) treatment burden among low-income primary care patients. *Chronic Illness*, *11*(3), 171–183.** | | |
| Study Characteristics | Study Purpose | To explore the self-management strategies of low-income primary care patients with stage 3 and 4 chronic kidney disease (CKD) in the United States. |
|  | Source of Data | Qualitative interviews |
|  | Study Type | Qualitative study |
|  | Data Collection Geography | Multiple sites in a single state |
|  | Population | Patients |
|  | Sample Size | 34 patients |
|  | Medical Condition | CKD |
| Findings: Categories of PCBEOs | Categories of PCBEOs Examined | Indirect impacts  Intangible burdens |
|  | Indirect Costs | Time spent traveling to or between medical appointments, unpaid time off work, early retirement |
|  | Intangible Burdens | Delaying or forgoing medical treatments due to costs, changes in employment status and/or insurance coverage |
| Findings: Differences in PCBEOs | Types of Differences Examined | Employment status, insurance |
|  | Employment Status | Taking off time from work and lost income were themes among employed patients. They described the amount of time it takes to take public transportation to get to a doctor’s office. Patients also mentioned that if they began a job, they were no longer eligible for Medicaid, and they had difficulties purchasing medications. |
|  | Insurance | Patients explained that when they were unemployed and on Medicaid, they had few problems filling their prescriptions. However, if they began a job with limited insurance and were no longer eligible for Medicaid, they encountered difficulties purchasing their prescriptions. |
| Findings: Strategies for Addressing Differences | Strategies for Addressing Differences | No |
|  | Methods/Instruments | No |
| **Kazzi, B., Chino, F., Kazzi, B., Jain, B., Tian, S., Paguio, J. A., Yao, J. S., Muralidhar, V., Mahal, B. A., Nguyen, P. L., Sanford, N. N., & Dee, E. C. (2022). Shared burden: The association between cancer diagnosis, financial toxicity, and healthcare cost-related coping mechanisms by family members of non-elderly patients in the USA. *Supportive Care in Cancer*, *30*(11), 8905–8917.** | | |
| Study Characteristics | Study Purpose | To assess the association between a cancer diagnosis and the health care cost-related coping mechanisms of participant family members through their decision to forgo or delay seeking medical care, one of the manifestations of financial toxicity. |
|  | Source of Data | Primary quantitative data collected through a survey |
|  | Study Type | Cross-sectional |
|  | Data Collection Geography | National |
|  | Population | Patients |
|  | Sample Size | 254,486 patients |
|  | Medical Condition | Cancer (all types) |
| Findings: Categories of PCBEOs | Categories of PCBEOs Examined | Intangible burdens |
|  | Intangible Burdens | Delaying or forgoing medical treatments due to costs |
| Findings: Differences in PCBEOs | Types of Differences Examined | Age, income, insurance, relationship status, sex/gender |
|  | Age | There was a statistically significant cancer diagnosis*age interaction term such that the association between cancer diagnosis and delaying care was stronger for participants aged 18–45 (18–45 with cancer: 24.9%; without cancer 16.9%; adjusted odds ratio [aOR]=1.24, 95% confidence interval [CI] [1.12–1.36], *p*<0.001) than those aged 46–64 (46–64 with cancer: 17.5%; without cancer: 15.2%; aOR=1.11, 95% CI [1.03–1.20], *p*=0.007) (interaction= 0.028). |
|  | Income | On multivariable logistic regression analysis, participants were significantly more likely to report family members delaying care if participants had a lower household income (participants whose family income to poverty threshold ratio was less than 1.0). |
|  | Insurance | On multivariable logistic regression analysis, participants were significantly more likely to report family members delaying care if participants lacked health insurance coverage (vs. participants who did not lack health insurance coverage: 37.2% vs. 12.3%, aOR=3.61, 95% CI [3.49–3.74], *p*<0.001). |
|  | Relationship Status | On multivariable logistic regression analysis, participants were significantly more likely to report family members delaying care if participants were unmarried (widowed, divorced, separated, and never married vs. married: 21.4% vs. 14.0%, aOR=1.22, 95% CI [1.18–1.26], *p*<0.001). |
|  | Sex | On multivariable logistic regression analysis, participants were significantly more likely to report family members delaying care if participants were female (female vs. male: 7.1% vs. 15.8%, aOR=1.04, 95% CI [1.01–1.07], *p*=0.004). |
| Findings: Strategies for Addressing Differences | Strategies for Addressing Differences | No |
|  | Methods/Instruments | No |
| **Kelley, A. S., McGarry, K., Gorges, R., & Skinner, J. S. (2015). The burden of health care costs for patients with dementia in the last 5 years of life. *Annals of Internal Medicine*, *163*(10), 729–736.** | | |
| Study Characteristics | Study Purpose | To examine social costs and financial risks faced by Medicare beneficiaries 5 years before death. |
|  | Source of Data | Secondary analysis of pre-existing survey data |
|  | Study Type | Retrospective cohort: Longitudinal study using pre-existing data on two groups to examine the relationship between an exposure and an outcome |
|  | Data Collection Geography | National |
|  | Population | Patients |
|  | Sample Size | 1,702 patients (proxy after death) |
|  | Medical Condition | Dementia, end-of-life patients, general |
| Findings: Categories of PCBEOs | Categories of PCBEOs Examined | Direct medical costs  Indirect impacts |
|  | Direct Medical Costs | Other direct medical costs: Composite medical out-of-pocket costs |
|  | Indirect Costs | Time spent providing unpaid caregiving |
| Findings: Differences in PCBEOs | Types of Differences Examined | Race/ethnicity |
|  | Race/Ethnicity | Black decedents at high risk for dementia had much lower levels of out-of-pocket spending ($23,425) than non-Black decedents ($64,819), most likely because of fewer financial resources. However, more informal care was provided in Black households ($117,496) than in non-Black households ($80,038) (*p*<0.001), so the combined value of out-of-pocket spending and informal care was nearly identical between the two groups. |
| Findings: Strategies for Addressing Differences | Strategies for Addressing Differences | No |
|  | Methods/Instruments | Health and Retirement Study |
| **Lee, K., Yefimova, M., Puga, F., & Pickering, C. E. (2021). Gender differences in caregiver burden among family caregivers of persons with dementia. *Journal of Gerontological Nursing*, *47*(7), 33–42.** | | |
| Study Characteristics | Study Purpose | To identify gender-dependent factors that influence caregiver burden among family caregivers of people with dementia through secondary data analysis. |
|  | Source of Data | Secondary analysis of pre-existing survey data |
|  | Study Type | Cross-sectional |
|  | Data Collection Geography | Not stated/unclear |
|  | Population | Caregivers |
|  | Sample Size | 379 caregivers |
|  | Medical Condition | Dementia |
| Findings: Categories of PCBEOs | Categories of PCBEOs Examined | Direct non-medical costs  Indirect impacts |
|  | Direct Non-Medical Costs | Other direct non-medical costs: General descriptions of financial burden |
|  | Indirect Costs | Time spent providing unpaid caregiving |
| Findings: Differences in PCBEOs | Types of Differences Examined | Sex/gender |
|  | Sex | On average, male caregivers reported a slightly higher financial burden than females (mean=2.43 and 2.14, respectively; *p*=0.027). |
| Findings: Strategies for Addressing Differences | Strategies for Addressing Differences | No |
|  | Methods/Instruments | No |
| **Lee, M. K., Durani, U., Zhang, N., Hilal, T., Warsame, R. M., Borah, B., Khera, N., & Griffin, J. M. (2023). Relationships among health insurance literacy, financial toxicity, and sociodemographic factors in patients with cancer. *JCO Oncology Practice*, *19*(10), 888–898** | | |
| Study Characteristics | Study Purpose | To describe the prevalence of health insurance literacy (HIL) and investigate how patient-reported outcome measures assessing HIL are related to financial toxicity (FT) in patients with cancer. |
|  | Source of Data | Primary quantitative data collected through a survey |
|  | Study Type | Cross-sectional |
|  | Data Collection Geography | Single institution/site |
|  | Population | Patients |
|  | Sample Size | 406 patients |
|  | Medical Condition | Cancer (all types) |
| Findings: Categories of PCBEOs | Categories of PCBEOs Examined | Intangible burdens |
|  | Intangible Burdens | Stress and anxiety directly caused by out-of-pocket costs, financial debt, or lost income (i.e., FT)  Other intangible burdens: Financial stress |
| Findings: Differences in PCBEOs | Types of Differences Examined | Age, income |
|  | Age | Older age was significantly associated with less FT (estimate=-0.23, *p*<0.001). |
|  | Income | Higher income was significantly associated with less FT (estimate=0.27, *p*<0.001). |
| Findings: Strategies for Addressing Differences | Strategies for Addressing Differences | No |
|  | Methods/Instruments | COST: A FACIT Measure of Financial Toxicity (FACIT-COST)  Kaiser Family Foundation Health Insurance Study. Whether the method/instrument is validated and/or publicly available was not mentioned. |
| **Li, C., Narcisse, M. R., & McElfish, P. A. (2020). Medical financial hardship reported by Native Hawaiian and Pacific Islander cancer survivors compared with non‐Hispanic whites. *Cancer*, *126*(12), 2900–2914.** | | |
| Study Characteristics | Study Purpose | To describe the medical financial hardship (MFH) experienced by Native Hawaiian and Pacific Islander (NHPI) cancer survivors using the 2014 NHPI-National Health Interview Survey (NHIS) and compare it with the MFH experienced by adult (non-Hispanic white) NHW cancer survivors identified from the general NHIS. |
|  | Source of Data | Secondary analysis of pre-existing survey data |
|  | Study Type | Cross-sectional |
|  | Data Collection Geography | National |
|  | Population | Patients |
|  | Sample Size | 2,248 patients |
|  | Medical Condition | Cancer (all types) |
| Findings: Categories of PCBEOs | Categories of PCBEOs Examined | Direct medical costs  Intangible burdens |
|  | Direct Medical Costs | Medical financial debt |
|  | Intangible Burdens | Delaying or forgoing medical treatments due to costs, stress and anxiety directly caused by out-of-pocket costs, financial debt, or lost income (i.e., financial toxicity) |
| Findings: Differences in PCBEOs | Types of Differences Examined | Education, race/ethnicity, sex/gender |
|  | Educational Attainment | NHPIs with a college degree or higher were more likely to report MFH (adjusted odds ratio [aOR]=8.34, 95% confidence interval [CI] [1.83, 38.07], *p*=0.008), material hardship (aOR=5.37, 95% CI [1.43, 20.24], *p*=0.015), and psychological hardship (aOR=6.18, 95% CI [1.35, 28.20], *p*=0.021) compared with NHPIs without a college degree. |
|  | Race/Ethnicity | NHPIs were slightly more likely to report behavioral MFH (24% vs. 19%; *p*=0.090) than NHW cancer survivors.  Among cancer survivors aged 65 or older, NHPIs were more likely to report psychological hardship (36% vs. 22%, *p*=0.040), behavioral hardship (27% vs. 9%, *p*=.004), and any MFH (59% vs. 32%, *p*=<0.01).  Among survivors younger than 65, the proportions of cancer survivors reporting any MFH were lower in NHPIs compared with NHWs (46% vs. 65%, *p*=0.034).  Among NHPI cancer survivors, those who reported NHPI as their only race were significantly more likely to report MFH in all three domains than those who reported NHPI and other races (aOR=22.50, 95% CI [3.49, 144.98], *p*=0.002). |
|  | Sex | NHPI women (aOR=7.48; 95% CI [2.80, 20.01], *p*<0.001) were significantly more likely to report MFH and psychological hardship (aOR=3.80, 95% CI [1.49, 9.69], *p*=0.007) compared with NHPI men. |
| Findings: Strategies for Addressing Differences | Strategies for Addressing Differences | No |
|  | Methods/Instruments | Subset of NHIS questions |
| **Liang, I., Tay, D. L., Kirchhoff, A. C., Schwanke, G., Ellington, L., Pisu, M., & Mooney, K. (2024). Financial toxicity of total cancer care immunotherapy patients and caregivers: Impacts of COVID-19 pandemic and inflation. *Supportive Care in Cancer*, *32*(12), 790.** | | |
| Study Characteristics | Study Purpose | To explore the sources, buffers, and consequences of financial toxicity (FT) among patients with advanced cancers receiving immunotherapies and those caring for them, recognizing the pervasive impact of economic inflation. |
|  | Source of Data | Primary quantitative data collected through a survey  Qualitative interviews |
|  | Study Type | Cross-sectional |
|  | Data Collection Geography | Single institution/site |
|  | Population | Both |
|  | Sample Size | 16 patients and 10 caregivers |
|  | Medical Condition | Lung cancer, head and neck cancer |
| Findings: Categories of PCBEOs | Categories of PCBEOs Examined | Intangible burdens |
|  | Intangible Burdens | Stress and anxiety directly caused by out-of-pocket costs, financial debt, or lost income (i.e., FT) |
| Findings: Differences in PCBEOs | Types of Differences Examined | Age, geographic location |
|  | Age | Compared with metropolitan-dwelling patients, greater proportions of non-metropolitan patients (n=4, 44% vs. n=2, 29%) had high levels of FT. |
|  | Geographic Location | Compared with metropolitan dwelling patients, greater proportions of non-metropolitan patients (n=4, 44% vs. n=2, 29%) had high levels of FT. |
| Findings: Strategies for Addressing Differences | Strategies for Addressing Differences | Addressing communication challenges between patients and their care team is important because patients and providers may have difficulty communicating openly about cost concerns.  Future interventions should assess the applicability of universal versus targeted screening tools for FT, a process to assess FT proactively and periodically, and improvement of training on cost communication in the care team.  Simplifying and standardizing access to essential financial resources and programs could help mitigate disparities in access to supports. |
|  | Methods/Instruments | COST tool: Publicly available, validated |
| **Liang, M. I., Pisu, M., Summerlin, S. S., Boitano, T. K., Blanchard, C. T., Bhatia, S., & Huh, W. K. (2020). Extensive financial hardship among gynecologic cancer patients starting a new line of therapy. *Gynecologic Oncology*, *156*(2), 271–277.** | | |
| Study Characteristics | Study Purpose | To evaluate the three domains of financial hardship (psychological response, material conditions, and coping behaviors) among gynecologic cancer patients receiving treatment. |
|  | Source of Data | Primary quantitative data collected through a survey |
|  | Study Type | Cross-sectional |
|  | Data Collection Geography | Single institution/site |
|  | Population | Patients |
|  | Sample Size | 121 patients |
|  | Medical Condition | Gynecologic cancer |
| Findings: Categories of PCBEOs | Categories of PCBEOs Examined | Direct medical costs  Direct non-medical costs  Indirect impacts  Intangible burdens |
|  | Direct Medical Costs | Medical Financial Debt |
|  | Direct Non-Medical Costs | Filed for bankruptcy |
|  | Indirect Costs | Unpaid time off work, early retirement |
|  | Intangible Burdens | Fear and anxiety about future ability to afford medical treatments; changes in employment status and/or insurance coverage; food insecurity; housing insecurity; reduced spending on basic goods, groceries, and leisure activities; stress and anxiety directly caused by out-of-pocket costs, financial debt, or lost income (i.e., financial toxicity) |
| Findings: Differences in PCBEOs | Types of Differences Examined | Age, income |
|  | Age | Age younger than 65 (adjusted odds ratio [aOR]=2.61, 95% confidence interval [CI] [1.04, 6.52]) was associated with increased odds of screening positive for financial distress. |
|  | Income | Income less than $40,000 (aOR=3.41, 95% CI [1.28, 9.09]) was associated with increased odds of screening positive for financial distress. |
| Findings: Strategies for Addressing Differences | Strategies for Addressing Differences | Using thresholds (younger than 65 and income less than $40,000) to identify patients for earlier or more intensive interventions. |
|  | Methods/Instruments | Comprehensive Score for financial Toxicity tool: Publicly available, validated |
| **Massa, S. T., Osazuwa-Peters, N., Boakye, E. A., Walker, R. J., & Ward, G. M. (2019). Comparison of the financial burden of survivors of head and neck cancer with other cancer survivors. *JAMA Otolaryngology–Head & Neck Surgery*, *145*(3), 239–249.** | | |
| Study Characteristics | Study Purpose | To assess the financial burdens of head and neck cancers (HNCs) compared with other cancers. |
|  | Source of Data | Secondary analysis of pre-existing survey data |
|  | Study Type | Cross-sectional |
|  | Data Collection Geography | National |
|  | Population | Patients |
|  | Sample Size | 16,771 patients (10,083,586 weighted) |
|  | Medical Condition | Head and neck cancer compared with other cancers |
| Findings: Categories of PCBEOs | Categories of PCBEOs Examined | Direct medical costs |
|  | Direct Medical Costs | Out-of-pocket medical costs |
| Findings: Differences in PCBEOs | Types of Differences Examined | Geographic location, income, insurance |
|  | Geographic Location | Living in the South was associated with higher relative expenses (out-of-pocket costs as a percentage of income) compared with living in the Northeast (β=2.82, 95% confidence intervals [CI] [1.47, 5.43]). |
|  | Income | Higher income rating on the poverty index was associated with lower relative expenses (β=0.043, 95% CI [0.21, 0.85]). |
|  | Insurance | Public insurance was associated with higher relative expenses compared with private insurance (β=1.89, 95% CI [1.10, 3.28]). |
| Findings: Strategies for Addressing Differences | Strategies for Addressing Differences | No |
|  | Methods/Instruments | Used the Medical Expenditure Panel Survey to calculate relative out-of-pocket expenses defined as out-of-pocket expenses as a percentage of income: Publicly available |
| **McDougall, J. A., Banegas, M. P., Wiggins, C. L., Chiu, V. K., Rajput, A., & Kinney, A. Y. (2018). Rural disparities in treatment-related financial hardship and adherence to surveillance colonoscopy in diverse colorectal cancer survivors. *Cancer Epidemiology, Biomarkers & Prevention*, *27*(11), 1275–1282.** | | |
| Study Characteristics | Study Purpose | To investigate potential rural disparities in the likelihood of financial hardship and nonadherence to surveillance colonoscopy. |
|  | Source of Data | Primary quantitative data collected through a survey |
|  | Study Type | Cross-sectional |
|  | Data Collection Geography | Multiple sites in a single state |
|  | Population | Patients |
|  | Sample Size | 277 patients |
|  | Medical Condition | Colorectal cancer |
| Findings: Categories of PCBEOs | Categories of PCBEOs Examined | Direct medical costs  Direct non-medical costs  Intangible burdens |
|  | Direct Medical Costs | Medical financial debt |
|  | Direct Non-Medical Costs | Filed for bankruptcy |
|  | Intangible Burdens | Reduced spending on basic goods, groceries, and leisure activities |
| Findings: Differences in PCBEOs | Types of Differences Examined | Age, geographic location, relationship status, other (language) |
|  | Age | For every 1-year increase in the age of colorectal cancer diagnosis, a 9% decrease in the likelihood of financial hardship (odds ratio [OR]=0.91, 95% confidence interval [CI] [0.88, 0.95]) was observed. |
|  | Geographic Location | In the multivariable adjusted model, rural colorectal cancer survivors were nearly twice as likely (OR=1.86; 95% CI [1.06, 3.28]) as urban survivors to report treatment-related financial hardship. |
|  | Relationship Status | Those who were divorced, separated, or single (OR=1.94, 95% CI [1.06, 3.54]) were nearly twice as likely to report financial burden as married participants. |
|  | Other | Language: Spanish-speaking Hispanic individuals were over 3 times as likely to report financial burden as non-Hispanic White individuals (OR=3.09, 95% CI [1.39, 6.87]). |
| Findings: Strategies for Addressing Differences | Strategies for Addressing Differences | No |
|  | Methods/Instruments | Self-designed questionnaire borrowing questions from the Medical Expenditure Panel Survey Experiences with Cancer Supplement: Validated and publicly available |
| **McDougall, J. A., Blair, C. K., Wiggins, C. L., Goodwin, M. B., Chiu, V. K., Rajput, A., & Kinney, A. Y. (2019). Socioeconomic disparities in health-related quality of life among colorectal cancer survivors. *Journal of Cancer Survivorship*, *13*(3), 459–467.** | | |
| Study Characteristics | Study Purpose | To investigate the relationship between socioeconomic characteristics and health-related quality of life in a diverse group of colorectal cancer (CRC) survivors. |
|  | Source of Data | Primary quantitative data collected through a survey |
|  | Study Type | Cross-sectional |
|  | Data Collection Geography | Multiple sites in a single state |
|  | Population | Patients |
|  | Sample Size | 301 patients |
|  | Medical Condition | Colorectal cancer |
| Findings: Categories of PCBEOs | Categories of PCBEOs Examined | Intangible burdens |
|  | Intangible Burdens | Quality-of-life impacts (not quality-adjusted life-years or disability-adjusted life-years) |
| Findings: Differences in PCBEOs | Types of Differences Examined | Income, insurance |
|  | Income | Low-income ($30,000 or less) CRC survivors had on average, a 4.70-point (95% confidence interval [CI] [1.10, 8.28]) higher pain interference score and a 5.13-point (95% CI [-8.56, -1.71]) lower physical function score, compared with CRC survivors with an income of $70,000 or more. |
|  | Insurance | CRC survivors with Medicaid insurance had pain interference scores that were 7.59 points (95% CI [3.18, 12.0]) higher on average than those with private insurance. In addition, physical function scores for Medicaid-insured survivors were 6.94 points (95% CI [-10.6, -3.18]) lower than privately insured survivors. |
| Findings: Strategies for Addressing Differences | Strategies for Addressing Differences | Developing and adapting existing effective interventions to improve chronic illness self-management among low-income and low–health literacy populations |
|  | Methods/Instruments | PROMIS Short Forms v2.0 |
| **McKenna, R. M., Pintor, J. K., & Ali, M. M. (2019). Insurance-based disparities in access, utilization, and financial strain for adults with psychological distress. *Health Affairs*, *38*(5), 826–834.** | | |
| Study Characteristics | Study Purpose | To identify barriers to general and mental health care access and utilization for people with any psychological distress by insurance type in the post–Affordable Care Act era. |
|  | Source of Data | Secondary analysis of pre-existing survey data |
|  | Study Type | Cross-sectional |
|  | Data Collection Geography | National |
|  | Population | Patients |
|  | Sample Size | 63,177 patients (14,005 with psychological distress) |
|  | Medical Condition | Other: Psychological distress |
| Findings: Categories of PCBEOs | Categories of PCBEOs Examined | Intangible burdens |
|  | Intangible Burdens | Delaying or forgoing medical treatments due to costs |
| Findings: Differences in PCBEOs | Types of Differences Examined | Insurance |
|  | Insurance | Non-elderly adults with moderate or severe psychological distress with employer-sponsored insurance (odds ratio [OR]=1.559, *p*<0.001) and marketplace insurance (OR=3.963, *p*<0.001) had higher odds of not getting medical care due to cost compared with those with Medicaid.  Non-elderly adults with moderate or severe psychological distress with marketplace insurance also had higher odds of not getting mental health care (OR=1.746, *p*<0.01), specialty care (OR=2.301, *p*<0.001), and prescription medication (OR=2.272, *p*<0.001) due to cost compared with those with Medicaid. |
| Findings: Strategies for Addressing Differences | Strategies for Addressing Differences | Development of a plan quality and network breadth metric, akin to Medicare’s Star Rating System to increase transparency in plans.  Simplify administrative processes to increase provider participation in insurance plans.  State-level policy to address provider participation. |
|  | Methods/Instruments | National Health Interview Survey 2014–2017: Publicly available, validated |
| **Miller, J. E., Nugent, C. N., & Russell, L. B. (2015). Risk factors for family time burdens providing and arranging health care for children with special health care needs: Lessons from nonproportional odds models. *Social Science Research*, *52*, 602–614.** | | |
| Study Characteristics | Study Purpose | To identify need, enabling, and predisposing factors for high family time burdens associated with the health care of chronically ill children. |
|  | Source of Data | Secondary analysis of pre-existing survey data |
|  | Study Type | Cross-sectional |
|  | Data Collection Geography | National |
|  | Population | Caregivers |
|  | Sample Size | 37,860 caregivers |
|  | Medical Condition | Other: Children with special health care needs |
| Findings: Categories of PCBEOs | Categories of PCBEOs Examined | Indirect impacts |
|  | Indirect Costs | Time to attend medical appointments, time spent providing unpaid caregiving |
| Findings: Differences in PCBEOs | Types of Differences Examined | Education, geographic location, income, insurance, race/ethnicity |
|  | Educational Attainment | Households with the most educated adult having high school or less had higher odds of 6 or more (adjusted odds ratio [aOR]=1.21, *p*<0.001) and 11 or more (aOR=1.36, *p*<0.001) hours of providing health care at home; however, they had lower odds of providing 1 or more hours of health care at home (aOR=0.86, *p*<0.05).  Households with the most educated adult having high school or less also had higher odds of 6 or more hours of arranging or coordinating care (aOR=1.40, *p*<0.001), providing moderate or high care (aOR=1.31, *p*<0.001), and providing high care (aOR=1.72, *p*<0.001). |
|  | Geographic Location | Rural residence was associated with higher odds of providing 6 or more hours of health care at home (aOR=1.16, *p*<0.01) and moderate to high caregiving (aOR=1.11, *p*<0.05). |
|  | Income | Compared with family income >400% of the federal poverty level (FPL), families with 133% or less of FPL had higher odds of 6 or more hours of health care at home (aOR=1.18, *p*<0.05), 6 or more hours of arranging/coordinating care (aOR=1.78, *p*<0.001). |
|  | Insurance | Compared with private insurance, public insurance and public and private insurance are associated with more time caregiving across all categories (*p*<0.001).  The largest increase in odds is between 11 or more and fewer than 11 hours of providing health care at home, with those with public insurance reporting 2.03 times the odds of providing 11 or more hours of care.  Additionally, those with gaps in insurance coverage during the year had higher odds of providing 6 or more hours of health care at home compared with those with the same insurance status all year (aOR=1.24, *p*<0.05). |
|  | Race/Ethnicity | Compared with non-Hispanic White, non-Hispanic Black race had higher odds of 6 or more hours of arranging or coordinating care (aOR=1.26, *p*<0.001) and moderate to high caregiving (aOR=1.17, *p*<0.05).  Compared with non-Hispanic White, other non-Hispanic race had higher odds of 6 or more hours of health care at home (aOR=1.21, *p*<0.05) and moderate to high caregiving (aOR=1.17, *p*<0.05).  Compared with non-Hispanic White, Hispanic was associated with higher odds of 6 or more hours of health care at home (aOR=1.34, *p*<0.01), 11 or more hours of health care at home (aOR=1.60, *p*<0.001), 6 or more hours of arranging or coordinating care (aOR=1.19, *p*<0.05), and moderate to high caregiving (aOR=1.22, *p*<0.01). |
| Findings: Strategies for Addressing Differences | Strategies for Addressing Differences | No |
|  | Methods/Instruments | National Survey of Children with Special Health Care Needs |
| **Moon, H. E., Haley, W. E., Rote, S. M., & Sears, J. S. (2020). Caregiver well-being and burden: Variations by race/ethnicity and care recipient nativity status. *Innovation in Aging*, *4*(6).** | | |
| Study Characteristics | Study Purpose | To examine the differences in caregiver outcomes (care burden, psychological well-being, and self-rated health) by care recipient nativity status (U.S. born vs. foreign born) and the extent to which caregiver outcomes vary by care recipient nativity status and caregiver race/ethnicity (non-Hispanic White, non-Hispanic Black, Hispanic, and others). |
|  | Source of Data | Secondary analysis of pre-existing survey data |
|  | Study Type | Cross-sectional |
|  | Data Collection Geography | National |
|  | Population | Caregivers |
|  | Sample Size | 1,436 caregivers |
|  | Medical Condition | Older adults, general |
| Findings: Categories of PCBEOs | Categories of PCBEOs Examined | Indirect impacts  Intangible burdens |
|  | Indirect Costs | Time spent providing unpaid caregiving, unpaid time off work, early retirement, other indirect impacts: Help with activities of daily living (ADLs) |
|  | Intangible Burdens | Family/social life impacts, stress and anxiety directly caused by out-of-pocket costs, financial debt, or lost income (i.e., financial toxicity) |
| Findings: Differences in PCBEOs | Types of Differences Examined | Education, other (immigration status) |
|  | Educational Attainment | Some college or more was associated with a decrease in caregiver burden (-0.07, *p*<0.05). |
|  | Other | Immigration: Caregivers of foreign-born care recipients reported higher stressors, including help with ADLs and instrumental ADLs (*t*=3.89, *p*<0.01), help with medical care (*t*=5.71, *p*<0.001), and help with medical insurance and appointments (*t*=2.05, *p*<0.05).  Caregivers of foreign-born care recipients had higher limited activities due to care and higher financial difficulties due to caregiving (χ2=30.33; *p*<0.001). |
| Findings: Strategies for Addressing Differences | Strategies for Addressing Differences | No |
|  | Methods/Instruments | Uses National Health and Aging Trends Study National Study of Caregiving data: Publicly available |
| **Mukherjee, K., & Kamal, K. M. (2017). Sociodemographic determinants of out-of-pocket expenditures for patients using prescription drugs for rheumatoid arthritis. *American Health & Drug Benefits*, *10*(1), 7.** | | |
| Study Characteristics | Study Purpose | To explore the relationship between sociodemographic characteristics, personal characteristics, and out-of-pocket expenditures associated with rheumatoid arthritis prescription medications. |
|  | Source of Data | Secondary analysis of pre-existing survey data |
|  | Study Type | Cross-sectional |
|  | Data Collection Geography | National |
|  | Population | Patients |
|  | Sample Size | 1,090 patients |
|  | Medical Condition | Arthritis |
| Findings: Categories of PCBEOs | Categories of PCBEOs Examined | Direct medical costs |
|  | Direct Medical Costs | Out-of-pocket medical costs |
| Findings: Differences in PCBEOs | Types of Differences Examined | Age, income, insurance, sex/gender |
|  | Age | The mean annual out-of-pocket expenditure decreased with age (0.98, *p*<0.0001, 95% confidence interval [CI] [0.97, 0.99]). |
|  | Income | The out-of-pocket expenditures were lower for poor (0.60, *p*=0.003, 95% CI [0.43, 0.84]) and low-income (0.69, *p*=0.034, 95% CI [0.49, 0.97]) patients versus high-income patients. |
|  | Insurance | The out-of-pocket expenditures were lower for privately insured (0.31, *p*<0.0001, 95% CI [0.21, 0.45]) patients and publicly insured (0.18, *p*<0.0001, 95% CI [0.12, 0.27]) patients versus uninsured patients |
|  | Sex | The mean annual out-of-pocket expenditure was lower (0.73, *p*=0.006, 95% CI [0.58, 0.92]) for male patients than for female patients. |
| Findings: Strategies for Addressing Differences | Strategies for Addressing Differences | No |
|  | Methods/Instruments | Medical Expenditure Panel Survey data from 2009 to 2012 |
| **Ng, A. P., Sanaiha, Y., Verma, A., Lee, C., Akhavan, A., Cohen, J. G., & Benharash, P. (2022). Insurance-based disparities and risk of financial toxicity among patients undergoing gynecologic cancer operations. *Gynecologic Oncology*, *166*(2), 200–206.** | | |
| Study Characteristics | Study Purpose | To evaluate the risk of financial toxicity (FT) among inpatients undergoing gynecologic cancer resections and the association of insurance status with clinical and financial outcomes. |
|  | Source of Data | Secondary analysis of pre-existing survey data |
|  | Study Type | Cross-sectional |
|  | Data Collection Geography | National |
|  | Population | Patients |
|  | Sample Size | 462,529 patients |
|  | Medical Condition | Gynecologic cancer |
| Findings: Categories of PCBEOs | Categories of PCBEOs Examined | Direct medical costs  Intangible burdens |
|  | Direct Medical Costs | Out-of-pocket medical costs |
|  | Intangible Burdens | Stress and anxiety directly caused by out-of-pocket costs, financial debt, or lost income (i.e., FT) |
| Findings: Differences in PCBEOs | Types of Differences Examined | Insurance, race/ethnicity |
|  | Insurance | After risk adjustment, hospitalization costs significantly increased among the uninsured cohort over the 12-year study period from an estimated $17,500 (95% confidence interval [CI] [$16,300, $18,700] to $21,600 [$20,400, $22,700]), while the insured cohort followed a similar increase.  An estimated 52.8% of uninsured and 15.4% of insured patients were at risk of FT.  Over the study period, the estimated risk of FT significantly increased from 40.5% (95% CI: 34.1–47.2) to 64.3% (57.7–70.4) among uninsured patients and remained stable among Insured patients despite rising costs in both cohorts over time.  Among the insured, patients with public insurance (Medicare: 1.12 [1.06–1.18], *p*<0.001; Medicaid: 1.21 [1.14–1.30], *p*<0.001, ref.: Private) had higher odds of FT. |
|  | Race/Ethnicity | Among the insured, Black (adjusted odds ratio=1.27, 95% CI [1.19, 1.35], *p*<0.001) and Hispanic (1.08, [1.01, 1.16], *p*=0.03; ref.: White) had higher odds of FT. |
| Findings: Strategies for Addressing Differences | Strategies for Addressing Differences | No |
|  | Methods/Instruments | No |
| **Novak, P., Anderson, A. C., & Chen, J. (2018). Changes in health insurance coverage and barriers to health care access among individuals with serious psychological distress following the Affordable Care Act. *Administration and Policy in Mental Health and Mental Health Services Research*, *45*(6), 924–932.** | | |
| Study Characteristics | Study Purpose | To examine the impact of the implementation of the Affordable Care Act on the rate of health insurance coverage and reported barriers to health care access among individuals with serious psychological distress. |
|  | Source of Data | Secondary analysis of pre-existing survey data |
|  | Study Type | Cross-sectional |
|  | Data Collection Geography | National |
|  | Population | Patients |
|  | Sample Size | 6,052 |
|  | Medical Condition | Other: Psychological distress |
| Findings: Categories of PCBEOs | Categories of PCBEOs Examined | Intangible burdens |
|  | Intangible Burdens | Delaying or forgoing medical treatments due to costs |
| Findings: Differences in PCBEOs | Types of Differences Examined | Income, race/ethnicity |
|  | Income | Individuals with family income above 200% federal poverty level (FPL) were less likely to report not affording health care (average marginal effects=-0.05, *p*=0.01), compared with those with income below the 100% FPL. |
|  | Race/Ethnicity | Black (average marginal effects=-0.06, *p*<0.001) and other race (average marginal effects=-0.08, *p*=0.01) individuals were less likely to report not affording health care, compared with White individuals. |
| Findings: Strategies for Addressing Differences | Strategies for Addressing Differences | No |
|  | Methods/Instruments | National Health Interview Survey: Publicly available and validated |
| **O'Neill, K. M., Jean, R. A., Gross, C. P., Becher, R. D., Khera, R., Elizondo, J. V., & Nasir, K. (2020). Financial hardship after traumatic injury: Risk factors and drivers of out-of-pocket health expenses. *Journal of Surgical Research*, *256*, 1–12.** | | |
| Study Characteristics | Study Purpose | To investigate whether patients with a traumatic injury experienced financial hardship from out-of-pocket (out-of-pocket) health expenses. |
|  | Source of Data | Secondary analysis of pre-existing survey data |
|  | Study Type | Cross-sectional |
|  | Data Collection Geography | National |
|  | Population | Both |
|  | Sample Size | 90,964 families |
|  | Medical Condition | Other: Injury and health care utilization |
| Findings: Categories of PCBEOs | Categories of PCBEOs Examined | Direct medical costs |
|  | Direct Medical Costs | Out-of-pocket medical costs |
| Findings: Differences in PCBEOs | Types of Differences Examined | Age, geographic location, income, insurance, race/ethnicity, sex/gender |
|  | Age | Higher family age was associated with higher odds of excess financial burden (>20% of income on out-of-pocket expenses) in the unadjusted (odds ratio [OR]=1.01, 95% confidence interval [CI] [1.01, 1.02]) and adjusted model (adjusted OR [aOR]=1.01, 95% CI [1.01, 1.02]).  Higher family age was also associated with higher odds of catastrophic medical expenses (>40% of income on out-of-pocket expenses) in the unadjusted (OR=1.01, 95% CI [1.00, 1.01]) and adjusted model (aOR=1.01, 95% CI [1.00, 1.01]). |
|  | Geographic Location | Compared with the Northeast, families in the South had higher odds of excess financial burden in the unadjusted (OR=1.27, 95% CI [1.11, 1.45]) and adjusted (aOR=1.18, 95% CI [1.03, 1.36]) model, as well as higher odds of catastrophic medical expenses in the unadjusted model. |
|  | Income | Compared with middle income, very low-income (OR=14.79, 95% CI [12.6, 17.4]; aOR=18.1, 95% CI [15.3, 21.4]) and low-income (OR=2.97, 95% CI [2.45, 3.61]; aOR=2.97, 95% CI [2.45, 3.61]) families have higher odds of excess financial burden in the unadjusted model. |
|  | Insurance | Compared with private insurance, families with public insurance (OR=3.14, 95% CI [2.36, 3.47]; OR=3.85, 95% CI [3.39, 4.38]) and no insurance (OR=2.65, 95% CI [2.35, 2.98]; OR=3.56, 95% CI [3.06, 4.13]) had higher odds of excess financial burden and catastrophic medical expenses, respectively, in unadjusted models.  In adjusted models, public insurance was associated with lower odds of excess financial burden and catastrophic medical expenses, respectively (aOR=0.61, 95% CI [0.54, 0.69]; aOR=0.68, 95% CI [0.59, 0.79]). |
|  | Race/Ethnicity | Compared with White patients, Asian patients had lower odds of excess financial burden in the unadjusted model (OR=0.65, 95% CI [0.50, 0.83]), and Black (aOR=0.65, 95% CI [0.58, 0.74]), Hispanic (aOR=0.66, 95% CI [0.58, 0.75]), and multi-race (aOR=0.68, 95% CI [0.49, 0.95]) patients had lower odds of excess financial burden in the adjusted model.  Compared with White patients, Black (OR=1.41, 95% CI [1.22, 1.63]) and Hispanic (OR=1.20, 95% CI [1.06, 1.36]) patients had higher odds of catastrophic medical expenses in the unadjusted model; however, Black (aOR=0.78, 95% CI [0.68, 0.90]) and Hispanic (aOR=0.78, 95% CI [0.67, 0.92]) patients had lower odds of catastrophic medical expenses in the adjusted model. |
|  | Sex | Female sex was associated with higher odds of excess financial burden in the unadjusted (OR=1.83, 95% CI [1.68, 2.00]) and adjusted (aOR=1.13, 95% CI [1.02, 1.24]) models. Female sex was also associated with higher odds of catastrophic medical expenses in the unadjusted (aOR=1.96, 95% CI [1.76, 2.18]) and adjusted models (aOR=1.18, 95% CI [1.05, 1.33]). |
| Findings: Strategies for Addressing Differences | Strategies for Addressing Differences | No |
|  | Methods/Instruments | Uses Medical Expenditure Panel Survey data to calculate excess financial burden, defined as >20% of income on out-of-pocket expenses, and catastrophic medical expenses, defined as >40% of income on out-of-pocket expenses: Publicly available and validated |
| **Odai-Afotey, A., Lederman, R. I., Ko, N. Y., Gagnon, H., Fikre, T., Gundersen, D. A., Revette, A. C., Hershman, D. L., Crew, K. D., Keating, N. L., & Freedman, R. A. (2023). Breast cancer treatment receipt and the role of financial stress, health literacy, and numeracy among diverse breast cancer survivors. *Breast Cancer Research and Treatment*, *200*(1), 127–137.** | | |
| Study Characteristics | Study Purpose | To examine economic hardship, health literacy, and numeracy and whether these factors were associated with differences in receipt of recommended treatment among breast cancer survivors. |
|  | Source of Data | Primary quantitative data collected through a survey |
|  | Study Type | Cross-sectional |
|  | Data Collection Geography | Multiple sites across multiple states |
|  | Population | Patients |
|  | Sample Size | 296 patients |
|  | Medical Condition | Breast cancer |
| Findings: Categories of PCBEOs | Categories of PCBEOs Examined | Direct medical costs  Indirect impacts  Intangible burdens |
|  | Direct Medical Costs | Medical financial debt |
|  | Indirect Costs | Unpaid time off work, early retirement |
|  | Intangible Burdens | Changes in employment status and/or insurance coverage; reduced spending on basic goods, groceries, and leisure activities; stress and anxiety directly caused by out-of-pocket costs; financial debt; or lost income (i.e., financial toxicity) |
| Findings: Differences in PCBEOs | Types of Differences Examined | Race/ethnicity |
|  | Race/Ethnicity | Compared with non-Hispanic White patients, a higher percentage non-Hispanic Black and Hispanic patients reported borrowing money or going into debt (10.7% vs. 16.2% and 33.3%, *p*=0.001), making financial sacrifices (22.5% vs. 28.4% and 40.5%, *p*=0.05), worry about paying large medical bills (24.7% vs. 27.0% and 50.0%, *p*=0.005), and unable to cover share of visit costs (6.2% vs. 20.8% and 15.4%, *p*=0.002). |
| Findings: Strategies for Addressing Differences | Strategies for Addressing Differences | No |
|  | Methods/Instruments | Self-designed questionnaire based on Medical Expenditure Panel Survey questions: Whether the method/instrument is validated and/or publicly available was not mentioned. |
| **Pandrangi, V. C., Farrell, N. F., Mace, J. C., Detwiller, K. Y., Smith, T. L., & Geltzeiler, M. (2021). Perceived financial insecurity impacts healthcare decision‐making among patients with sinusitis. *The Laryngoscope*, *131*(11), 2403–2412.** | | |
| Study Characteristics | Study Purpose | To examine the impact of perceived financial insecurity on health care decision making and treatment compliance among sinusitis patients. |
|  | Source of Data | Secondary analysis of pre-existing survey data |
|  | Study Type | Cross-sectional |
|  | Data Collection Geography | National |
|  | Population | Patients |
|  | Sample Size | 3,159 patients |
|  | Medical Condition | Other: Sinusitis |
| Findings: Categories of PCBEOs | Categories of PCBEOs Examined | Intangible burdens |
|  | Intangible Burdens | Delaying or forgoing medical treatments due to costs, fear and anxiety about future ability to afford medical treatments |
| Findings: Differences in PCBEOs | Types of Differences Examined | Age, employment status, geographic location, income, insurance, sex/gender, other (socioeconomic status) |
|  | Age | Middle age, 31–45 (odds ratio [OR]=1.28, 95% confidence interval [CI] [1.276, 1.285]) and 46–65 (OR=1.12, 95% CI [1.120, 1.28]) had increased odds of cost-saving actions compared with 18- to 30-year-olds, although those older than 65 had lower odds (OR=0.90, 95% CI [0.899, 0.906]). |
|  | Employment Status | Unemployment, including no job last week and had job past 12 months (OR=1.44, 95% CI [1.660, 1.670]), no job past 12 months (OR=1.09, 95% CI [1.085, 1.091]), and never worked (OR=1.54, 95% CI [1.533, 1.551]) was also associated with increased odds of cost-saving actions compared with having a job last week, with the never employed group having the highest odds. |
|  | Geographic Location | Patients in the Midwest (OR=1.10, 95% CI [1.099, 1.106]), South (OR=1.39, 95% CI [1.384, 1.392]), and West (OR=1.61, 95% CI [1.602, 1.612]) had higher odds of cost-saving actions than those in the Northeast. |
|  | Income | Lower ratio of income to federal poverty, including 1–2 (OR=1.67, 95% CI [1.660, 1.670]) and <1 (OR=1.31, 95% CI [1.305, 1.315]), level had increased odds of cost-saving action. |
|  | Insurance | Lack of insurance had the second-highest odds of cost-saving actions among all variables (OR=5.13, 95% CI [5.107, 5.159]). Public or other insurance has lower odds of cost-saving actions (OR=0.97, 95% CI [0.970, 0.975]). |
|  | Sex | Female patients have higher odds of cost-saving actions (OR=1.11, 95% CI [1.106,1.110]). |
|  | Other | Socioeconomic status: Reporting at least one measure of financial insecurity was associated with the highest odds of at least one cost-saving health care action (OR=5.94, 95% CI [5.911, 5.970], *p*<0.001). |
| Findings: Strategies for Addressing Differences | Strategies for Addressing Differences | No |
|  | Methods/Instruments | Subset of National Health Interview Survey questions: Publicly available and validated |
| **Patel, M. R., Tolentino, D. A., Smith, A., & Heisler, M. (2023). Economic burden, financial stress, and cost-related coping among people with uncontrolled diabetes in the US. *Preventive Medicine Reports*, *34*, 102246.** | | |
| Study Characteristics | Study Purpose | To describe in depth the state of economic burden, financial stress, and coping among people with high A1c and some indication of diabetes being impacted by economic burden. |
|  | Source of Data | Secondary analysis of pre-existing survey data |
|  | Study Type | Cross-sectional |
|  | Data Collection Geography | Single institution/site |
|  | Population | Patients |
|  | Sample Size | 600 patients |
|  | Medical Condition | Other: People with high A1c |
| Findings: Categories of PCBEOs | Categories of PCBEOs Examined | Intangible burdens |
|  | Intangible Burdens | Stress and anxiety directly caused by out-of-pocket costs, financial debt, or lost income (i.e., financial toxicity) |
| Findings: Differences in PCBEOs | Types of Differences Examined | Age, education, income, insurance, race/ethnicity, sex/gender |
|  | Age | Financial stress was significantly higher for those older than 65 than those younger than 65 (*p*<0.001). |
|  | Educational Attainment | Financial stress was significantly higher among those who had some college education (*p*<0.001). |
|  | Income | Financial stress was significantly higher among those 201%–400% of the poverty level (*p*<0.001). |
|  | Insurance | Financial stress was significantly higher among those with government insurance than non-government insurance (*p*<0.001). |
|  | Race/Ethnicity | Financial stress was significantly highest among non-Hispanic White people (*p*<0.001). |
|  | Sex | Financial stress was significantly higher among men than women (*p*<0.001). |
| Findings: Strategies for Addressing Differences | Strategies for Addressing Differences | No |
|  | Methods/Instruments | Financial Management Behavior Scale (FMBS): Whether the method/instrument is validated and/or publicly available was not mentioned. |
| **Pereira, C., LaRoche, A., Arredondo, B., Pugh, E., Disbrow, E., Reekes, T. H., Brickell, E., Boettcher, A., & Sawyer, R. J. (2022). Evaluating racial disparities in healthcare system utilization and caregiver burden among older adults with dementia. *The Clinical Neuropsychologist*, *36*(2), 353–366.** | | |
| Study Characteristics | Study Purpose | To examine the performance of the Modified Caregiver Strain Index (MCSI) in a sample of Black and White caregivers of people living with dementia. |
|  | Source of Data | Primary quantitative data collected through a survey |
|  | Study Type | Cross-sectional |
|  | Data Collection Geography | Single institution/site |
|  | Population | Caregivers |
|  | Sample Size | 153 caregivers |
|  | Medical Condition | Dementia |
| Findings: Categories of PCBEOs | Categories of PCBEOs Examined | Indirect impacts  Intangible burdens |
|  | Indirect Costs | Time spent providing unpaid caregiving |
|  | Intangible Burdens | Stress and anxiety directly caused by out-of-pocket costs, financial debt, or lost income (i.e., financial toxicity) |
| Findings: Differences in PCBEOs | Types of Differences Examined | Race/ethnicity |
|  | Race/Ethnicity | Black caregivers endorsed “caregiving is a physical strain” to a greater degree than White caregivers (*p*=0.003). |
| Findings: Strategies for Addressing Differences | Strategies for Addressing Differences | No |
|  | Methods/Instruments | MCSI: Whether the method/instrument is validated and/or publicly available was not mentioned. |
| **Pereira-Osorio, C., Brickell, E., Lee, B., Arredondo, B., & Sawyer, R. J. (2024). Performance of the modified caregiver strain index in a sample of Black and White persons living with dementia and their caregivers. *The Gerontologist*, *64*(7).** | | |
| Study Characteristics | Study Purpose | To evaluate racial differences in health care utilization and caregiver burden in a culturally diverse population of older adults with dementia. |
|  | Source of Data | Primary quantitative data collected through a survey |
|  | Study Type | Cross-sectional |
|  | Data Collection Geography | Single institution/site |
|  | Population | Both |
|  | Sample Size | 155 patient–caregiver dyads |
|  | Medical Condition | Dementia |
| Findings: Categories of PCBEOs | Categories of PCBEOs Examined | Intangible burdens |
|  | Intangible Burdens | Family/social life impacts |
| Findings: Differences in PCBEOs | Types of Differences Examined | Race/ethnicity |
|  | Race/Ethnicity | Mann-Whitney U test results revealed higher impacts on social life for White caregivers than Black caregivers (Mann-Whitney U=1562.00, *p*=0.037). |
| Findings: Strategies for Addressing Differences | Strategies for Addressing Differences | No |
|  | Methods/Instruments | Short-form Zarit Burden Interview: Validated |
| **Pudrovska, T., & Anishkin, A. (2020). Personal mastery and the medical, financial, and physical cancer burden: Gender and race differences among older adults. *Journal of Aging and Health*, *32*(9), 1233–1243.** | | |
| Study Characteristics | Study Purpose | To explore how a new cancer diagnosis affects trajectories of personal mastery among non-Hispanic older Black and White adults. |
|  | Source of Data | Secondary analysis of pre-existing survey data |
|  | Study Type | Cross-sectional |
|  | Data Collection Geography | National |
|  | Population | Patients |
|  | Sample Size | 672 patients |
|  | Medical Condition | Cancer (all types) |
| Findings: Categories of PCBEOs | Categories of PCBEOs Examined | Direct medical costs  Intangible burdens |
|  | Direct Medical Costs | Out-of-pocket medical costs |
|  | Intangible Burdens | Hindered career or education advancement |
| Findings: Differences in PCBEOs | Types of Differences Examined | Race/ethnicity, sex/gender |
|  | Race/Ethnicity | Within-gender comparisons reveal that White men’s average treatment effect for the treated (ATT) is significantly greater than Black men’s ATT (ATT [White men] − ATT [Black men] = -0.098, *p*<0.05), whereas there is no significant race difference among women.  The negative effect of doctor visits on mastery was much stronger in White men (β=-0.118, *p*<0.001) than Black women (β=-0.081, *p*<0.05) and White women (β=-0.036, *p*<0.05).  A cancer diagnosis increased out-of-pocket medical expenses among White men and women but not among Black patients.  The interaction term between cancer and race is not significant among women (b=0.066, standard error [SE]=0.157, *p*>.05).  The adverse effect of cancer on mastery was significantly stronger in White men than Black men (b=0.186, SE=0.092, *p*<0.05).  The effect of hospital stays and, especially, doctor visits on mastery were significantly more adverse in White men than Black men (b=0.185, *p*<0.05).  Out-of-pocket costs increased more for White men than Black men, and these expenses were related to reduced mastery in White men but not Black men. |
|  | Sex | Between-gender comparisons (within the same race) indicate that the adverse effect of cancer on mastery is significantly stronger in White men relative to White women (ATT [White men] − ATT [White women] = −0.045, *p*<0.001). Wealth and debt grew among men, without any significant changes among women. |
| Findings: Strategies for Addressing Differences | Strategies for Addressing Differences | No |
|  | Methods/Instruments | Health and Retirement Study 2006–2014 |
| **Rice, H. E., L’Hotta, A. J., Siječić, A., Drake, B. F., Chang, S. H., Kim, E. H., Wright-Jones, R., Shahid, M., Neal, C., & Housten, A. J. (2024). “I just had to do what I had to do”: Characterizing direct and indirect prostate cancer treatment costs for black survivors and their caregivers. *MDM Policy & Practice*, *9*(2).** | | |
| Study Characteristics | Study Purpose | To characterize the direct and indirect costs associated with prostate cancer (Pca) treatment among Black men and their caregivers to inform shared decision-making materials. |
|  | Source of Data | Qualitative interviews |
|  | Study Type | Qualitative study |
|  | Data Collection Geography | Single institution/site |
|  | Population | Both |
|  | Sample Size | 11 survivors and 11 caregivers |
|  | Medical Condition | Prostate cancer |
| Findings: Categories of PCBEOs | Categories of PCBEOs Examined | Indirect impacts  Intangible burdens |
|  | Indirect Costs | Other indirect impacts: Time handling reorganized budget |
|  | Intangible Burdens | Food insecurity; housing insecurity; reduced spending on basic goods, groceries, and leisure activities; stress and anxiety directly caused by out-of-pocket costs; financial debt; or lost income (i.e., financial toxicity) |
| Findings: Differences in PCBEOs | Types of Differences Examined | Insurance |
|  | Insurance | In the interviews, patients and caregivers with comprehensive insurance coverage (i.e., insurance through the military, Medicare, etc.) described less financial stress and financial challenges. |
| Findings: Strategies for Addressing Differences | Strategies for Addressing Differences | No |
|  | Methods/Instruments | No |
| **Rosenberg, M. W., & Eckstrom, E. (2020). Geographic differences in the unpaid caregiver experience from the National Study of Caregiving (NSOC). *Rural and Remote Health*, *20*(4), 1–8.** | | |
| Study Characteristics | Study Purpose | To identify unpaid caregiver differences in demographics, competing work responsibilities, support, health, caregiver burden, and interaction with health care professionals in U.S. metropolitan and non-metropolitan settings. |
|  | Source of Data | Secondary analysis of pre-existing survey data |
|  | Study Type | Cross-sectional |
|  | Data Collection Geography | National |
|  | Population | Caregivers |
|  | Sample Size | 2,278 caregivers |
|  | Medical Condition | Older adults, general |
| Findings: Categories of PCBEOs | Categories of PCBEOs Examined | Indirect impacts |
|  | Indirect Costs | Other indirect impacts: Missing work for caregiving |
| Findings: Differences in PCBEOs | Types of Differences Examined | Geographic location |
|  | Geographic Location | Caregivers in metropolitan areas compared with caregivers in non-metropolitan areas were significantly more likely to have to miss work for caregiving (*p*<0.05), 35% metropolitan caregivers missed work, and 16.3% of non-metropolitan caregivers missed work for caregiving. |
| Findings: Strategies for Addressing Differences | Strategies for Addressing Differences | No |
|  | Methods/Instruments | National Health Study of Caregiving  National Health and Aging Trends Study |
| **Shah, J. P., Youn, G. M., Wei, E. X., & Patel, Z. M. (2023). Disparities in access to health care in adults with sinusitis in the United States. *International Forum of Allergy & Rhinology*, *13*(11), 2018–2029.** | | |
| Study Characteristics | Study Purpose | To explore disparities in health care access among adults with sinusitis based on sociodemographic factors. |
|  | Source of Data | Secondary analysis of pre-existing survey data |
|  | Study Type | Cross-sectional  Retrospective cohort |
|  | Data Collection Geography | National |
|  | Population | Patients |
|  | Sample Size | 32,994 participants (12.15% with sinusitis) |
|  | Medical Condition | Other: Sinusitis |
| Findings: Categories of PCBEOs | Categories of PCBEOs Examined | Intangible burdens |
|  | Intangible Burdens | Delaying or forgoing medical treatments due to costs |
| Findings: Differences in PCBEOs | Types of Differences Examined | Income, insurance, race/ethnicity |
|  | Income | Compared with patients in the less than $35,000 income bracket, all patients in the higher-income brackets had, overall, increasingly lower risks of delaying or not being able to attain needed health care based on self-report (*p*<0.001 for all groups for delaying care due to cost).  Those in the over $100,000 income bracket report having a lower risk of delaying health care due to transportation (odds ratio [OR]=0.01 [95% confidence interval (CI), 0.001-0.04]) and lower risk of not being able to access medications (OR=0.21 [0.11-0.40]) and follow-up care (OR=0.23 [95% CI, 0.08-0.62]) due to costs. |
|  | Insurance | Compared with individuals with private insurance, individuals on Medicaid (OR=4.64, 95% CI [2.52, 8.55], *p*<0.001) reported a higher risk of delaying care due to a lack of transportation.  Individuals without insurance reported having increased risk of being unable to attain needed medical care (OR=4.80, 95% CI [3.13, 7.37]), prescription medications (OR=4.14, 95% CI [2.76, 6.21], *p*<0.001), specialist care (OR=4.34, 95% CI [2.68, 7.04], *p*=0.000), and follow-up care (OR=2.85, 95% CI [1.77, 4.92]) due to cost.  Individuals on Medicaid reported having lower odds of delaying care (OR=0.37, 95% CI [0.25, 0.56]) and not being able to access care (OR=0.40, 95% CI [0.24, 0.65], *p*<0.001) due to cost. |
|  | Race/Ethnicity | Individuals identifying as being Hispanic (OR=0.51, 95% CI [0.33, 0.77], *p*=0.002) or Black (OR=0.70, 95% CI [0.49, 0.99], *p*=0.045) were associated with lower odds of delaying medical care due to cost compared with those identifying as White. |
| Findings: Strategies for Addressing Differences | Strategies for Addressing Differences | Tailoring prescriptions based on patients’ insurance status and cost barriers so they can get a prescription they can afford. For example, choosing the pharmacy, less-expensive antibiotics, and looking at cost-saving programs such as GoodRx. |
|  | Methods/Instruments | National Health Interview Survey data |
| **Shi, J. J., McGinnis, G. J., Peterson, S. K., Taku, N., Chen, Y. S., Yu, R. K., Wu, C., Mendoza, T. R., Shete, S. S., Ma, H., Volk, R. J., Giordano, S. H., Shih, Y., Nguyen, D., Kaiser, K. W., & Smith, G. L. (2023). Pilot study of a Spanish language measure of financial toxicity in underserved Hispanic cancer patients with low English proficiency. *Frontiers in Psychology*, *14*.** | | |
| Study Characteristics | Study Purpose | To evaluate the acceptability and appropriateness of the Spanish language instrument for assessing financial toxicity (FT) in a pilot sample of un-/under-insured or medically underserved low-English-proficiency Hispanic individuals receiving ambulatory oncology care from a public medical safety net hospital. |
|  | Source of Data | Primary quantitative data collected through a survey, qualitative interviews |
|  | Study Type | Cross-sectional |
|  | Data Collection Geography | Single institution/site |
|  | Population | Patients |
|  | Sample Size | 77 patients |
|  | Medical Condition | Cancer (all types) |
| Findings: Categories of PCBEOs | Categories of PCBEOs Examined | Direct medical costs  Indirect impacts  Intangible burdens |
|  | Direct Medical Costs | Medical financial debt |
|  | Indirect Costs | Unpaid time off work, early retirement |
|  | Intangible Burdens | Family/social life impacts; food insecurity; housing insecurity; reduced spending on basic goods, groceries, and leisure activities; stress and anxiety directly caused by out-of-pocket costs, financial debt, or lost income (i.e., FT) |
| Findings: Differences in PCBEOs | Types of Differences Examined | Insurance |
|  | Insurance | In the multivariable models un/under-insured English-speaking (odds ratio [OR]=5.73, *p*=0.001, 95% CI [1.49, 22.1]) and un/underinsured Spanish-speaking Hispanic patients (OR=4.00, *p*=0.04, 95% CI [1.07, 14.93]) had higher odds of coping domain FT than English-speaking insured Hispanic patients.  Un/underinsured English-speaking patients also had higher odds of overall FT compared with English-speaking insured Hispanic patients (OR=4.13, *p*=0.03, 95% CI [1.13, 15.12]). |
| Findings: Strategies for Addressing Differences | Strategies for Addressing Differences | No |
|  | Methods/Instruments | Spanish version of the Economic Strain and Resilience in Cancer (ENRICh) |
| **Simmons, M., Bishu, K. G., Williams, J. S., Walker, R. J., Dawson, A. Z., & Egede, L. E. (2019). Racial and ethnic differences in out-of-pocket expenses among adults with diabetes. *Journal of the National Medical Association*, *111*(1), 28–36.** | | |
| Study Characteristics | Study Purpose | To assess racial and ethnic differences in out-of-pocket (out-of-pocket) costs among a nationally representative sample of adults with diabetes. |
|  | Source of Data | Secondary analysis of pre-existing survey data |
|  | Study Type | Cross-sectional |
|  | Data Collection Geography | National |
|  | Population | Patients |
|  | Sample Size | 17,702 adults |
|  | Medical Condition | Diabetes |
| Findings: Categories of PCBEOs | Categories of PCBEOs Examined | Direct medical costs |
|  | Direct Medical Costs | Out-of-pocket medical costs |
| Findings: Differences in PCBEOs | Types of Differences Examined | Age, education, geographic location, income, insurance, race/ethnicity, sex/gender |
|  | Age | Compared with respondents younger than 45, respondents aged 45–64 (β=299, *p*<0.001, 95% confidence interval [CI] 146.90, 453.00]) and 65 or older (β=559, *p*<0.001, 95% CI [375.94, 742.18]) reported higher out-of-pocket expenditures. |
|  | Educational Attainment | High school (β=116, *p*=0.049, 95% CI [0.33, 233.63]) and college (β=4.97, *p*<0.001, 95% CI [328.22, 631.53]) are associated with higher out-of-pocket expenditures. |
|  | Geographic Location | Compared with the Northeast, the Midwest (β=1.93, *p*=0.009, 95% CI [48.64, 338.59]), South (β=309, *p*<0.001, 95% CI [181.58, 437.33]), and West (β=165, *p*=0.041, 95% CI [6.45, 324.68]) are associated with higher out-of-pocket expenditures. |
|  | Income | Compared with low-income respondents, middle-income (β=189, *p*=0.010, 95% CI [44.43, 334.88]) and high-income (β=343, *p*<0.001, 95% CI [170.06, 517.49]) respondents were associated with higher out-of-pocket expenditures. |
|  | Insurance | Public insurance was associated with lower out-of-pocket expenditures (β=-166, *p*=0.013, 95% CI [-298.63, -35.28]).  Private insurance was associated with higher out-of-pocket expenditures (β=962, *p*<0.001, 95% CI [642.18, 1282.23]). |
|  | Race/Ethnicity | Compared with White respondents, non-Hispanic Black (β=-481, *p*<0.001, 95% CI [-621.30, -341.51]), Hispanic (β=-591, *p*<0.001, 95% CI [-727.38, -455.0]), and other (β=-643, *p*<0.001, 95% CI [-803.07, -484.47]) respondents had lower out-of-pocket expenditures. |
|  | Sex | Female respondents reported higher out-of-pocket expenditures compared with males (β=273, *p*<0.001, 95% CI [155.53, 392.15]). |
| Findings: Strategies for Addressing Differences | Strategies for Addressing Differences | No |
|  | Methods/Instruments | Medical Expenditure Panel Survey Household Component from 2002 to 2011 |
| **Smith, G. L., Smith, B. D., Wu, C. F., Shaitelman, S. F., Chavez-MacGregor, M., Murthy, R., Kaiser, K., Ku, K. S., Shi, J. J., Shete, S. S., Chen, Y., Volk, R. J., Giordano, S. H., Shih, Y., & Hoffman, K. E. (2024). Financial toxicity in breast cancer patients receiving regional nodal irradiation: Variation by cancer subtype. *The Breast*, *78*, 103813.** | | |
| Study Characteristics | Study Purpose | To evaluate sociodemographic and clinical predictors of financial toxicity (FT) among patients with breast cancer with higher-risk clinical factors warranting regional nodal irradiation. |
|  | Source of Data | Primary quantitative data collected through a survey |
|  | Study Type | Cross-sectional |
|  | Data Collection Geography | Single institution/site |
|  | Population | Patients |
|  | Sample Size | 125 patients |
|  | Medical Condition | Breast cancer |
| Findings: Categories of PCBEOs | Categories of PCBEOs Examined | Intangible burdens |
|  | Intangible Burdens | Stress and anxiety directly caused by out-of-pocket costs, financial debt, or lost income (i.e., FT) |
| Findings: Differences in PCBEOs | Types of Differences Examined | Age, education, race/ethnicity |
|  | Age | Compared with patients diagnosed at 60 or older, patients diagnosed at 40–49 and 50–59 had a higher relative risk (RR) of FT (RR=2.73, *p*=0.05, 95% confidence interval [CI] [0.98, 4.28] and RR=2.88, *p*=0.04, 95% CI [1.08, 4.63], respectively). |
|  | Educational Attainment | Patients with high school to some college had a higher RR of FT compared with patients with college and above (RR=2.64, *p*=0.01, 95% CI [1.24, 4.37]). |
|  | Race/Ethnicity | Black/Hispanic/other patients had higher RR of FT compared with White patients (RR=1.77, *p*=0.02, 95% CI [11.11, 2.24]). |
| Findings: Strategies for Addressing Differences | Strategies for Addressing Differences | No |
|  | Methods/Instruments | Economic Strain and Resilience in Cancer (ENRICh) was used to estimate FT: Validated |
| **Starr, L. T., Bullock, K., Washington, K., Aryal, S., Parker Oliver, D., & Demiris, G. (2022). Anxiety, depression, quality of life, caregiver burden, and perceptions of caregiver-centered communication among black and white hospice family caregivers. *Journal of Palliative Medicine*, *25*(4), 596–605.** | | |
| Study Characteristics | Study Purpose | To describe demographics and caregiving experiences (e.g., duration of caregiving, weekly hours caregiving) by racial group and to understand whether anxiety, depression, quality of life (QOL), caregiver burden, and perceptions of hospice team communication differ between Black and White hospice family caregivers. |
|  | Source of Data | Primary quantitative data collected through a survey |
|  | Study Type | Cross-sectional |
|  | Data Collection Geography | Multiple sites across multiple states |
|  | Population | Caregivers |
|  | Sample Size | 722 caregivers |
|  | Medical Condition | End-of-life patients, general |
| Findings: Categories of PCBEOs | Categories of PCBEOs Examined | Intangible burdens |
|  | Intangible Burdens | Fear and anxiety about future ability to afford medical treatments, hindered career or education advancement, QOL impacts (not quality-adjusted life-years or disability-adjusted life-years) |
| Findings: Differences in PCBEOs | Types of Differences Examined | Race/ethnicity |
|  | Race/Ethnicity | Black and White caregivers differed across demographic and socioeconomic variables.  White caregivers reported lower QOL than Black caregivers (*p*=0.04), specifically in emotional (*p*=0.02) and social (*p*=0.0005) domains.  Black caregivers were significantly more likely to report employment-related changes as a result of caring for a patient on hospice (Black caregivers, 41%; White caregivers, 38.35%, *p*=0.02). |
| Findings: Strategies for Addressing Differences | Strategies for Addressing Differences | No |
|  | Methods/Instruments | Zarit Burden Interview Guide  Caregiver-Centered Communication Questionnaire  Caregiver Quality of life Index-Revised  Patient Health Questionnaire 9-item  Generalized Anxiety Disorder 7-item scale |
| **Teagarden, D. L., Morton, M. L., Janocko, N. J., Groover, O., Villarreal, H. K., Evans, L., Drane, D. L., & Karakis, I. (2020). Socioeconomic disparities in patients with psychogenic nonepileptic seizures and their caregivers. *Epilepsy & Behavior*, *110*, 107160.** | | |
| Study Characteristics | Study Purpose | To characterize any socioeconomic disparities in patients with psychogenic nonepileptic seizures and their caregivers with the intent to identify potential intervention targets to ameliorate them in the future. |
|  | Source of Data | Primary quantitative data collected through a survey |
|  | Study Type | Cross-sectional |
|  | Data Collection Geography | Single institution/site |
|  | Population | Caregivers |
|  | Sample Size | 28 caregivers |
|  | Medical Condition | Other: Seizures |
| Findings: Categories of PCBEOs | Categories of PCBEOs Examined | Indirect impacts |
|  | Indirect Costs | Time spent providing unpaid caregiving |
| Findings: Differences in PCBEOs | Types of Differences Examined | Income |
|  | Income | Caregivers with high socioeconomic status (SES) have increased caregiving burden compared with those with low SES (*p*=0.01). |
| Findings: Strategies for Addressing Differences | Strategies for Addressing Differences | No |
|  | Methods/Instruments | Zarit Caregiver Burden Inventory: Validated |
| **Tucker, K., Dark, T., & Harman, J. S. (2018). Variation in out-of-pocket health care costs for individuals with anxiety disorders by type of insurance coverage. *Journal of Anxiety Disorders*, *58*, 18–22.** | | |
| Study Characteristics | Study Purpose | To determine the annual out-of-pocket (out-of-pocket) cost burden faced by individuals with anxiety disorders and the variation of these costs by type of insurance coverage. |
|  | Source of Data | Secondary analysis of pre-existing survey data |
|  | Study Type | Cross-sectional |
|  | Data Collection Geography | National |
|  | Population | Patients |
|  | Sample Size | 9,985 patients |
|  | Medical Condition | Other: Anxiety disorder |
| Findings: Categories of PCBEOs | Categories of PCBEOs Examined | Direct medical costs |
|  | Direct Medical Costs | Out-of-pocket medical costs |
| Findings: Differences in PCBEOs | Types of Differences Examined | Insurance |
|  | Insurance | Those who are uninsured spent an average of 12.5% of their annual income on medical expenses, a significantly higher percentage of income among all the insurance categories (*p*<0.01). |
| Findings: Strategies for Addressing Differences | Strategies for Addressing Differences | No |
|  | Methods/Instruments | Medical Expenditure Panel Survey |
| **Valero-Elizondo, J., Javed, Z., Khera, R., Tano, M. E., Dudum, R., Acquah, I., Hyder, A. A., Andrieni, J., Sharma, G., Blaha, M. J., Virani, S. S., Blankstein, R., Cainzos-Achirica, M., & Nasir, K. (2022). Unfavorable social determinants of health are associated with higher burden of financial toxicity among patients with atherosclerotic cardiovascular disease in the US: Findings from the National Health Interview Survey. *Archives of Public Health*, *80*(1), 248.** | | |
| Study Characteristics | Study Purpose | (1) To fill the knowledge gap using large-scale, population-based, nationally representative data, and (2) to assess whether these associations vary across age strata. |
|  | Source of Data | Secondary analysis of pre-existing survey data |
|  | Study Type | Cross-sectional |
|  | Data Collection Geography | National |
|  | Population | Patients |
|  | Sample Size | 15,578 patients |
|  | Medical Condition | Cardiovascular disease (i.e., heart disease) |
| Findings: Categories of PCBEOs | Categories of PCBEOs Examined | Intangible burdens |
|  | Intangible Burdens | Delaying or forgoing medical treatments due to costs, other intangible burdens: Difficulty paying medical bills, cost-related medication non-adherence |
| Findings: Differences in PCBEOs | Types of Differences Examined | Income |
|  | Income | Burden of having more than two financial toxicity components was highest in participants with low income. |
| Findings: Strategies for Addressing Differences | Strategies for Addressing Differences | No |
|  | Methods/Instruments | No |
| **Vanderpool, R. C., Chen, Q., Johnson, M. F., Lei, F., Stradtman, L. R., & Huang, B. (2020). Financial distress among cancer survivors in Appalachian Kentucky. *Cancer Reports*, *3*(2), e1221.** | | |
| Study Characteristics | Study Purpose | To estimate the prevalence of financial distress among cancer survivors residing in Appalachian Kentucky, with a specific focus on the impact of rurality and to elucidate the risk factors impacting financial distress among this population. |
|  | Source of Data | Primary quantitative data collected through a survey; secondary analysis of pre-existing survey data |
|  | Study Type | Cross-sectional |
|  | Data Collection Geography | National |
|  | Population | Patients |
|  | Sample Size | 504 patients (secondary sample), 179 patients (primary sample) |
|  | Medical Condition | Cancer (all types) |
| Findings: Categories of PCBEOs | Categories of PCBEOs Examined | Intangible burdens |
|  | Intangible Burdens | Stress and anxiety directly caused by out-of-pocket costs, financial debt, or lost income (i.e., financial toxicity) |
| Findings: Differences in PCBEOs | Types of Differences Examined | Age, income, sex/gender |
|  | Age | Younger and middle-aged respondents were almost 5 times more likely to report financial hardship compared with survivors aged 65 or older (*p*=.006). |
|  | Income | Cancer survivors reporting an annual household income of less than $19,999 were 20 times more likely to report financial distress compared with the reference category of $100,000 or more (*p*=0.001).  Respondents with an annual household income of less than $19,999 were 4 times more likely to report cancer-related financial distress compared with survivors earning $100,000 or more (*p*=.005). |
|  | Sex | Male cancer survivors in Appalachian Kentucky were 3 times more likely to report financial distress compared with females (*p*=.015). |
| Findings: Strategies for Addressing Differences | Strategies for Addressing Differences | There is an obvious need for increased financial risk and cost of care conversations between cancer patients and their health care team; ideally, these conversations would promote shared decision making and improved cost-related health literacy among patients.  In addition, support services such as financial and insurance counseling, patient navigation, medication assistance programs, social services, and psychosocial oncology resources are needed to help patients manage medical and non-medical financial issues related to their cancer care.  These services and resources are needed within clinical and community settings but could also be made available through connected health programs, particularly for isolated rural and Appalachian communities. |
|  | Methods/Instruments | No |
| **Vasquez-Trespalacios, E. M., Rivera, J. N., McIntyre, M., Santiago-Datil, W., Wenham, R. M., Vadaparampil, S. T., Buras, A. L., & Conley, C. C. (2024). High financial hardship among patients with advanced ovarian cancer: Financial toxicity in advanced ovarian cancer patients. *Journal of Social Work in End-of-Life & Palliative Care*, *20*(2), 120–132.** | | |
| Study Characteristics | Study Purpose | To examine the experience of, and factors associated with, financial toxicity (FT) among patients diagnosed with advanced ovarian cancer. |
|  | Source of Data | Secondary analysis of pre-existing survey data |
|  | Study Type | Cross-sectional |
|  | Data Collection Geography | Single institution/site |
|  | Population | Patients |
|  | Sample Size | 38 patients |
|  | Medical Condition | Ovarian cancer |
| Findings: Categories of PCBEOs | Categories of PCBEOs Examined | Intangible burdens |
|  | Intangible Burdens | Stress and anxiety directly caused by out-of-pocket costs, financial debt, or lost income (i.e., FT) |
| Findings: Differences in PCBEOs | Types of Differences Examined | Employment status, income |
|  | Employment Status | Employment status was significantly associated with clinically relevant FT (*p*=0.001). |
|  | Income | Participants with a yearly household income less than $50,000, who were born outside the United States and unable to work were associated with clinically significant FT. Number of people (standard deviation) among those with income below $50,000: Low FT=5 (38.8), high FT=8 (61.5), income above $50,0000: Low FT=18 (94.7), high FT=1 (5.3), *p*=0.001. |
| Findings: Strategies for Addressing Differences | Strategies for Addressing Differences | No |
|  | Methods/Instruments | Comprehensive Score for FT |
| **Wajnberg, A., Soones, T. N., Smith, K. L., Russell, D., Ross, J. S., & Federman, A. (2016). Identifying sociodemographic characteristics associated with burden among caregivers of the urban homebound: The importance of racial and relationship differences. *Gerontology and Geriatric Medicine*, *2*.** | | |
| Study Characteristics | Study Purpose | To examine patient and caregiver characteristics (e.g., race/ethnicity, health status) associated with caregiver burden. |
|  | Source of Data | Primary quantitative data collected through a survey |
|  | Study Type | Cross-sectional |
|  | Data Collection Geography | Multiple sites in a single state |
|  | Population | Caregivers |
|  | Sample Size | 49 caregivers |
|  | Medical Condition | Older adults, general |
| Findings: Categories of PCBEOs | Categories of PCBEOs Examined | Intangible burdens |
|  | Intangible Burdens | Other intangible burdens: Composite caregiver burden |
| Findings: Differences in PCBEOs | Types of Differences Examined | Race/ethnicity |
|  | Race/Ethnicity | Compared with non-Hispanic White patients (36.5), non-Hispanic Black (24.5) and Hispanic (23.0) patients had higher average caregiver burden scores (*p*=0.02). |
| Findings: Strategies for Addressing Differences | Strategies for Addressing Differences | No |
|  | Methods/Instruments | Zarit Caregiver Burden Scale |
| **Washington, K. T., Pike, K. C., Demiris, G., Parker Oliver, D., Albright, D. L., & Lewis, A. M. (2015). Gender differences in caregiving at end of life: Implications for hospice teams. *Journal of Palliative Medicine*, *18*(12), 1048–1053.** | | |
| Study Characteristics | Study Purpose | To determine how, if at all, current day caregiving at end of life varies by gender. |
|  | Source of Data | Secondary analysis of pre-existing survey data |
|  | Study Type | Cross-sectional |
|  | Data Collection Geography | Multiple sites in a single state |
|  | Population | Patients |
|  | Sample Size | 289 caregivers |
|  | Medical Condition | Other: Patients in hospice care |
| Findings: Categories of PCBEOs | Categories of PCBEOs Examined | Direct non-medical costs  Intangible burdens |
|  | Direct Non-Medical Costs | Other direct non-medical costs: General impact on finances |
|  | Intangible Burdens | Other intangible burdens: Scheduling changes |
| Findings: Differences in PCBEOs | Types of Differences Examined | Age, race/ethnicity |
|  | Age | Age was inversely associated with caregiving burden’s impact on schedule, such that older individuals experienced significantly less caregiving burden due to scheduling (β=-0.02, 95% confidence interval [CI] [-0.02, -0.01], *p*≤0.01). |
|  | Race/Ethnicity | White race was inversely associated with caregiving burden’s impact on finances, such that White individuals experienced significantly less association between caregiver reactions and financial burden (β=-0.44, 95% CI= [-0.83, -0.04], *p*=0.03). |
| Findings: Strategies for Addressing Differences | Strategies for Addressing Differences | Interventions consistent with a feminist perspective on family care may involve helping female caregivers access support from their social network of family members and friends, who may be less likely to perceive women as needing help in their caregiving role than men or who may be more likely to provide unsolicited critiques of caregiving provided by women, who are often assumed to be ‘‘natural’’ caregivers.  This may involve structured interventions, such as assertiveness training or communication skills education, but it may also be accomplished through the provision of peer support.  For example, caregivers may benefit from hearing how others have secured tangible support by taking advantage of even vague offers of help (e.g., ‘‘If there’s ever anything I can do’’) from family members and friends. |
|  | Methods/Instruments | Caregiver Reaction Assessment |
| **Waters, A. R., Kent, E. E., Meernik, C., Getahun, D., Laurent, C. A., Xu, L., Mitra, S., Kushi, L. H., Chao, C., & Nichols, H. B. (2023). Financial hardship differences by LGBTQ+ status among assigned female at birth adolescent and young adult cancer survivors: A mixed methods analysis. *Cancer Causes & Control*, *34*(11), 973–981.** | | |
| Study Characteristics | Study Purpose | To use qualitative and quantitative survey data from the Horizon Study cohort to assess financial hardship of adolescent and young adults (AYAs) by LGBTQ+ status. |
|  | Source of Data | Primary quantitative data collected through a survey  Other: Qualitative survey questions. |
|  | Study Type | Cross-sectional |
|  | Data Collection Geography | Multiple sites across multiple states |
|  | Population | Patients |
|  | Sample Size | 1,679 patients |
|  | Medical Condition | Breast cancer, gynecologic cancer, leukemia and lymphoma, thyroid cancer, melanoma |
| Findings: Categories of PCBEOs | Categories of PCBEOs Examined | Direct medical costs  Direct non-medical costs  Intangible burdens |
|  | Direct Medical Costs | Medical financial debt |
|  | Direct Non-Medical Costs | Filed for bankruptcy |
|  | Intangible Burdens | Reduced spending on basic goods, groceries, and leisure activities; stress and anxiety directly caused by out-of-pocket costs, financial debt, or lost income (i.e., financial toxicity) |
| Findings: Differences in PCBEOs | Types of Differences Examined | Other (LGBTQ+ status) |
|  | Other | LGBTQ+ status: In bivariate analyses, LGBTQ+ AYAs experienced significantly more material financial hardship (41% vs. 26%, *p*=0.004) and psychological financial hardship (61% vs. 48%, *p*=0.03) than non-LGBTQ+AYAs. |
| Findings: Strategies for Addressing Differences | Strategies for Addressing Differences | No |
|  | Methods/Instruments | Self-designed questionnaire using questions from the Medical Expenditure Panel Survey experiences with cancer survivorship supplement |
| **Zahnd, W. E., Davis, M. M., Rotter, J. S., Vanderpool, R. C., Perry, C. K., Shannon, J., Ko, L. K., Wheeler, S. B., Odahowski, C. L., Farris, P. E., & Eberth, J. M. (2019). Rural-urban differences in financial burden among cancer survivors: An analysis of a nationally representative survey. *Supportive Care in Cancer*, *27*(12), 4779–4786.** | | |
| Study Characteristics | Study Purpose | To examine rural–urban differences in reported financial problems due to cancer using a nationally representative survey. |
|  | Source of Data | Secondary analysis of pre-existing survey data |
|  | Study Type | Cross-sectional |
|  | Data Collection Geography | National |
|  | Population | Patients |
|  | Sample Size | 1,368 patients |
|  | Medical Condition | Cancer (all types) |
| Findings: Categories of PCBEOs | Categories of PCBEOs Examined | Direct medical costs  Intangible burdens |
|  | Direct Medical Costs | Medical financial debt, out-of-pocket medical costs |
|  | Intangible Burdens | Delaying or forgoing medical treatments due to costs |
| Findings: Differences in PCBEOs | Types of Differences Examined | Geographic location, income |
|  | Geographic Location | Of rural cancer survivors, 50.4% indicated financial problems after their diagnosis and treatment compared with 38.8% of urban survivors (difference=11.6%, *p*=0.02). |
|  | Income | Reported financial problems increased with decreasing income levels; 29.7% of cancer survivors making $75,000 or more reported financial problems compared with 55.2% at the lowest income level ($0–$19,999) (*p*=0.04). |
| Findings: Strategies for Addressing Differences | Strategies for Addressing Differences | No |
|  | Methods/Instruments | National Cancer Institute’s Health Information National Trends Survey |
| **Zheng, Z., Jemal, A., Han, X., Guy Jr, G. P., Li, C., Davidoff, A. J., Banegas, M. P., Ekwueme, D. U., & Yabroff, K. R. (2019). Medical financial hardship among cancer survivors in the United States. *Cancer*, *125*(10), 1737–1747.** | | |
| Study Characteristics | Study Purpose | To examine medical financial hardship in cancer survivors and those without a cancer history in the United States. |
|  | Source of Data | Secondary analysis of pre-existing survey data |
|  | Study Type | Cross-sectional |
|  | Data Collection Geography | National |
|  | Population | Patients |
|  | Sample Size | 134,790 respondents |
|  | Medical Condition | Cancer (all types) |
| Findings: Categories of PCBEOs | Categories of PCBEOs Examined | Direct medical costs  Intangible burdens |
|  | Direct Medical Costs | Medical financial debt |
|  | Intangible Burdens | Delaying or forgoing medical treatments due to costs, stress and anxiety directly caused by out-of-pocket costs, financial debt, or lost income (i.e., financial toxicity) |
| Findings: Differences in PCBEOs | Types of Differences Examined | Insurance |
|  | Insurance | Among cancer survivors aged 18–49, those with high-deductible health plans without health savings accounts were more likely to report any material hardship (60% vs. 36.8%, *p*<0.05) and behavioral hardship (34.6% vs. 20.3%, *p*<0.05). |
| Findings: Strategies for Addressing Differences | Strategies for Addressing Differences | No |
|  | Methods/Instruments | National Health Interview Survey |
